# Discovery of 4,5,6,7-Tetrahydrothieno [3,2-b] Pyridine as Novel Fungicide Lead Scaffold

**DOI:** 10.3390/microorganisms13071588

**Published:** 2025-07-05

**Authors:** Ke Chen, Difan Deng, Yupeng Yin, Dongmei Xi, Phumbum Park, Wei Gao, Rui Liu, Kang Lei

**Affiliations:** 1 Department of Biotechnology, The University of Suwon, Hwaseong 18323, Gyeonggi-do, Republic of Korea; chenke_sd@163.com (K.C.); pbpark@suwon.ac.kr (P.P.); 2 College of Life Sciences, Linyi University, Linyi 276005, China; xidongmei@lyu.edu.cn; 3 School of Pharmaceutical Sciences and Food Engineering, Liaocheng University, Liaocheng 252059, China; 2022406865@stu.lcu.edu.cn (D.D.); 2022406950@stu.lcu.edu.cn (Y.Y.); gaowei@lcu.edu.cn (W.G.)

**Keywords:** 4,5,6,7-tetrahydrothieno [3,2-b] pyridine, fungicides, 4,5-dihydrotetrazolo [1,5-a]thieno [2,3-e]pyridine, mode of action

## Abstract

To identify fungicide lead compounds with novel scaffold and high efficacy, a library of 4,5-dihydrotetrazolo [1,5-a]thieno [2,3-e]pyridine derivatives, consisting of 10 newly synthesized compounds and 12 previously reported compounds, was evaluated for their potential as fungicide agents. In vitro bioassay results indicated that some target compounds exhibited certain antifungal activity against the tested fungi at a concentration of 50 μg/mL. Especially, compounds **I-1**, **I-5**, **I-7**, and **I-12** demonstrated promising antifungal activity against *C. arachidicola*, *R. solani*, and *S. sclerotiorum*, with EC_50_ values ranging from 4.61 to 6.66 μg/mL. Additionally, transcriptome analysis revealed that the molecular mode of action of compound **I-12** involves the inhibition of nitrogen metabolism and the proteasome pathway. The present work demonstrates that 4,5,6,7-tetrahydrothieno [3,2-b] pyridine represents a promising lead scaffold and provides important theoretical foundations and innovative perspectives for the development of novel and highly efficient fungicides.

## 1. Introduction

As an indispensable tool for the prevention and control of diseases, pests, and weeds in agricultural production, pesticides play a critical role in ensuring stable agricultural yields and improving economic efficiency [1,2,3]. However, the development of new pesticides faces significant challenges, including a lengthy development cycle, substantial financial investment, and low success rates [4]. Identifying novel mode of action and lead scaffolds for pesticides constitutes a critical component in the development of new pesticides [5,6,7,8,9]. On the one hand, the discovery of innovative targets offers precise research directions for advancing pesticide development. Currently, cutting-edge technologies such as drug affinity responsive target stability (DARTS) [10], cellular thermal shift assay (CETSA) [11,12], thermal proteome profiling (TPP) [13,14], stability of proteins from rates of oxidation (SPROX) [15], and target identification by chromatographic elution (TICE) [16] have significantly facilitated the identification of multiple pesticide targets, including the pyruvate dehydrogenase complex [17], transketolase [18], pyruvate kinase [19], and dihydroorotate dehydrogenase [20], among others. Integrating novel targets with advanced computer-aided drug design methodologies, such as structure-based and ligand-based drug design, can substantially enhance the efficiency and effectiveness of new pesticide development. On the other hand, testing the biological activity of a compound on different models increases the probability of discovering lead scaffolds or candidate new pesticides [21], thereby improving the efficiency of discovery and development, and playing a crucial role in the development of new pesticides.

**Figure 1:**
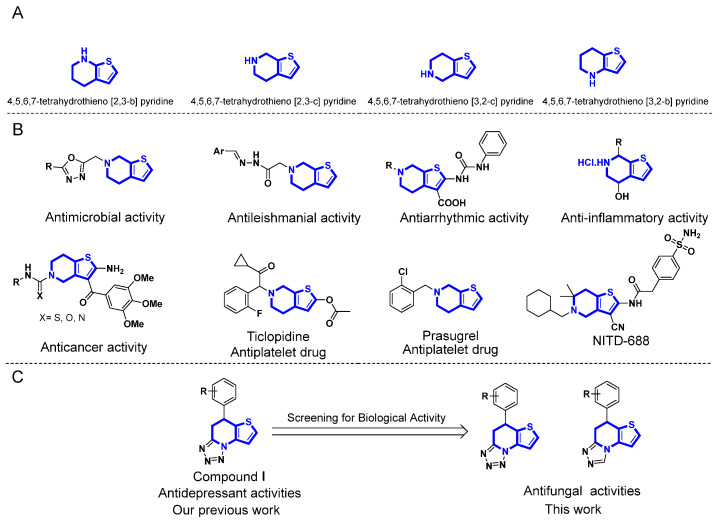
The structures of 4,5,6,7-tetrahydrothienopyridines. (**A**) the common structures of four representative 4,5,6,7-tetrahydrothienopyridines; (**B**) the structures of representative 4,5,6,7-tetrahydrothienopyridines with biological activity; (**C**) the design of target compound.

4,5,6,7-Tetrahydrothienopyridines are an important class of heterocyclic compounds that are widely distributed in nature and serve as critical structural motifs in bioorganic chemistry (Figure 1A). Many 4,5,6,7-tetrahydrothienopyridine derivatives have been synthesized and evaluated for biological activities (Figure 1B), such as anti-rheumatoid arthritis [22], anti-inflammatory [23], antiviral [24], antioxidant [25], antiplatelet [26], antitumor [27], and antimicrobial activities [28]. Furthermore, several drug molecules containing the 4,5,6,7-tetrahydrothienopyridine scaffold are currently available on the market, while others are at different stages of clinical development [29]. In our previous research, a series of 4,5,6,7-tetrahydrothieno [3,2-b] pyridine derivatives (Figure 1C, compound **I**) were synthesized and assessed for their antidepressant activity [30,31]. However, the efficacy of most compounds was found to be suboptimal. Notably, compound **I** incorporates structural moiety such as thiophene and tetrazole, which have been reported to possess potential inhibitory effects against plant pathogenic fungi [32,33,34,35]. Consequently, we constructed a library of 4,5,6,7-tetrahydrothieno [3,2-b] pyridine derivatives, comprising 10 newly synthesized compounds and 12 previously reported ones, and evaluated their inhibitory effects against plant pathogenic fungi. Herein, we report on the antifungal activity, structure–activity relationships, and the preliminary molecular mode of action of these 4,5,6,7-tetrahydrothieno [3,2-b] pyridine derivatives. To the best of our knowledge, this is the first investigation into the effect of 4,5,6,7-tetrahydrothieno [3,2-b] pyridine derivatives against plant pathogenic fungi.

## 2. Materials and Methods

### 2.1. General Information

The original raw materials were purchased from Shanghai Energy Chemical (Shanghai, China), Tokyo Chemical Industry (Tokyo, Japan), or Shanghai Aladdin (Shanghai, China). All reagents were of an analytical reagent grade and used as received if not specified otherwise. Column chromatography purification was carried out using silica gel column chromatography (silica gel 300~400 mesh) (Qingdao Makall Group Co., Ltd., Qingdao, China). Melting points were determined on an X-4 electrothermal digital melting point apparatus (Beijing Tech Instruments Co., Beijing, China) and uncorrected. ^1^H NMR and ^13^C NMR spectra were recorded using a AV500 spectrometer (Bruker Co., Fallanden, Switzerland) in CDCl_3_ or DMSO-d_6_ with TMS as an internal standard, and chemical shift values (δ) were given in parts per million (ppm). High-resolution mass spectra (HRMS) were recorded using a 6520 TOF LC/MS (Agilent Technologies, Santa Clara, CA, USA) instrument.

### 2.2. Chemical Synthesis Procedures

**Scheme 1:**
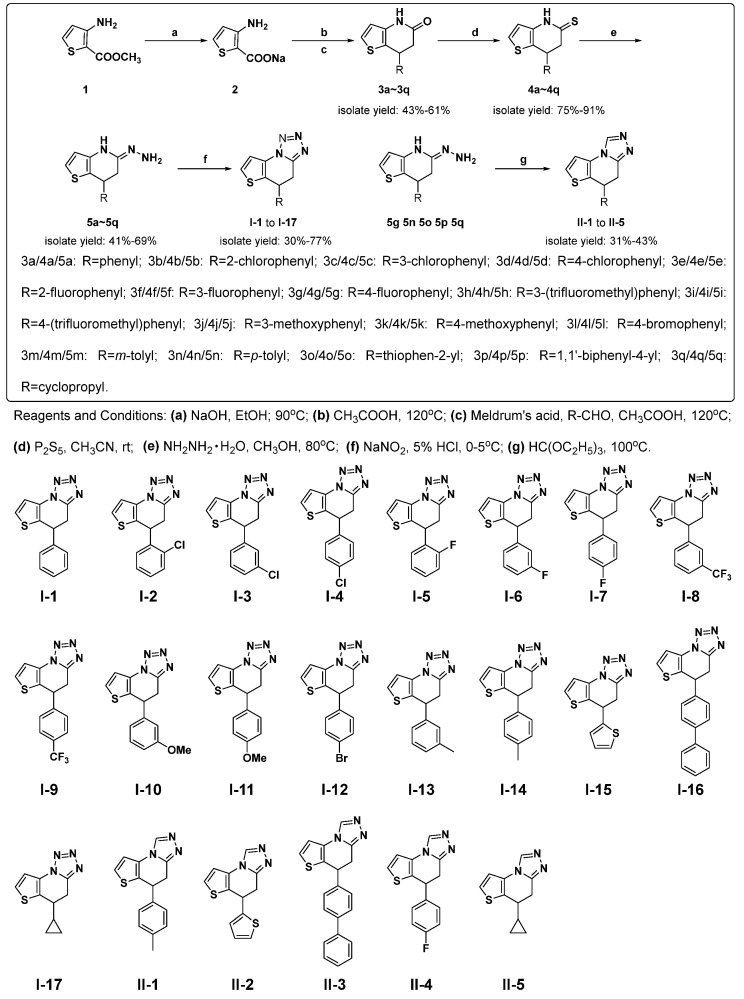
General synthetic route of series target compounds **I** and **II**.

The synthetic pathway employed for the preparation of the series target compounds **I** and **II** is illustrated in Scheme 1. It should be noted that the yields have not been optimized.

#### 2.2.1. General Method for the Synthesis of Intermediates **3a**~**3q**

Intermediates **3a**~**3q** were synthesized according to the previously reported methods [30,31]. Methyl-3-aminothiophene-2-carboxylate (1.57 g, 10 mmol), NaOH (0.8 g, 20 mmol), and 40 mL of ethanol were sequentially added to a 100 mL round-bottom flask. The mixture was then stirred at 80 °C for 12 h, and the reaction was monitored by thin layer chromatography (TLC). After the reaction was completed, the solvent was removed under reduced pressure to give the intermediate **2**, which was followed dissolved in 30 mL of acetic acid and stirred at 120 °C for 1 h. Subsequently, meldrum’s acid (2.16 g, 15 mmol) and benzaldehyde (1.17 g, 11 mmol) were sequentially added into the above solution. The mixture was then stirred at 120 °C for 12 h. After the reaction was completed (monitored by TLC), 50 mL of water was added to the reaction solution and then extracted with ethyl acetate (EtOAc, 50 mL × 3). The organic phase was separated, washed three times with distilled water, and then the organic phase was dried with anhydrous Na_2_SO_4_ for 2 h, and concentrated, and the residue was purified by 300~400 mesh silica gel column chromatography using dichloromethane/methanol (100:1, *v*/*v*) as the eluent to obtain intermediate **3a** as a white solid (1.3 g, yield 57%). ^1^H NMR (500 MHz, CDCl_3_) δ 8.45 (s, 1H), 7.36–7.33 (m, 2H), 7.31–7.25 (m, 3H), 7.14 (d, J = 5.3 Hz, 1H), 6.70 (d, J = 5.3 Hz, 1H), 4.52–4.28 (m, 1H), 3.05–2.88 (m, 2H); ^13^C NMR (125 MHz, CDCl_3_) δ 170.77, 142.18, 135.96, 128.98, 127.57, 127.26, 124.52, 120.15, 117.85, 40.02, 39.13. (Figures S1 and S2) The intermediates **3b**~**3q** were prepared by a procedure like that for **3a**.

#### 2.2.2. General Method for the Synthesis of Intermediates **4a**~**4q**

A 100 mL round-bottom flask was charged with 50 mL of a mixed solvent composed of acetonitrile and triethylamine (3:1, *v*/*v*). Subsequently, phosphorus pentasulfide (1.0 g, 4.5 mmol) was added at 0 °C under stirring. Intermediate **3a** (1.15 g, 5 mmol) was then introduced into the reaction mixture, and the resulting solution was stirred at 70 °C for 12 h. Upon completion of the reaction (monitored by TLC), the solvent was removed under reduced pressure. The crude product was purified by crystallization using dichloromethane, yielding intermediate **4a** as a yellow solid (1.1 g, yield 89%). ^1^H NMR (500 MHz, DMSO) δ 12.42 (s, 1H), 7.40 (d, J = 5.3 Hz, 1H), 7.33 (dd, J = 10.2, 4.6 Hz, 2H), 7.27–7.22 (m, 3H), 6.87 (d, J = 5.3 Hz, 1H), 4.38 (t, J = 7.3 Hz, 1H), 3.36–3.23 (m, 2H); ^13^C NMR (125 MHz, DMSO) δ 196.56, 142.86, 136.46, 129.21, 127.65, 127.58, 125.29, 124.06, 118.39, 48.35, 37.73. (Figures S3 and S4) Intermediates **4b**~**4q** were synthesized following a similar procedure.

#### 2.2.3. General Method for the Synthesis of Intermediates **5a**~**5q**

To a 100 mL round-bottom flask, intermediate **4a** (980 mg, 4.0 mmol) was added, followed by the sequential addition of methanol (20 mL) and hydrazine hydrate (400 mg, 8.0 mmol). The reaction mixture was stirred at 70 °C for 1 h. Upon completion of the reaction (monitored by TLC), the solvent was removed under reduced pressure. The crude product was subsequently purified by crystallization from methanol, yielding intermediate **5a** as a white solid (638 mg, yield 62%). ^1^H NMR (500 MHz, DMSO) δ 7.31–7.28 (m, 2H), 7.25–7.21 (m, 2H), 7.21–7.18 (m, 2H), 6.78 (d, J = 5.2 Hz, 1H), 4.22 (t, J = 6.5 Hz, 1H), 2.79–2.57 (m, 2H); ^13^C NMR (125 MHz, DMSO) δ 144.36, 128.94, 127.71, 127.21, 123.88, 119.60, 116.11, 38.78, 36.69. (Figures S5 and S6) Intermediates **5a**~**5q** were synthesized following a similar procedure.

#### 2.2.4. General Method for the Synthesis of Target Compounds **I-1**~**I-17**

Intermediate **5a** (486 mg, 2.0 mmol) and 10 mL of triethyl orthoformate were introduced into a 50 mL round-bottom flask. The reaction mixture was stirred at 120 °C for 12 h. Upon completion of the reaction (monitored by TLC), 20 mL of water was added to the reaction mixture. The resulting solid was collected via filtration and dried to afford a crude product. The crude product was subsequently purified using column chromatography on 300~400 mesh silica gel with dichloromethane/methanol (200:1, *v*/*v*) as the eluent, yielding target compound **I-1** as a light-yellow solid (269 mg, yield 53%). Target compounds **I-2**~**I-17** were synthesized following a similar procedure.

#### 2.2.5. General Method for the Synthesis of Target Compounds **II-1**~**II-5**

The intermediate **5f** (514 mg, 2.0 mmol) was introduced into a 50 mL round-bottom flask containing 10 mL of 5% hydrochloric acid. The mixture was subsequently cooled to 0 °C. Thereafter, 1 mL of a 3.0 M sodium nitrite aqueous solution was gradually added under controlled conditions. The reaction mixture was then stirred at 100 °C for 12 h. Upon completion of the reaction (monitored by TLC), the reaction solution was extracted with dichloromethane (20 mL × 3). The organic phase was separated, concentrated under reduced pressure, and the residue was purified via column chromatography on 100~200 mesh silica gel using a gradient eluent of petroleum ether/ethyl acetate (30:1 to 20:1, *v*/*v*). This process yielded the target compound **II-1** as a light-yellow solid (210 mg, yield 39%). Target compounds **II-2**~**II-5** were synthesized following a similar procedure.

In the detailed physiochemical information, the yields ^1^H NMR, ^13^C NMR, and HRMS of all target compounds are as follows:

5-Phenyl-4,5-dihydrotetrazolo [1,5-a]thieno [2,3-e]pyridine (**I-1**): Yellow solid; Yield, 53%; M.p. 101–102 °C; ^1^H NMR (500 MHz, CDCl_3_) δ 7.57 (d, J = 5.4 Hz, 1H), 7.42–7.34 (m, 4H), 7.26–7.22 (m, 2H), 4.60 (dd, J = 10.1, 7.1 Hz, 1H), 3.78 (dd, J = 16.7, 7.1 Hz, 1H), 3.53 (dd, J = 16.7, 10.1 Hz, 1H); ^13^C NMR (125 MHz, CDCl_3_) δ 149.99, 140.27, 130.72, 130.57, 129.34, 128.42, 127.39, 126.84, 117.57, 39.82, 29.16; HRMS, *m*/*z* calcd. for C_13_H_11_N_4_S^+^ [M + H]^+^ 255.06989, found 255.07018.

5-(2-Chlorophenyl)-4,5-dihydrotetrazolo [1,5-a]thieno [2,3-e]pyridine (**I-2**): Yellow solid; Yield, 68%; M.p. 159–160 °C; ^1^H NMR (500 MHz, CDCl_3_) δ 7.61 (d, J = 5.4 Hz, 1H), 7.45 (d, J = 5.6 Hz, 2H), 7.30–7.23 (m, 1H), 7.17 (td, J = 7.6, 0.9 Hz, 1H), 6.89 (dd, J = 7.8, 1.3 Hz, 1H), 5.18 (t, J = 7.4 Hz, 1H), 3.77 (dd, J = 16.9, 7.6 Hz, 1H), 3.63 (dd, J = 16.9, 7.3 Hz, 1H); ^13^C NMR (125 MHz, CDCl_3_) δ 149.56, 137.65, 133.07, 131.35, 130.34, 129.56, 128.61, 128.19, 127.77, 127.21, 117.54, 35.92, 27.52. HRMS, *m*/*z* calcd. for C_13_H_10_ClN_4_S^+^ [M + H]^+^ 289.03092, found 289.03110.

5-(3-Chlorophenyl)-4,5-dihydrotetrazolo [1,5-a]thieno [2,3-e]pyridine (**I-3**): Yellow solid; Yield, 77%; M.p. 169–170 °C; ^1^H NMR (500 MHz, CDCl_3_) δ 7.59 (d, J = 5.4 Hz, 1H), 7.43 (dd, J = 5.4, 0.5 Hz, 1H), 7.36–7.29 (m, 2H), 7.25 (s, 1H), 7.11 (dt, J = 6.4, 1.9 Hz, 1H), 4.60 (dd, J = 9.8, 7.2 Hz, 1H), 3.79 (dd, J = 16.7, 7.1 Hz, 1H), 3.52 (dd, J = 16.7, 9.8 Hz, 1H). ^13^C NMR (125 MHz, CDCl_3_) δ 149.65, 142.28, 135.16, 130.78, 130.67, 129.54, 128.70, 127.65, 127.19, 125.58, 117.68, 39.46, 29.09. HRMS, *m*/*z* calcd. for C_13_H_10_ClN_4_S^+^ [M + H]^+^ 289.03092, found 289.03119.

5-(4-Chlorophenyl)-4,5-dihydrotetrazolo [1,5-a]thieno [2,3-e]pyridine (**I-4**): Yellow solid; Yield, 72%; M.p. 108–110 °C; ^1^H NMR (500 MHz, CDCl_3_) δ 7.58 (d, J = 5.4 Hz, 1H), 7.42 (d, J = 5.4 Hz, 1H), 7.37–7.33 (m, 2H), 7.20–7.16 (m, 2H), 4.60 (dd, J = 9.9, 7.1 Hz, 1H), 3.77 (dd, J = 16.7, 7.1 Hz, 1H), 3.49 (dd, J = 16.7, 10.0 Hz, 1H). ^13^C NMR (125 MHz, CDCl_3_) δ 149.73, 138.74, 134.34, 130.71, 129.96, 129.56, 128.78, 127.08, 117.69, 39.25, 29.15. HRMS, *m*/*z* calcd. for C_13_H_10_ClN_4_S^+^ [M + H]^+^ 289.03092, found 289.03098.

5-(2-Fluorophenyl)-4,5-dihydrotetrazolo [1,5-a]thieno [2,3-e]pyridine (**I-5**): Yellow solid; Yield, 62%; M.p. 122–123 °C; ^1^H NMR (500 MHz, CDCl_3_) δ 7.59 (d, J = 5.4 Hz, 1H), 7.43 (dd, J = 5.4, 0.5 Hz, 1H), 7.35–7.28 (m, 1H), 7.18–7.04 (m, 2H), 6.95 (td, J = 7.6, 1.6 Hz, 1H), 4.97 (t, J = 7.7 Hz, 1H), 3.76 (dd, J = 16.8, 7.4 Hz, 1H), 3.64 (dd, J = 16.8, 8.0 Hz, 1H). ^13^C NMR (125 MHz, CDCl_3_) δ 160.11 (d, J = 247.3 Hz), 149.69, 131.00, 130.13 (d, J = 8.4 Hz), 128.71, 128.35 (d, J = 3.5 Hz), 127.25 (d, J = 13.2 Hz), 126.95, 124.93 (d, J = 3.6 Hz), 117.60, 116.18 (d, J = 21.5 Hz), 32.87 (d, J = 3.1 Hz), 27.61. HRMS, *m*/*z* calcd. for C_13_H_10_FN_4_S^+^ [M + H]^+^ 273.06047, found 273.06070.

5-(3-Fluorophenyl)-4,5-dihydrotetrazolo [1,5-a]thieno [2,3-e]pyridine (**I-6**): Yellow solid; Yield, 61%; M.p. 124–125 °C; ^1^H NMR (500 MHz, CDCl_3_) δ 7.59 (d, J = 5.4 Hz, 1H), 7.43 (d, J = 5.4 Hz, 1H), 7.35 (td, J = 8.0, 6.0 Hz, 1H), 7.04 (ddd, J = 14.0, 7.9, 4.7 Hz, 2H), 6.97–6.91 (m, 1H), 4.63 (dd, J = 9.5, 7.2 Hz, 1H), 3.79 (dd, J = 16.7, 7.1 Hz, 1H), 3.53 (dd, J = 16.7, 9.7 Hz, 1H). ^13^C NMR (125 MHz, CDCl_3_) δ 163.11 (d, J = 248.0 Hz), 149.67, 142.68 (d, J = 6.7 Hz), 131.02 (d, J = 8.3 Hz), 130.76, 129.61, 127.14, 123.07 (d, J = 2.8 Hz), 117.66, 115.48 (d, J = 21.1 Hz), 114.50 (d, J = 22.1 Hz), 39.45, 29.08. HRMS, *m*/*z* calcd. for C_13_H_10_FN_4_S^+^ [M + H]^+^ 273.06047, found 273.06049.

5-(4-Fluorophenyl)-4,5-dihydrotetrazolo [1,5-a]thieno [2,3-e]pyridine (**I-7**): Yellow solid; Yield, 55%; M.p. 123–124 °C; ^1^H NMR (500 MHz, CDCl_3_) δ 7.50 (d, J = 5.4 Hz, 1H), 7.34 (d, J = 5.4 Hz, 1H), 7.15 (dd, J = 8.5, 5.2 Hz, 2H), 6.99 (t, J = 8.5 Hz, 2H), 4.54 (dd, J = 10.1, 7.1 Hz, 1H), 3.70 (dd, J = 16.7, 7.0 Hz, 1H), 3.42 (dd, J = 16.7, 10.1 Hz, 1H). ^13^C NMR (125 MHz, CDCl_3_) δ 161.48 (d, J = 248.0 Hz), 148.79, 135.00 (d, J = 3.1 Hz), 129.48 (d, J = 23.8 Hz), 128.07 (d, J = 8.3 Hz), 125.94, 116.63, 115.28 (d, J = 21.9 Hz), 38.11, 28.27. HRMS, *m*/*z* calcd. for C_13_H_10_FN_4_S^+^ [M + H]^+^ 273.06047, found 273.06067.

5-(3-(Trifluoromethyl)phenyl)-4,5-dihydrotetrazolo [1,5-a]thieno [2,3-e]pyridine (**I-8**): Yellow solid; Yield, 53%; M.p. 141–142 °C; ^1^H NMR (500 MHz, CDCl_3_) δ 7.63 (d, J = 7.8 Hz, 1H), 7.60 (d, J = 5.4 Hz, 1H), 7.57 (s, 1H), 7.52 (t, J = 7.8 Hz, 1H), 7.44 (dd, J = 5.4, 0.6 Hz, 1H), 7.40 (d, J = 7.8 Hz, 1H), 4.70 (dd, J = 10.0, 7.1 Hz, 1H), 3.83 (dd, J = 16.7, 7.1 Hz, 1H), 3.54 (dd, J = 16.7, 10.1 Hz, 1H). ^13^C NMR (125 MHz, CDCl_3_) δ 149.59, 141.32, 131.75 (dd, J = 65.3, 32.7 Hz), 130.94, 130.79, 130.01, 129.28, 127.30, 125.42 (q, J = 3.5 Hz), 124.37 (q, J = 3.7 Hz), 123.71 (q, J = 272.3 Hz), 117.77, 39.68, 29.20. HRMS, *m*/*z* calcd. for C_14_H_10_F_3_N_4_S^+^ [M + H]^+^ 323.05728, found 323.05713.

5-(4-(Trifluoromethyl)phenyl)-4,5-dihydrotetrazolo [1,5-a]thieno [2,3-e]pyridine (**I-9**): Yellow solid; Yield, 48%; M.p. 155–156 °C; ^1^H NMR (500 MHz, CDCl_3_) δ 7.64 (d, J = 8.2 Hz, 2H), 7.60 (d, J = 5.4 Hz, 1H), 7.45 (d, J = 5.4 Hz, 1H), 7.37 (d, J = 8.2 Hz, 2H), 4.71 (dd, J = 9.4, 7.3 Hz, 1H), 3.81 (dd, J = 16.7, 7.2 Hz, 1H), 3.55 (dd, J = 16.7, 9.5 Hz, 1H). ^13^C NMR (125 MHz, CDCl_3_) δ 149.53, 144.26, 131.14, 130.91, 130.75 (q, J = 32.9 Hz), 129.18, 127.87, 127.28, 126.38 (q, J = 3.6 Hz), 123.78 (q, J = 272.5 Hz), 117.73, 39.55, 29.05. HRMS, *m*/*z* calcd. for C_14_H_10_F_3_N_4_S^+^ [M + H]^+^ 323.05728, found 323.05743.

5-(3-Methoxyphenyl)-4,5-dihydrotetrazolo [1,5-a]thieno [2,3-e]pyridine (**I-10**): Yellow solid; Yield, 66%; M.p. 104–105 °C; ^1^H NMR (500 MHz, CDCl_3_) δ 7.57 (d, J = 5.4 Hz, 1H), 7.39 (d, J = 5.4 Hz, 1H), 7.30 (t, J = 8.0 Hz, 1H), 6.88 (dd, J = 8.2, 2.3 Hz, 1H), 6.82 (d, J = 7.7 Hz, 1H), 6.79–6.74 (m, 1H), 4.57 (dd, J = 10.1, 7.1 Hz, 1H), 3.89–3.68 (m, 4H), 3.53 (dd, J = 16.7, 10.2 Hz, 1H). ^13^C NMR (125 MHz, CDCl_3_) δ 160.18, 149.97, 141.77, 130.43, 126.86, 119.57, 117.56, 113.48, 113.35, 55.31, 39.85, 29.12. HRMS, *m*/*z* calcd. for C_14_H_13_N_4_OS^+^ [M + H]^+^ 285.08046, found 285.08057.

5-(4-Methoxyphenyl)-4,5-dihydrotetrazolo [1,5-a]thieno [2,3-e]pyridine (**I-11**): Yellow solid; Yield, 60%; M.p. 156–157 °C; ^1^H NMR (500 MHz, CDCl_3_) δ 7.56 (d, J = 5.4 Hz, 1H), 7.38 (d, J = 5.4 Hz, 1H), 7.19–7.15 (m, 2H), 6.95–6.87 (m, 2H), 4.55 (dd, J = 10.5, 7.0 Hz, 1H), 3.81 (s, 3H), 3.75 (dd, J = 16.6, 7.0 Hz, 1H), 3.48 (dd, J = 16.6, 10.5 Hz, 1H). ^13^C NMR (125 MHz, CDCl_3_) δ 159.53, 150.12, 132.17, 131.41, 130.40, 128.54, 126.69, 117.59, 114.64, 55.37, 39.15, 29.33. HRMS, *m*/*z* calcd. for C_14_H_13_N_4_OS^+^ [M + H]^+^ 285.08046, found 285.08066.

5-(4-Bromophenyl)-4,5-dihydrotetrazolo [1,5-a]thieno [2,3-e]pyridine (**I-12**): Yellow solid; Yield, 56%; M.p. 119–121 °C; ^1^H NMR (500 MHz, CDCl_3_) δ 7.58 (d, J = 5.4 Hz, 1H), 7.50 (d, J = 7.2 Hz, 2H), 7.42 (d, J = 5.3 Hz, 1H), 7.12 (d, J = 7.3 Hz, 2H), 4.70–4.45 (m, 1H), 3.77 (dd, J = 16.7, 7.0 Hz, 1H), 3.49 (dd, J = 16.6, 10.0 Hz, 1H). ^13^C NMR (125 MHz, CDCl_3_) δ 149.70, 139.27, 132.52, 130.73, 129.83, 129.09, 127.09, 122.43, 117.69, 39.33, 29.09. HRMS, *m*/*z* calcd. for C_13_H_10_BrN_4_S^+^ [M + H]^+^ 332.98041, found 332.98068.

5-(m-Tolyl)-4,5-dihydrotetrazolo [1,5-a]thieno [2,3-e]pyridine (**I-13**): Yellow solid; Yield, 42%; M.p. 142–146 °C; ^1^H NMR (500 MHz, CDCl_3_) δ 7.57 (d, J = 5.4 Hz, 1H), 7.39 (d, J = 5.4 Hz, 1H), 7.26 (t, J = 7.6 Hz, 1H), 7.15 (d, J = 7.6 Hz, 1H), 7.07–7.00 (m, 2H), 4.55 (dd, J = 10.2, 7.1 Hz, 1H), 3.76 (dd, J = 16.7, 7.1 Hz, 1H), 3.52 (dd, J = 16.7, 10.3 Hz, 1H), 2.35 (s, 3H). ^13^C NMR (125 MHz, CDCl_3_) δ 150.1), 140.3, 139.1, 130.9, 130.5, 129.2, 129.2, 128.1, 126.8, 124.4, 117.5, 39.8, 29.2, 21.5. HRMS, *m*/*z* calcd. for C_14_H_13_N_4_S^+^ [M + H]^+^ 269.08554, found 269.08563.

5-(p-Tolyl)-4,5-dihydrotetrazolo [1,5-a]thieno [2,3-e]pyridine (**I-14**): Yellow solid, yield 48%, M.p. 103–105 °C; ^1^H NMR (500 MHz, CDCl_3_) δ 7.56 (d, J = 5.4 Hz, 1H), 7.38 (d, J = 5.4 Hz, 1H), 7.18 (d, J = 8.0 Hz, 2H), 7.13 (d, J = 8.1 Hz, 2H), 4.56 (dd, J = 10.3, 7.1 Hz, 1H), 3.75 (dd, J = 16.7, 7.0 Hz, 1H), 3.50 (dd, J = 16.7, 10.4 Hz, 1H), 2.36 (s, 3H). ^13^C NMR (125 MHz, CDCl_3_) δ 150.10, 138.26, 137.25, 131.12, 130.47, 129.98, 127.27, 126.71, 117.55, 39.50, 29.21, 21.13. HRMS, *m*/*z* calcd. for C_14_H_13_N_4_S^+^ [M + H]^+^ 269.08554, found 269.08572.

5-(Thiophen-2-yl)-4,5-dihydrotetrazolo [1,5-a]thieno [2,3-e]pyridine (**I-15**): Yellow solid, yield 43%, M.p. 120–122 °C; ^1^H NMR (500 MHz, CDCl_3_) δ 7.56 (d, J = 5.4 Hz, 1H), 7.42 (d, J = 5.4 Hz, 1H), 7.30–7.24 (m, 1H), 6.97 (dd, J = 5.1, 3.6 Hz, 1H), 6.92 (d, J = 3.4 Hz, 1H), 4.93 (dd, J = 8.9, 6.9 Hz, 1H), 3.82 (dd, J = 16.6, 6.8 Hz, 1H), 3.65 (dd, J = 16.6, 9.1 Hz, 1H). ^13^C NMR (125 MHz, CDCl_3_) δ 149.69, 142.81, 130.21, 130.18, 127.24, 126.96, 125.72, 125.53, 117.66, 34.92, 29.81. HRMS, *m*/*z* calcd. for C_11_H_9_N_4_S_2_^+^ [M + H]^+^ 261.02631, found 261.02658.

5-([1,1′-Biphenyl]-4-yl)-4,5-dihydrotetrazolo [1,5-a]thieno [2,3-e]pyridine (**I-16**): Yellow solid, yield 30%, M.p. 161–164 °C; ^1^H NMR (500 MHz, CDCl_3_) δ 7.58 (dd, J = 8.5, 6.7 Hz, 5H), 7.45 (t, J = 7.6 Hz, 2H), 7.41 (d, J = 5.4 Hz, 1H), 7.37 (d, J = 7.3 Hz, 1H), 7.30 (d, J = 8.2 Hz, 2H), 4.65 (dd, J = 9.9, 7.1 Hz, 1H), 3.81 (dd, J = 16.7, 7.1 Hz, 1H), 3.57 (dd, J = 16.7, 10.0 Hz, 1H). ^13^C NMR (125 MHz, CDCl_3_) δ 149.98, 141.39, 140.22, 139.19, 130.64, 130.60, 128.90, 128.01, 127.81, 127.67, 127.08, 126.88, 117.61, 39.52, 29.20. HRMS, *m*/*z* calcd. for C_19_H_15_N_4_S^+^ [M + H]^+^ 331.10119, found 331.10141.

5-Cyclopropyl-4,5-dihydrotetrazolo [1,5-a]thieno [2,3-e]pyridine (**I-17**): Yellow solid, yield 39%, M.p. 68–72 °C; ^1^H NMR (500 MHz, CDCl_3_) δ 7.52 (d, J = 5.4 Hz, 1H), 7.37 (d, J = 5.4 Hz, 1H), 3.64 (dd, J = 16.6, 6.8 Hz, 1H), 3.27 (dd, J = 16.6, 10.7 Hz, 1H), 2.54 (td, J = 10.1, 6.9 Hz, 1H), 1.12–1.00 (m, 1H), 0.82–0.65 (m, 2H), 0.46–0.36 (m, 2H). ^13^C NMR (125 MHz, CDCl_3_) δ 150.44, 131.36, 129.56, 126.00, 117.63, 39.50, 26.78, 16.30, 4.86, 4.02. HRMS, *m*/*z* calcd. for C_10_H_11_N_4_S^+^ [M + H]^+^ 219.06989, found 219.07007.

5-(p-Tolyl)-4,5-dihydrothieno [2,3-e][1,2,4]triazolo [4,3-a]pyridine (**II-1**): Yellow solid, yield 37%, M.p. 86–88 °C; ^1^H NMR (500 MHz, CDCl_3_) δ 8.48 (s, 1H), 7.32 (d, J = 5.4 Hz, 1H), 7.26 (s, 1H), 7.18 (s, 1H), 7.16 (d, J = 1.2 Hz, 2H), 7.15 (s, 1H), 4.45 (dd, J = 10.2, 6.5 Hz, 1H), 3.62 (dd, J = 16.1, 6.5 Hz, 1H), 3.38 (dd, J = 16.1, 10.3 Hz, 1H), 2.35 (s, 3H). ^13^C NMR (125 MHz, CDCl_3_) δ 149.62, 137.89, 137.85, 137.49, 130.14, 129.81, 127.35, 127.04, 126.10, 116.62, 39.65, 30.06, 21.12. HRMS, *m*/*z* calcd. for C_15_H_14_N_3_S^+^ [M + H]^+^ 268.09029, found 268.09039.

5-(Thiophen-2-yl)-4,5-dihydrothieno [2,3-e][1,2,4]triazolo [4,3-a]pyridine (**II-2**): Yellow solid, yield 31%, M.p. 196–199 °C; ^1^H NMR (500 MHz, CDCl_3_) δ 8.61 (s, 1H), 7.17 (d, J = 5.0 Hz, 1H), 7.09 (d, J = 5.3 Hz, 1H), 6.98–6.86 (m, 2H), 6.65 (d, J = 5.2 Hz, 1H), 4.60 (t, J = 6.6 Hz, 1H), 3.17 (dt, J = 15.0, 5.4 Hz, 1H), 3.05 (dd, J = 15.1, 7.5 Hz, 1H). ^13^C NMR (125 MHz, CDCl_3_) δ 149.74, 145.12, 134.91, 125.83, 123.59, 123.55, 123.29, 123.00, 116.83, 36.37, 33.36, 33.29. HRMS, *m*/*z* calcd. for C_12_H_10_N_3_S_2_^+^ [M + H]^+^ 260.03107, found 260.03116.

5-([1,1′-Biphenyl]-4-yl)-4,5-dihydrothieno [2,3-e][1,2,4]triazolo [4,3-a]pyridine (**II-3**): Yellow solid, yield 43%, M.p. 129–132 °C; ^1^H NMR (500 MHz, CDCl_3_) δ 8.52 (s, 1H), 7.58–7.44 (m, 4H), 7.36 (t, J = 6.0 Hz, 2H), 7.31 (d, J = 8.1 Hz, 2H), 7.20 (d, J = 5.3 Hz, 2H), 4.54 (dd, J = 9.6, 6.6 Hz, 1H), 3.67 (dd, J = 16.1, 6.5 Hz, 1H), 3.46 (dd, J = 16.1, 9.8 Hz, 1H). ^13^C NMR (125 MHz, CDCl_3_) δ 141.07, 140.38, 139.82, 137.55, 130.28, 129.6, 128.86, 127.87, 127.86, 127.55, 127.09, 126.29, 116.68, 39.63, 30.02. HRMS, *m*/*z* calcd. for C_20_H_16_N_3_S^+^ [M + H]^+^ 330.10594, found 330.10608.

5-(4-Fluorophenyl)-4,5-dihydrothieno [2,3-e][1,2,4]triazolo [4,3-a]pyridine (**II-4**): Yellow solid, yield 34%, M.p. 110–113 °C; ^1^H NMR (500 MHz, CDCl_3_) 10.45–10.21 (m, 0.5H), 8.51–8.47 (m, 0.5H), 7.43–7.34 (m, 2H), 7.31 (dd, J = 10.4, 5.5 Hz, 0.5H), 7.23 (dd, J = 8.6, 5.2 Hz, 0.5H), 7.17–6.99 (m, 3H), 5.35–5.29 (m, 0.5H), 4.50–4.29 (m, 0.5H), 3.84–3.66 (m, 1H), 3.60–3.41 (m, 0.5H), 3.32–3.25 (m, 0.5H). ^13^C NMR (125 MHz, CDCl_3_) δ 163.44, 163.38, 163.00, 162.92 (d, J = 2.6 Hz), 161.48 (d, J = 8.5 Hz), 160.90, 160.8, 159.70, 159.67, 151.44, 151.12, 149.25, 149.10, 139.13, 139.01, 138.87, 138.83, 136.11, 136.03, 136.01, 135.86, 133.63, 133.51, 132.02, 131.87, 129.29, 129.22, 129.14, 129.07, 128.81, 128.79, 128.75, 125.99, 125.95, 124.56, 124.42, 123.26, 123.17, 117.08, 116.25, 116.07, 115.92, 115.87, 115.75, 115.70, 39.36, 39.33, 38.95, 38.60, 35.20, 35.10, 30.30, 29.72. HRMS, *m*/*z* calcd. for C_14_H_11_FN_3_S^+^ [M + H]^+^ 272.06522, found 272.06531.

5-Cyclopropyl-4,5-dihydrothieno [2,3-e][1,2,4]triazolo [4,3-a]pyridine (**II-5**): Yellow solid, yield 41%, M.p. 171–173 °C; ^1^H NMR (500 MHz, CDCl_3_) δ 10.45 (d, J = 12.5 Hz, 0.5H), 8.44 (s, 0.5H), 7.43–7.34 (m, 1H), 7.29–7.25 (m, 0.5H), 7.11 (dd, J = 19.0, 5.3 Hz, 0.5H), 3.63–3.46 (m, 0.5H), 3.45–3.23 (m, 1H), 3.16–3.06 (m, 0.5H), 3.04–2.94 (m, 0.5H), 2.45–2.23 (m, 0.5H), 1.36–1.23 (m, 1H), 1.05–0.91 (m, 0.5H), 0.81–0.56 (m, 2H), 0.45–0.26 (m, 2H). ^13^C NMR (125 MHz, CDCl_3_) δ 159.62, 159.59, 151.80, 151.58, 149.65, 149.46, 133.69, 133.63, 132.52, 132.26, 131.99, 129.20, 125.09, 125.05, 124.29, 124.19, 121.98, 121.92, 117.29, 117.18, 40.27, 40.00, 39.66, 39.63, 36.05, 36.00, 28.27, 28.14, 19.74, 19.66, 16.03, 15.83, 6.11, 6.01, 5.63, 5.60, 4.82, 4.79, 3.99, 3.87. HRMS, *m*/*z* calcd. for C_11_H_12_N_3_S^+^ [M + H]^+^ 218.07464, found 218.07468.

### 2.3. Fungicidal Activity

In vitro fungicidal activities of target compounds **I-1**~**I-l7** and **II-1**~**II5** against *Alternaria solani*, *Botrytis cinerea*, *Cercospora arachidicola*, *Fusarium graminearum*, *Physalospora piricola*, *Rhizoctonia solani*, and *Sclerotinia sclerotiorum* were evaluated according to our previously reported method [9]. The commercial fungicides flutriafol and thifluzamide were used for comparison. For the preparation of the mother solution, 5 mg of the tested compound was precisely weighed and dissolved in 200 μL of N, N-dimethylformamide (DMF) to obtain a concentration of 25,000 μg/mL. To prepare the working solution with a test concentration of 500 μg/mL, 100 μL of the mother solution was carefully added to 4900 μL of sterile water containing 0.1% Tween 80 and thoroughly mixed. Finally, 50 μg/mL of potato glucose agar (PDA) was formulated by uniformly mixing 4 mL of the working solution with 36 mL of PDA. The control was prepared by pure DMF and PDA medium according to the corresponding amount described above. Each experiment was repeated three times. The relative inhibition (I, %) was calculated using the equation below:

I (%) = [(C − T)/(C − 4)] × 100%

where I represent the rate of fungal growth inhibition, C denotes the average diameter of the control colony (mm), and T indicates the average diameter of the mycelial colony in the experiment (mm). The diameter of the mycelial cake inoculated on PDA was 4 mm.

### 2.4. Transcriptome Analyses

RNA extraction was conducted according to the previously reported method [36,37,38]. A mother solution of target compound **I-12** (25,000 μg/mL) was prepared with dimethyl sulfoxide as a solvent, and then 100 μL of the mother solution was mixed with 4900 μL of sterile water with 0.1% Tween 80 to prepare the tested solution (500 μg/mL). The culture with a concentration of 6.66 μg/mL of target compound **I-12** was prepared by adding 1 mL of the test solution to 99 mL of PDB medium. The control sample including all the components without **I-12** was set up. Subsequently, *R. solani* was cultivated in PDB medium for three days. Following this, the *R. solani* mycelia were harvested for RNA extraction. Each treatment consisted of three replicates.

RNA samples were sent to Majorbio (Shanghai, China) for transcriptome study. *R. solani* AG-1 IA genome (assembly accession: GCA_000334115) was used as a reference for mapping the short reads. The data were analyzed on the online platform of Majorbio Cloud Platform (www.majorbio.com, accessed on 21 February 2025). The genes with *p* < 0.05 and |Log 2 Fold Change| ≥ 1 across three biological replicates were identified as up- or down-regulated differentially expressed genes (DEGs). The functional annotations of the DEGs were performed using Gene Ontology (GO) (http://www.geneontology.org, accessed on 22 February 2025), and the biological and functional properties of the DEGs were identified using the Kyoto Encyclopaedia of Genes and Genomes (KEGG) database (http://www.genome.jp/kegg/, accessed on 22 February 2025).

### 2.5. Statistical Analysis

The values presented in each table represent the mean ± standard deviation (SD) derived from at least three repeated experiments. Statistical analysis was performed using the DPS 7.05 data processing system (DPS, Hangzhou, China).

## 3. Results and Discussion

### 3.1. Chemistry

Target compounds **I-1**~**I-17** and **II-1**~**II-5** were synthesized via a five-step synthetic route as depicted in Scheme 1. The reaction sequence was initiated by a base-promoted ester hydrolysis of methyl 3-aminothiophene-2-carboxylate. In brief, methyl 3-aminothiophene-2-carboxylate reacted with sodium hydroxide at 90 °C in ethanol to yield intermediate **2**, which subsequently underwent a cyclization reaction with Meldrum’s acid and the corresponding aldehyde to produce intermediates **3a**~**3q**. These intermediates were further subjected to nucleophilic substitution reactions with phosphorus pentasulfide, resulting in the formation of thiophene derivatives **4a**~**4q**. Next, key intermediates **5a**~**5q** were obtained by treating compounds **4a**~**4q** with hydrazine hydrate in methanol under heating at 80 °C. Finally, to synthesize the target compound series **I**, intermediates **5a**~**5q** were subjected to ring-closure reactions with triethyl orthoformate, affording target compounds **I-1**~**I-17**. For the synthesis of target compounds in series **II**, intermediates **5g**, **5n**, **5o**, **5p**, and **5q** were individually treated with sodium nitrite in 5% hydrochloric acid to yield target compounds **II-1**~**II-5**. The structures of all synthesized compounds were characterized and confirmed using ^1^H NMR, ^13^C NMR, and HRMS. (Figures S7 and S72)

### 3.2. Fungicidal Activity and SAR

The in vitro antifungal activities of the target compounds **I-1**~**I-17** and **II-1**~**II-5** against *A. solani*, *B. cinerea*, *C. arachidicola*, *F. graminearum*, *P. piricola*, *R. solani*, and *S. sclero-tiorum* at a concentration of 50 μg/mL are summarized in Table 1. Commercial fungicides flutriafol and thifluzamide were used as positive controls. The results presented in Table 1 indicated that several target compounds exhibited moderate to high antifungal activity against certain tested fungal pathogens. Notably, compounds **I-6** and **I-12** demonstrate promising antifungal activity against *A. solani*, with inhibition rates exceeding 70%, surpassing thifluzamide (inhibition rate = 67%) (*p* < 0.01) but falling short of flutriafol (inhibition rate = 100%) (*p* > 0.01). Compounds **I-14** and **I-15** exhibited moderate antifungal activity against *A. solani*, with inhibition rates of 65% and 64%, respectively, comparable to thifluzamide. Additionally, compounds **I-6**, **I-7**, **I-9**, **I-12**, and **I-15** showed moderate antifungal activity against *B. cinerea*, with inhibition rates exceeding 60%, significantly outperforming thifluzamide (inhibition rate = 32%) (*p* < 0.01). Although the antifungal activities of all target compounds against *C. arachidicola* were lower compared to flutriafol and thifluzamide, several compounds still exhibited notable efficacy. For example, compounds **I-1**, **I-7**, and **I-14** displayed good antifungal activity against *C. arachidicola*, with inhibition rates of 89%, 89%, and 81%, respectively. Furthermore, compounds **I-7** and **I-11** demonstrated inhibition rates of 61% and 63% against *F. graminearum*, respectively, significantly higher than that of thifluzamide (*p* < 0.01). Moreover, some target compounds, such as **I-6**, **I-7**, and **I-12**, exhibited good antifungal activities against *R. solani*, with inhibition rates of 76%, 80%, and 79%, respectively. Additionally, four compounds (i.e., **I-1**, **I-5**, **I-7**, and **I-14**) demonstrated good antifungal activity against *S. sclerotiorum* with inhibition rates exceeding 75%, of which compounds **I-1** and **I-7** exhibited comparable antifungal activity to flutriafol (inhibition rate = 95%) with inhibition rates of 90% and 91%, respectively, which is markedly better than thifluzamide (inhibition rate = 15%) (*p* < 0.01).

Based on the antifungal results against *R. solani*, a comprehensive structure–activity relationship (SAR) was analyzed. As the results presented in Table 1, when the R group is a substituted benzene ring, the antifungal activity of the target compounds follows this order: **I-7** (R = 4-fluorophenyl, inhibition rate = 80%), **I-6** (R = 3-fluorophenyl, inhibition rate = 76%) > **I-5** (R = 2-fluorophenyl, inhibition rate = 38%); **I-4** (R = 4-chlorophenyl, inhibition rate = 65%) > **I-3** (R = 3-chlorophenyl, inhibition rate = 42%) > **I-2** (R = 2-chlorophenyl, inhibition rate = 17%); and **I-9** (R = 4-trifluoromethylphenyl, inhibition rate = 68%) > **I-8** (R = 3-trifluoromethylphenyl, inhibition rate = 51%) (*p* < 0.01). Furthermore, it was observed that substituents introduced at the four-position of the benzene ring significantly enhanced the antifungal activity of compounds **I-4**, **I-7**, and **I-9** compared to compound **I-1** (R = phenyl, inhibition rate = 54%) (*p* < 0.01). In contrast, compounds **I-11** (R = 4-methoxyphenyl, inhibition rate = 37%) and **I-14** (R = 4-methylphenyl, inhibition rate = 41%) exhibited lower antifungal activity than compound **I-1** (*p* > 0.01). These findings suggest that the position of the substituent on the benzene ring has a significant impact on antifungal activity, with electron-withdrawing substituents at the four-position enhancing activity. Additionally, it was found that compounds with an F atom at the four-position of the benzene ring (i.e., compound **I-7**) demonstrated superior fungicidal activity compared to their corresponding Cl-substituted analogs (i.e., compound **I-4**) and CF_3_-substituted analogs (i.e., compound **I-9**) (*p* < 0.01), suggesting that the introduction of an F atom is more beneficial for improving antifungal activity. When the R group is a thiophene ring, compound **I-15** (inhibition rate = 62%) demonstrates higher antifungal activity compared to compound **I-1** (*p* < 0.01). In contrast, when the R group is a cycloalkane, compound **I-17** (inhibition rate = 22%) exhibits lower antifungal activity than compound **I-1** (*p* > 0.01). These results suggest that the introduction of a heteroaromatic ring enhances antifungal efficacy.

**Figure 2:**
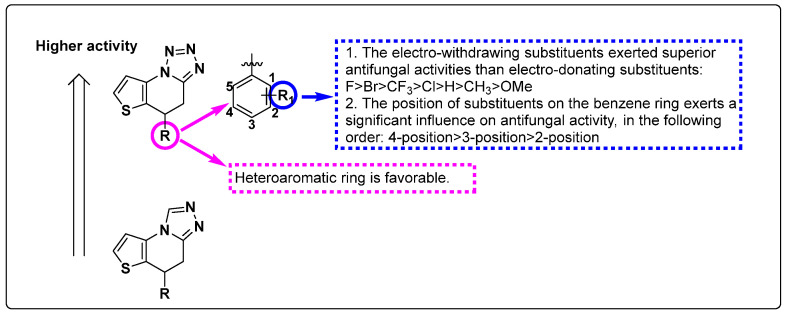
The summarized SAR analysis.

Furthermore, a comparative analysis of the antifungal activities against *R. solani* between target compound series **I** and **II**, which share an identical R group, revealed that series compound **I** generally exhibited superior antifungal efficacy compared to its corresponding series compound **II**. For instance, compounds **I-7**, **I-14**, **I-15**, **I-16**, and **I-17** demonstrated antifungal efficacies of 80%, 41%, 62%, 59%, and 22% against *R. solani*, respectively, whereas their corresponding compounds **II-4**, **II-1**, **II-2**, **II-3**, and **II-5** showed inhibition rates of 32%, 26%, 31%, 37%, and 10% against *R. solani*, respectively. These results indicate that compounds with a 4,5-dihydrotetrazolo [1,5-a]thieno [2,3-e]pyridine scaffold possess enhanced antifungal activity relative to those with a 4,5-dihydrothieno [2,3-e] [1,2,4]triazolo [4,3-a]pyridine scaffold. Overall, these SAR analysis results provide valuable insights for guiding the design of compounds with improved antifungal activity. The summarized SAR analysis was shown in Figure 2.

To further investigate the antifungal potential of the target compound, compounds with antifungal inhibition rate exceeding 70% were selected for further evaluation of their median effective concentration (EC_50_) values. As the results show in Table 2, compound **I-12** exhibited an EC_50_ value of 9.49 μg/mL against *A. solani*, indicating its significant antifungal efficacy. Compounds **I-5**, **I-7**, and **I-14** showed substantial antifungal activities against *C. arachidicola*, with EC_50_ values of 8.83 μg/mL, 6.46 μg/mL, and 5.15 μg/mL, respectively. Among the tested compounds, **I-12** was the most effective against *R. solani*, with an EC_50_ value of 6.66 μg/mL. Additionally, compounds **I-1**, **I-7**, and **I-14** displayed relatively strong antifungal activities against *S. sclerotiorum*, with EC_50_ values of 4.61 μg/mL, 5.89 μg/mL, and 7.18 μg/mL, respectively. While the antifungal efficacy of these compounds is less potent compared to that of commercial fungicides flutriafol and thifluzamide, the encouraging results indicate that compounds **I-1**, **I-5**, **I-7**, **I-12**, and **I-14** exhibit favorable antifungal properties. These promising findings suggest that 4,5-dihydrotetrazolo [1,5-a]thieno [2,3-e]pyridine merit further exploration as potential lead scaffold for fungicide development.

**Figure 3:**
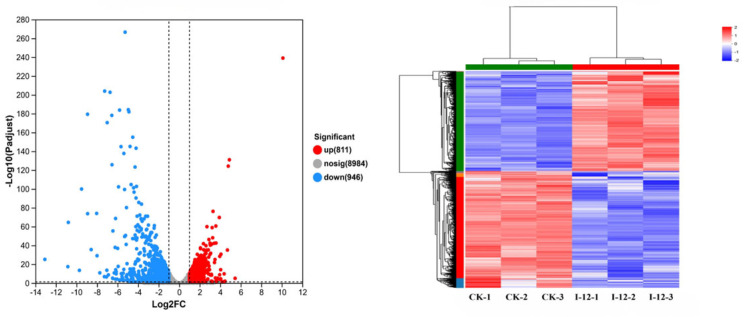
Transcriptome analysis of *R. solani* treated by **I-12**. (**Left**) Volcano plots. (**Right**) Heatmap.

**Figure 4:**
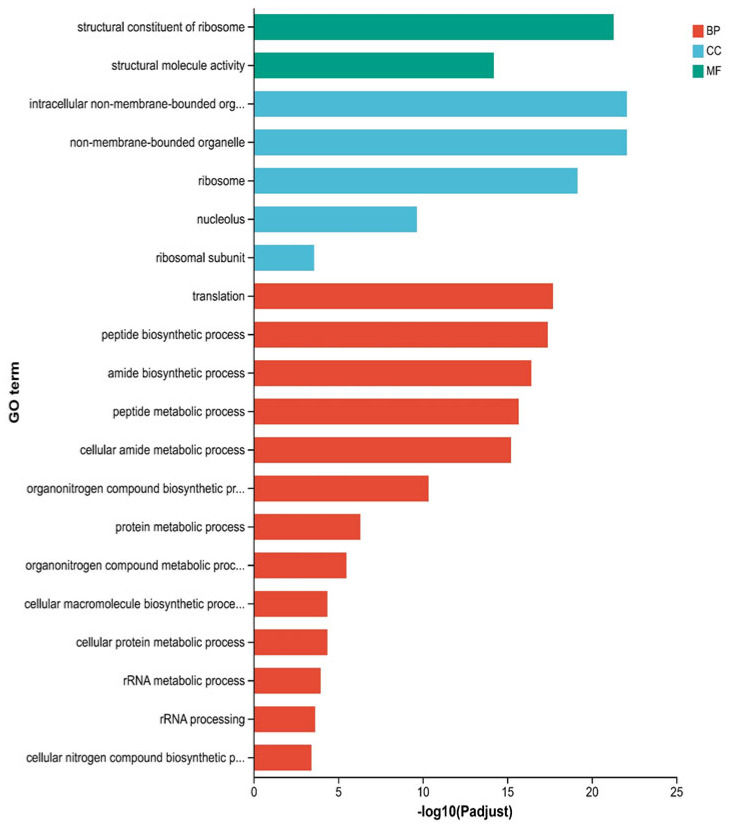
GO analysis of DEGs.

To preliminarily investigate the antifungal mechanism of target compounds, compound **I-12** was selected as a representative to explore its molecular mode of action at the gene expression level using transcriptome sequencing technology. Based on the quantitative analysis of *R. solani* expression levels following treatment with **I-12**, an inter-group differential gene expression analysis was conducted. The volcano plots and heatmap for these DEGs are presented in Figure 3. A total of 1757 differentially expressed genes (DEGs) were identified, of which 811 were significantly up-regulated and 946 were significantly down-regulated. Subsequently, these DEGs were used for Gene Ontology (GO) and Kyoto Encyclopedia of Genes and Genomes (KEGG) annotations. The GO enrichment histogram provides a comprehensive overview of the enrichment and distribution of DEGs across three categories: molecular function (MF), cellular component (CC), and biological process (BP). Figure 4 illustrates the top 20 GO functional categories that show significant enrichment (*p*-adjust < 0.05) along with their annotations. Among them, 13 GO terms were related to biological processes, mainly including translation, peptide biosynthetic process, amide biosynthetic process, peptide metabolic process, cellular amide metabolic process, organonitrogen compound biosynthesis process, protein metabolic process, organonitrogen compound metabolic process, cellular macromolecule biosynthetic process, cellular protein metabolic process, rRNA metabolic process, rRNA processing, and cellular nitrogen compound biosynthetic process; 5 GO terms related to cellular component, mainly including intracellular non-membrane-bounded organelle, non-membrane-bounded organelle, ribosome, nucleolus, and ribosomal subunit; 2 GO terms related to structural constituent of ribosome and structural molecule activity. The GO enrichment analysis demonstrated that compound **I-12** treatment significantly affected the metabolic process and cellular process of *R. solani*.

**Figure 5:**
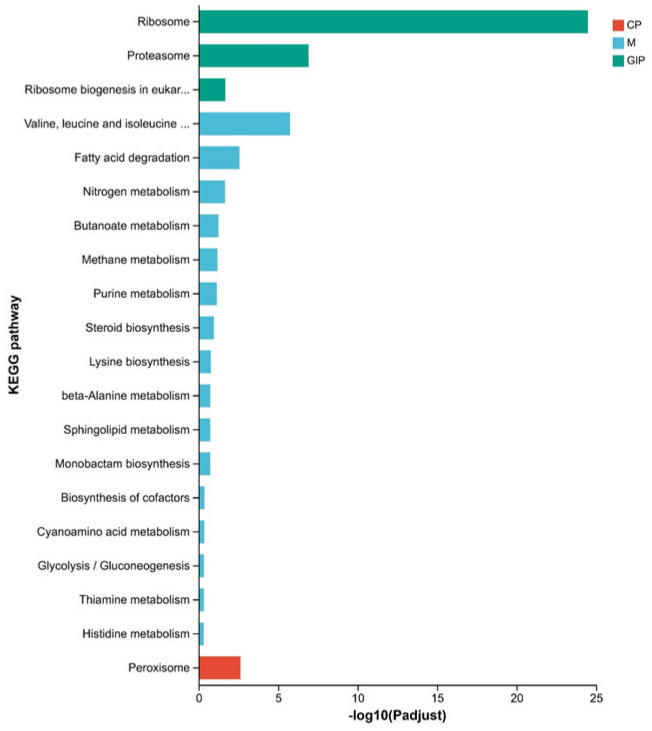
KEGG pathway analysis of DEGs.

Furthermore, the primary metabolic pathways associated with DEGs were identified via KEGG pathway enrichment analysis. Figure 5 provides the top 20 enriched metabolic pathways, of which the most enrichment pathway is ribosome (Figure S73). In addition, it was found that DEGs are also significantly enriched at the proteasome (Figure S79) and ribosome biogenesis in eukaryotes pathway (Figure S77). The ribosome and proteasome pathways are associated with genetic information processing, particularly translation-related processes. Additionally, the pathways related to valine–leucine–isoleucine degradation (Figure S74), fatty acid degradation (Figure S78), and nitrogen metabolism (Figure S75) fall under metabolic processes. The pathway related to peroxisome (Figure S76) fall under cell processes. Notably, DEGs involved in nitrogen metabolism and proteasome pathways exhibited significant down-regulation. Nitrogen metabolism plays a critical role in the growth, development, reproduction, and maintenance of normal physiological functions in fungal organisms. The inhibition of the nitrogen metabolism pathway can disrupt essential biochemical processes such as amino acid and protein synthesis. Similarly, suppression of the proteasome pathway can lead to imbalances in protein metabolism, reducing stress resistance and impairing intracellular signal transduction, thereby affecting fundamental life activities like growth and reproduction in fungi. These findings indicate that compound **I-12** exhibits antifungal activity by modulating nitrogen metabolism and proteasome-related biological pathways in *R. solani*.

## 4. Conclusions

By extending the scope of bioactivity screening within our laboratory’s molecular library, we successfully discovery a series of 4,5,6,7-tetrahydrothieno [3,2-b] pyridine derivatives that exhibit promising antifungal efficacy. Specifically, compounds **I-1**, **I-5**, **I-7**, and **I-12** demonstrated potent antifungal activity against *Candida arachidicola*, *Rhizoctonia solani*, and *Sclerotinia sclerotiorum*, with EC_50_ values ranging from 4.61 to 6.66 μg/mL. SAR studies revealed that the R group significantly influences antifungal activity, and compounds featuring a 4,5-dihydrotetrazolo [1,5-a]thieno [2,3-e]pyridine scaffold exhibited enhanced antifungal potency compared to those with a 4,5-dihydrothieno [2,3-e] [1,2,4]triazolo [4,3-a]pyridine scaffold. Furthermore, transcriptome analyses were conducted to preliminarily investigate the molecular mode of action for compound **I-12**, which suggests its potential to inhibit nitrogen metabolism and the proteasome pathway, thereby disrupting normal fungal growth and development and exerting fungicidal effects. In summary, this study has identified a novel class of antifungal compounds with a unique structural framework, warranting further in-depth investigation. This research is expected to provide critical theoretical foundations and innovative insights for the development of novel and highly efficient fungicides. Ongoing efforts in our laboratory are focused on structural optimization and elucidation of the molecular mechanism of action of the 4,5,6,7-tetrahydrothieno [3,2-b] pyridine scaffold.

## Figures and Tables

**Table 1:** In vitro antifungal activity of the target compounds **I-1** to **I-17** and **II-1** to **II-5** at a concentration of 50 μg/mL (% inhibition) ^1^.

Comp.	*A. s* ^2^	*B. c* ^2^	*C. a* ^2^	*F. g* ^2^	*P. p* ^2^	*R. s* ^2^	*S. s* ^2^
**I-1**	56 ± 1e	34 ± 1h	89 ± 1b	43 ± 1f	62 ± 1c	54 ± 1g	90 ± 1b
**I-2**	44 ± 1g	58 ± 1d	27 ± 1k	45 ± 1d	42 ± 1f	17 ± 4l	46 ± 1h
**I-3**	47 ± 4g	39 ± 1g	13 ± 1n	17 ± 1k	55 ± 0d	42 ± 2i	10 ± 1o
**I-4**	52 ± 1f	24 ± 1j	30 ± 1j	15 ± 1k	28 ± 1h	65 ± 1d	45 ± 1i
**I-5**	58 ± 2e	24 ± 1j	75 ± 1d	24 ± 1j	29 ± 1h	38 ± 1i	78 ± 1d
**I-6**	76 ± 1c	64 ± 0c	58 ± 1g	44 ± 1e	51 ± 0e	76 ± 1c	49 ± 1g
**I-7**	59 ± 4e	61 ± 0c	89 ± 1b	61 ± 0b	26 ± 0i	80 ± 1c	91 ± 1b
**I-8**	35 ± 1i	47 ± 2f	39 ± 1i	46 ± 0d	39 ± 1g	51 ± 1h	58 ± 1f
**I-9**	57 ± 1e	61 ± 1c	20 ± 1m	45 ± 0e	30 ± 0h	68 ± 0d	20 ± 0m
**I-10**	52 ± 1f	36 ± 1g	66 ± 1e	45 ± 0e	29 ± 1h	54 ± 1g	67 ± 1e
**I-11**	22 ± 1k	57 ± 1e	63 ± 1f	63 ± 1b	18 ± 1k	37 ± 1i	58 ± 1f
**I-12**	84 ± 1b	76 ± 1b	50 ± 1h	49 ± 0c	55 ± 0d	79 ± 3c	48 ± 1g
**I-13**	30 ± 1j	25 ± 1j	36 ± 1i	36 ± 1g	7 ± 1m	59 ± 3f	39 ± 1j
**I-14**	65 ± 1d	48 ± 1f	81 ± 1c	35 ± 1h	74 ± 1b	41 ± 4i	83 ± 1c
**I-15**	64 ± 1d	62 ± 0c	53 ± 1h	33 ± 2i	52 ± 2d	62 ± 1e	58 ± 0f
**I-16**	25 ± 1k	34 ± 1h	10 ± 1o	1 ± 2l	20 ± 2j	59 ± 2f	35 ± 1k
**I-17**	41 ± 1h	38 ± 5g	26 ± 1k	35 ± 1h	22 ± 1j	22 ± 0k	47 ± 2g
**II-1**	35 ± 1i	47 ± 2f	23 ± 1k	16 ± 1k	39 ± 1g	26 ± 3k	27 ± 2l
**II-2**	19 ± 4l	15 ± 4k	0q	0l	10 ± 1l	31 ± 0j	20 ± 1m
**II-3**	12 ± 1m	10 ± 1l	11 ± 2n	0l	21 ± 2j	37 ± 3i	16 ± 1n
**II-4**	24 ± 2k	55 ± 1e	22 ± 4l	47 ± 1c	29 ± 2h	32 ± 4j	40 ± 2j
**II-5**	30 ± 1j	55 ± 1e	25 ± 1k	37 ± 1g	23 ± 2j	10 ± 1m	39 ± 1j
Flutriafol	100a	97 ± 1a	97 ± 2a	100a	99 ± 1a	95 ± 1a	95 ± 1a
Thifluzamide	67 ± 2d	32 ± 2i	100a	44 ± 1e	22 ± 2j	90 ± 1b	15 ± 1n

^1^ Each value represents the mean ± SD of three experiments. Different letters represent significant differences between different treatments (*p* < 0.01). ^2^ *A. solani* (*A. s*), *B. cinerea* (*B. c*), *C. arachidicola* (*C. a*), *F. graminearum* (*F. g*), *P. piricola* (*P. p*), *R. solani* (*R. s*), and *S. sclerotiorum* (*S. s*).

**Table 2:** In vitro precision toxicity determination of EC_50_ values of selected compounds.

Comp.	Fungi ^1^	Regression Equation	R^2^	EC_50_ (μg/mL)	95% Confidence Interval (μg/mL)
**I-1**	*C. a*	y = 3.0551 + 1.9245x	0.9514	10.23	8.00–13.12
	*S. s*	y = 4.3421 + 0.9909x	0.9580	4.61	3.31–6.42
**I-5**	*S. s*	y = 4.1252 + 1.1353x	0.9779	5.89	3.73–9.32
	*C. a*	y = 3.7874 + 1.2820x	0.9962	8.83	8.15–9.56
**I-6**	*S. s*	y = 4.0716 + 1.0393x	0.9628	7.82	5.76–10.63
	*A. s*	y = 2.2428 + 1.9286x	0.9726	26.89	21.90–33.02
**I-7**	*R. s*	y = 2.9379 + 1.6176x	0.9918	18.83	16.89–20.99
	*C. a*	y = 4.0862 + 1.1276x	0.9998	6.46	6.18–6.75
	*S. s*	y = 2.9350 + 1.6853x	0.9984	16.80	16.02–17.62
**I-12**	*A. s*	y = 4.2756 + 0.7412x	0.9789	9.49	7.75–11.62
	*B. c*	y = 3.6982 + 1.1403x	0.9696	13.85	11.37–16.88
	*R. s*	y = 4.4158 + 0.7095x	0.9974	6.66	6.22–7.13
**I-14**	*C. a*	y = 3.8396 + 1.6293x	0.9769	5.15	4.30–6.17
	*S. s*	y = 2.5793 + 2.8281x	0.9543	7.18	5.59–9.21
	*P. p*	y = 3.0468 + 1.5726x	0.9843	17.46	15.06–20.24
Flutriafol	*A. s*	y = 5.6465 + 1.2352x	0.9938	0.30	0.25–0.35
	*B. c*	y = 4.6342 + 0.8530x	0.9946	2.68	2.47–2.91
	*C. a*	y = 5.5458 + 1.2204x	0.9845	0.36	0.29–0.44
	*F. g*	y = 5.0119 + 1.4412x	0.9859	0.98	0.82–1.17
	*P. p*	y = 5.6715 + 1.2163x	0.9880	0.28	0.23–0.34
	*R. s*	y = 5.4127 + 0.5878x	0.9817	0.20	0.15–0.27
	*S. s*	y = 5.5140 + 1.1679x	0.9868	0.36	0.31–0.43
Thifluzamide	*A. s*	y = 4.5223 + 0.6386x	0.9984	5.60	5.35–5.86
	*C. a*	y = 5.0057 + 0.3451x	0.9900	0.96	0.78–1.20
	*R. s*	y = 5.2730 + 0.1924x	0.9855	0.04	0.03–0.05

^1^ *A. solani* (*A. s*), *B. cinerea* (*B. c*), *C. arachidicola* (*C. a*), *F. graminearum* (*F. g*), *P. piricola* (*P. p*), *R. solani* (*R. s*), and *S. sclerotiorum* (*S. s*).

## Data Availability

The data are contained within the article; further inquiries can be directed to the corresponding authors.

## Supplementary Materials

The following supporting information can be downloaded at https://www.mdpi.com/article/10.3390/microorganisms13071588/s1, Figures S1–S6: The ^1^HNMR and ^13^CNMR spectrum of intermediates compound; Figures S7–S72: The ^1^HNMR, ^13^CNMR, and HRMS spectrum of target compounds; Figures S73–S79: KEGG pathway analysis of DEGs.
