# Supplementary material for: Discovery of 4,5,6,7-Tetrahydrothieno [3,2-b] Pyridine as Novel Fungicide Lead Scaffold
Source: Microorganisms. 2025 Jul 5;13(7):1588. doi: 10.3390/microorganisms13071588 (PMC12300993; doi:10.3390/microorganisms13071588)
Supplement: Supplementary file 1 [file microorganisms-13-01588-s001.zip › microorganisms-3674164-supplementary.pdf]

Supporting Information

Discovery of 4,5,6,7-tetrahydrothieno [3,2-b] pyridine as Novel Fungicide Lead Scaffold

Ke Chen [1,2], Difan Deng [3], Yupeng Yin [3], Dongmei Xi [2], Phumbum Park [1], Wei Gao [3], Rui Liu [1,*] and Kang Lei [3,*]

[1] Department of Biotechnology, The University of Suwon, Hwaseong 18323, Gyeonggi-do, Republic of Korea; chenke_sd@163.com (K.C.); pbpark@suwon.ac.kr (P.P.)
[2] College of Life Sciences, Linyi University, Linyi 276005, China; xidongmei@lyu.edu.cn
[3] School of Pharmaceutical Sciences and Food Engineering, Liaocheng University, Liaocheng 252059, China; 2022406865@stu.lcu.edu.cn (D.D.); 2022406950@stu.lcu.edu.cn (Y.Y.); gaowei@lcu.edu.cn (W.G.)
* Correspondence: liurcau@suwon.ac.kr (R.L.); leikang@lcu.edu.cn (K.L.)

1. **The ^{1}HNMR, ^{13}CNMR, and HRMS Spectrum of Representative Intermediates**

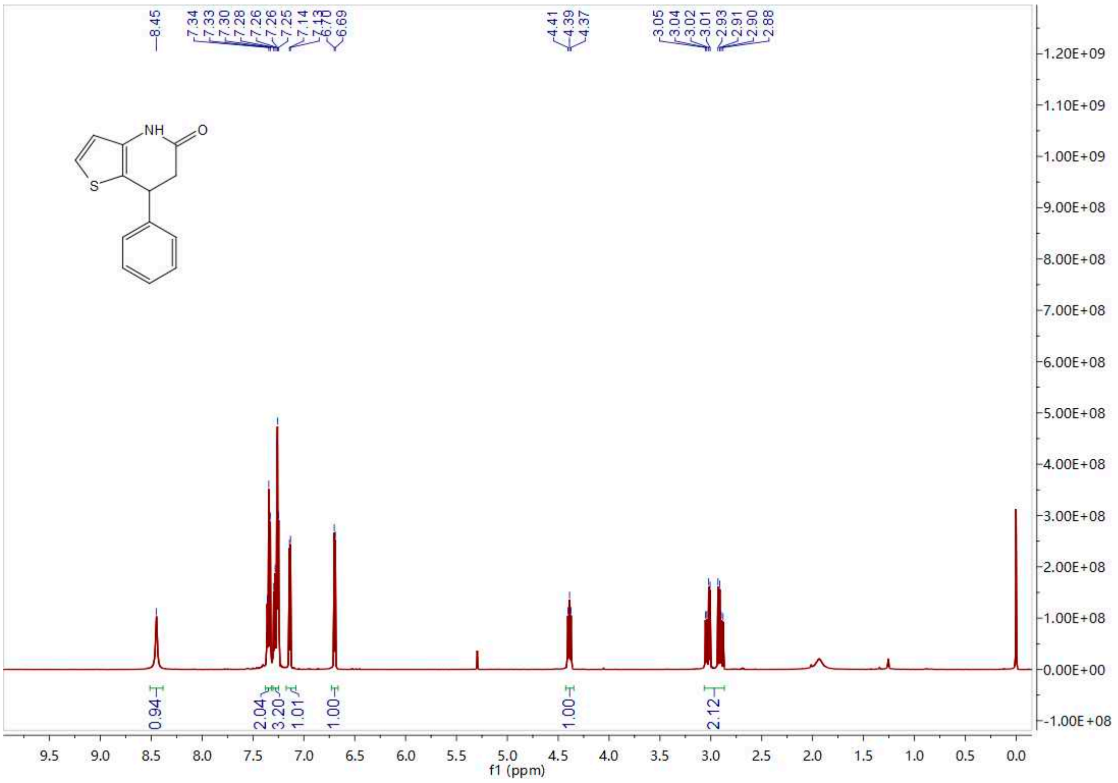

Figure S1 ^{1}HNMR of intermediate compound **3a**

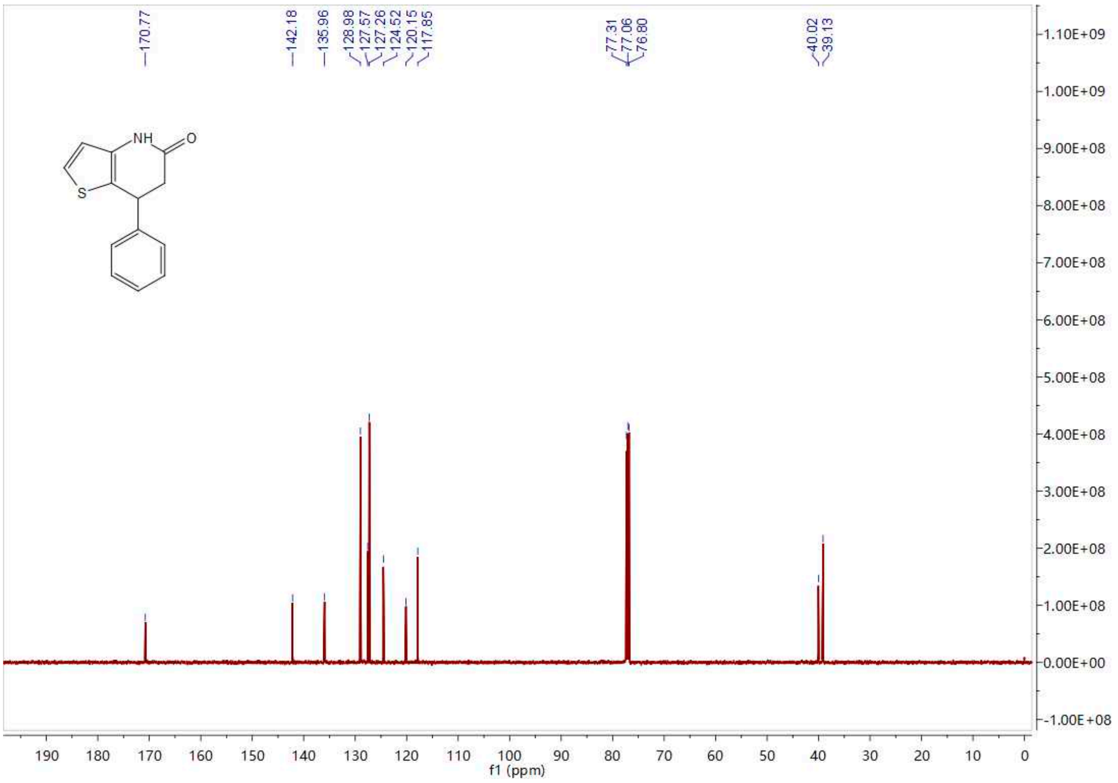

Figure S2 ^{13}CNMR of intermediate compound **3a**

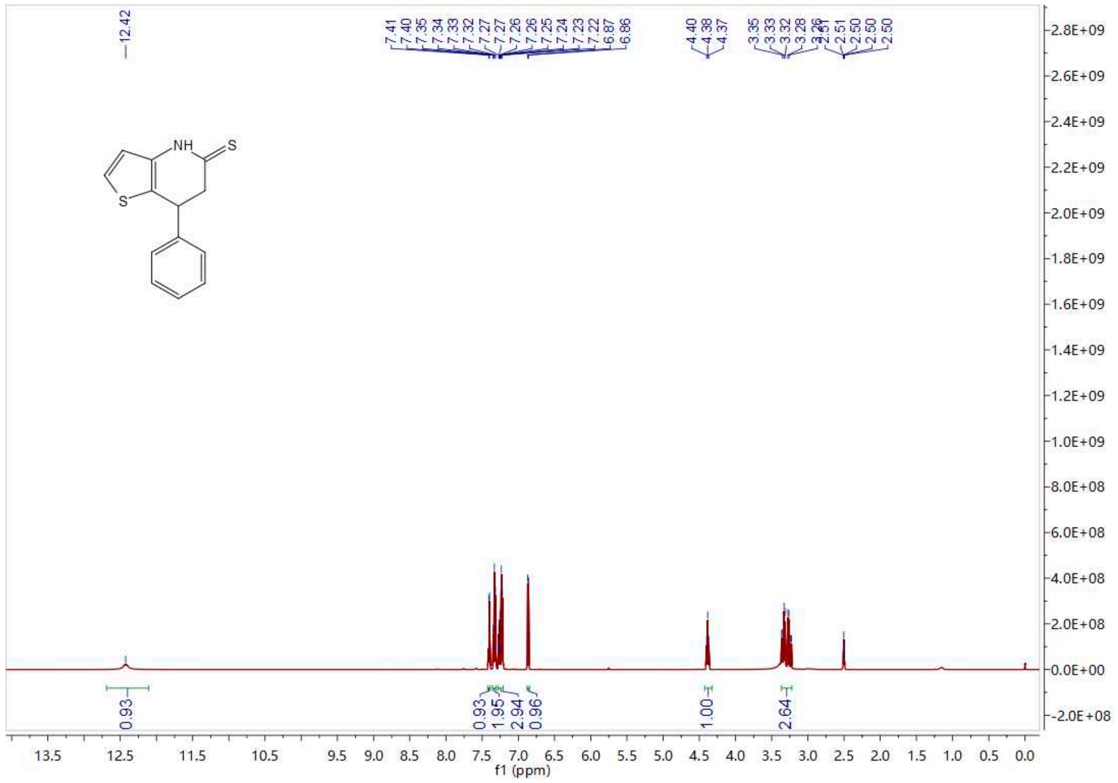

Figure S3 ^{1}HNMR of intermediate compound **4a**

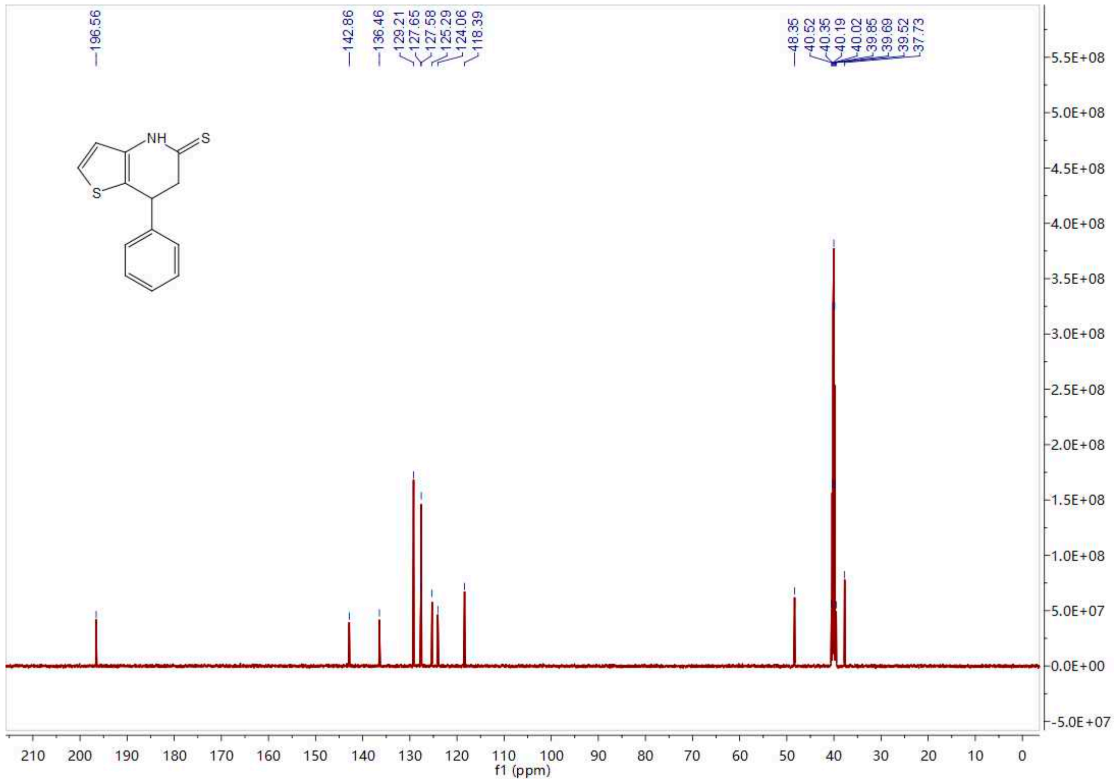

Figure S4 ^{13}CNMR of intermediate compound **4a**

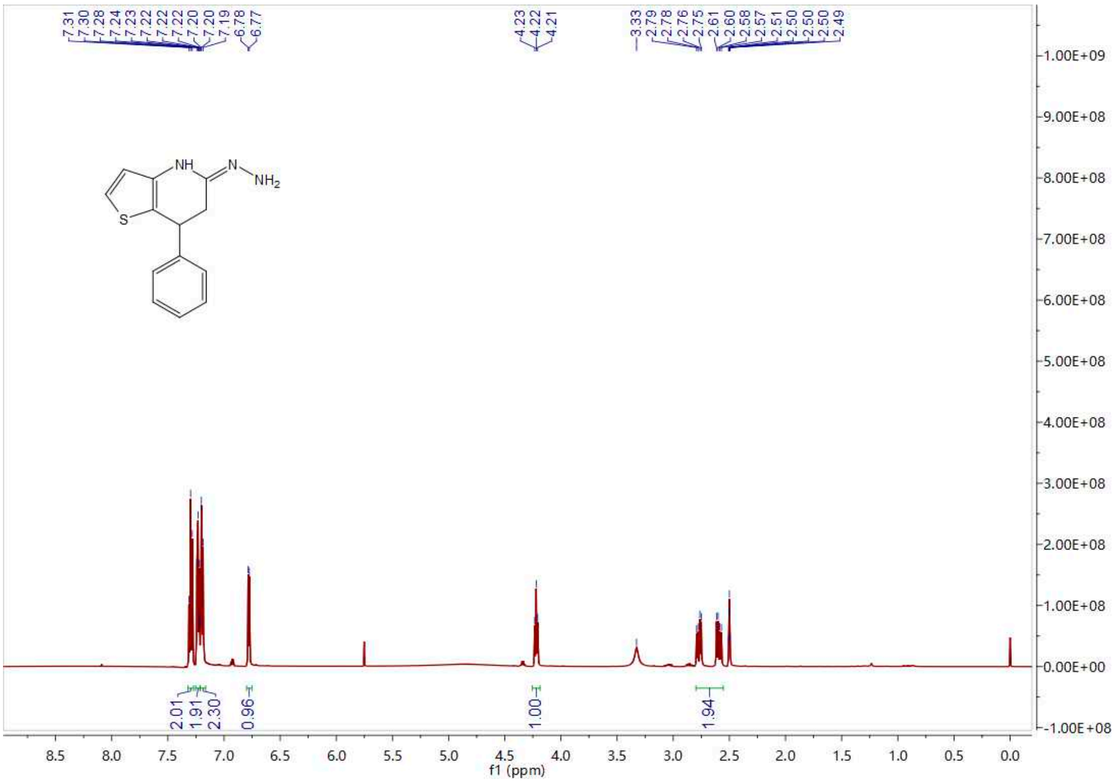

Figure S5 ^{1}HNMR of intermediate compound **5a**

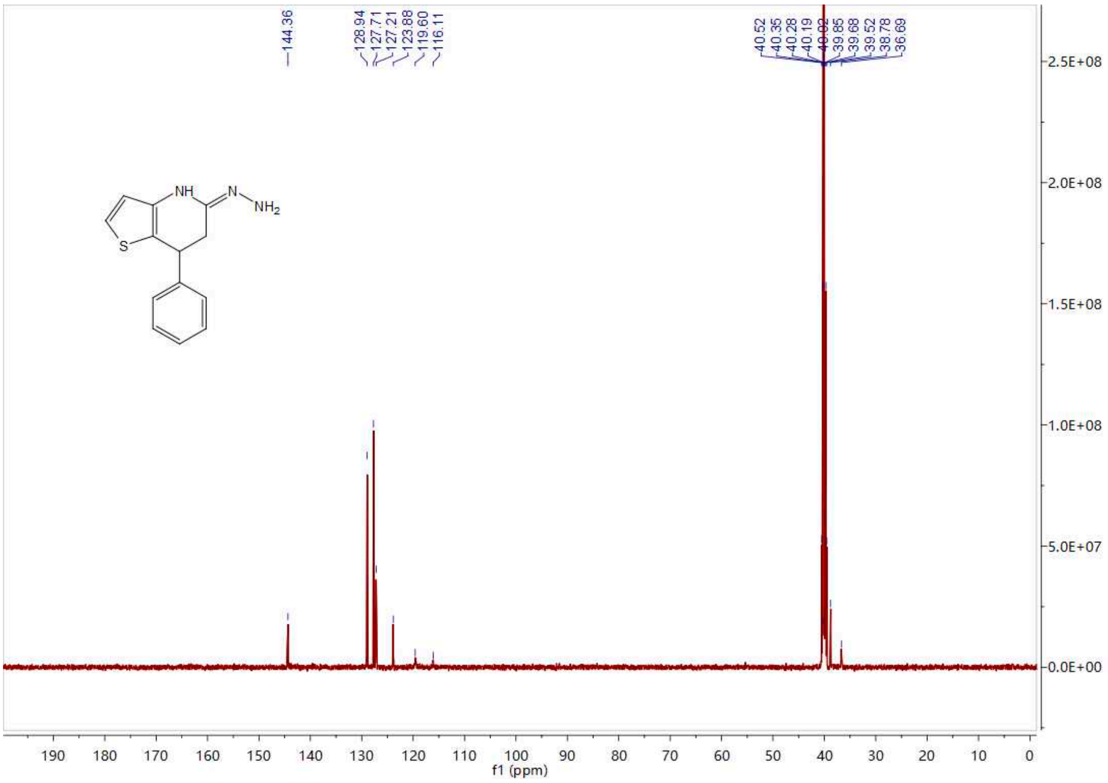

Figure S6 ^{13}CNMR of intermediate compound **5a**

2. The ^{1}HNMR, ^{13}CNMR, and HRMS Spectrum of Target Compounds

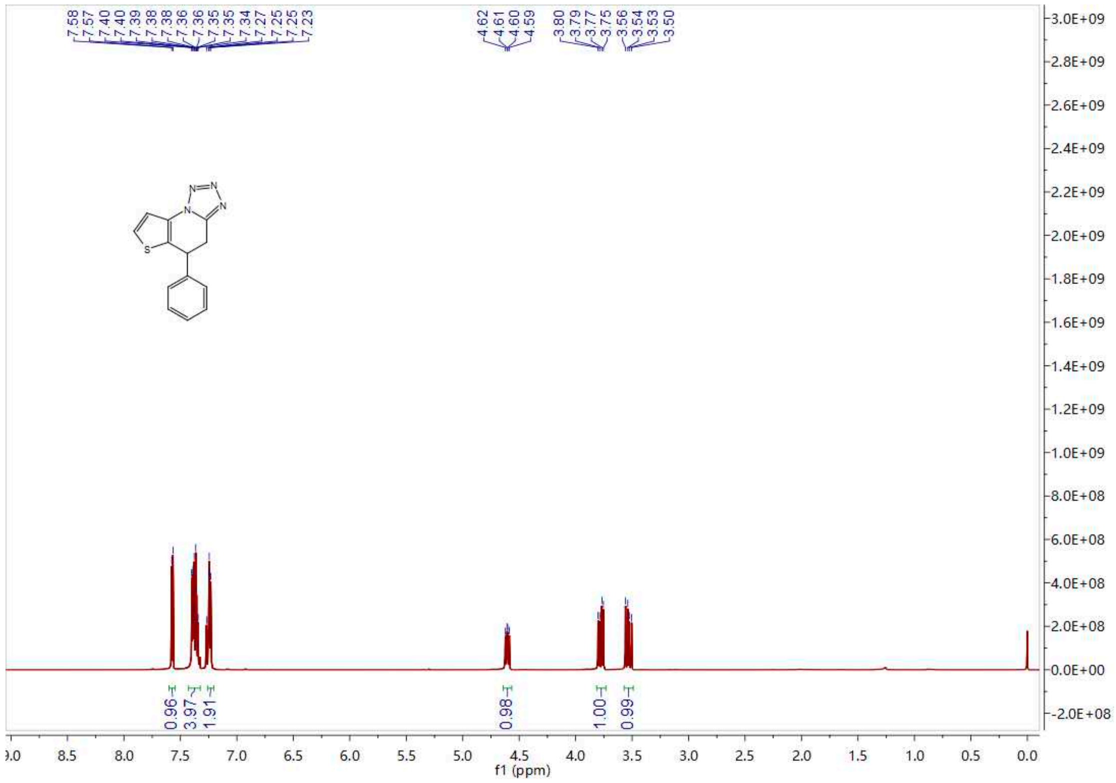

Figure S7 ^{1}HNMR of target compound **I-1**

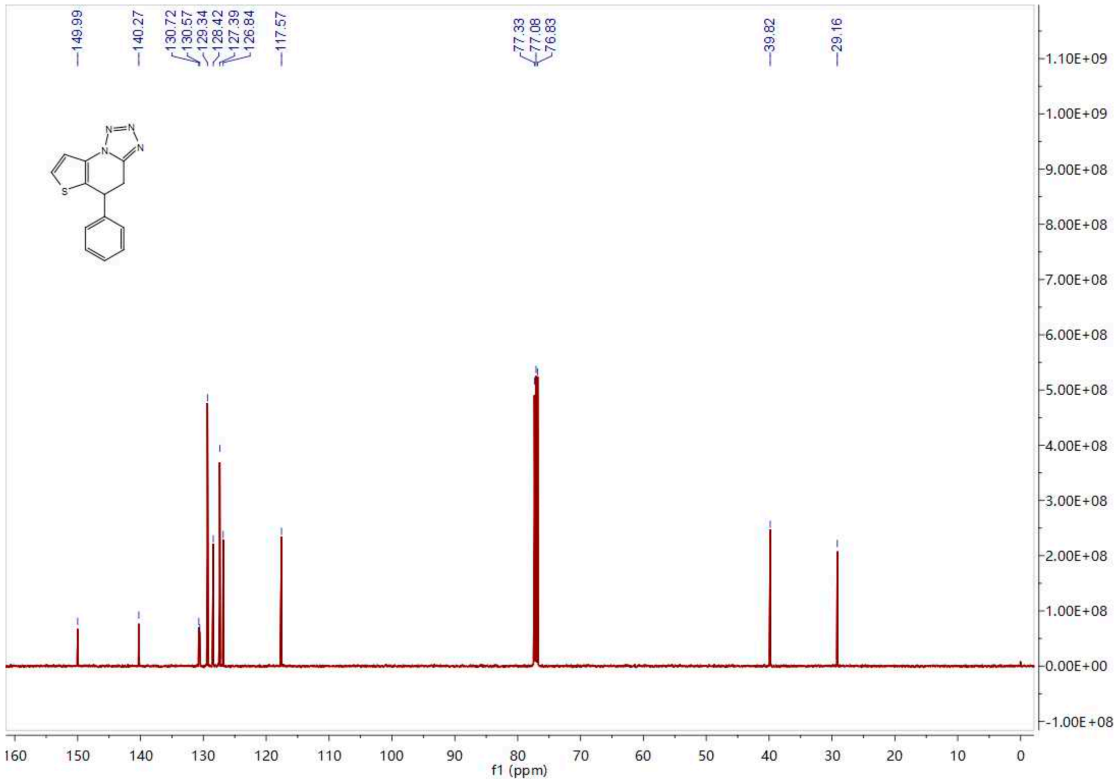

Figure S8 ^{13}CNMR of target compound **I-1**

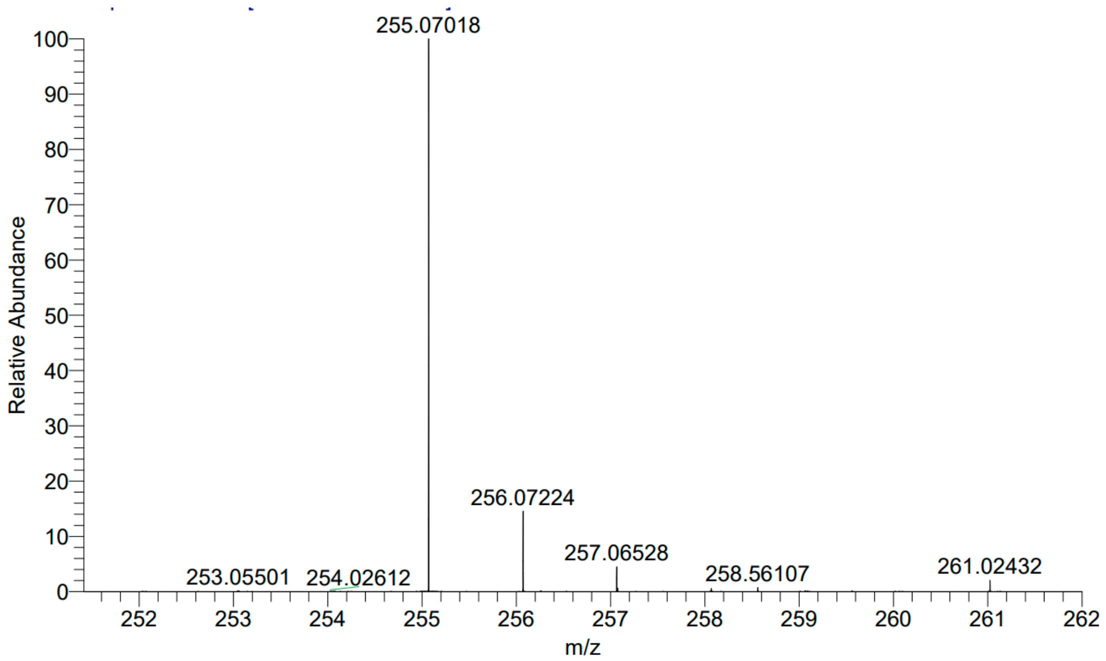

Figure S9 HRMS of target compound **I-1**

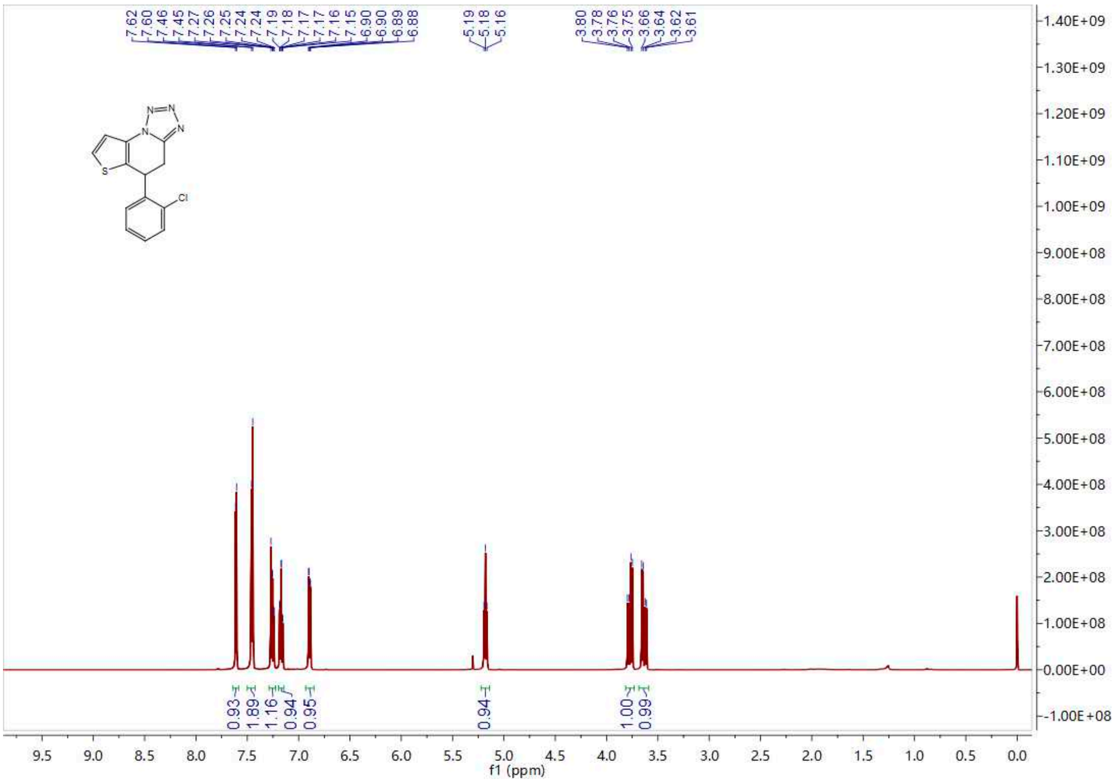

Figure S10 ^{1}HNMR of target compound **I-2**

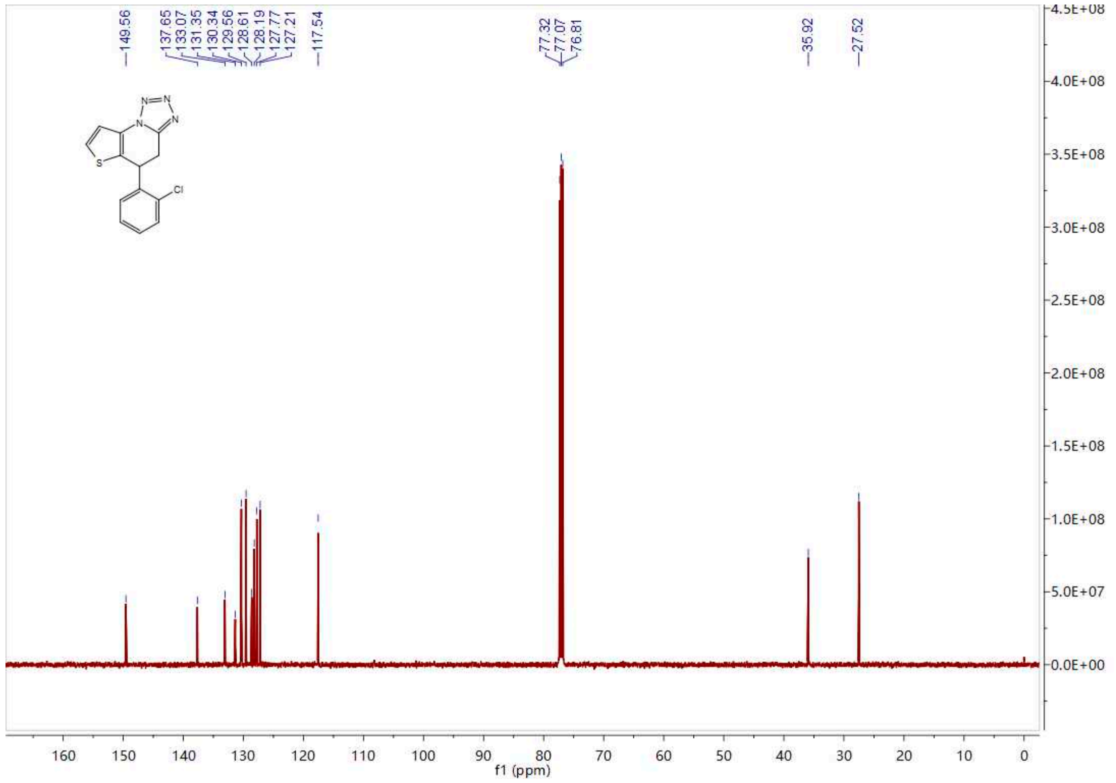

Figure S11 ^{13}CNMR of target compound **I-2**

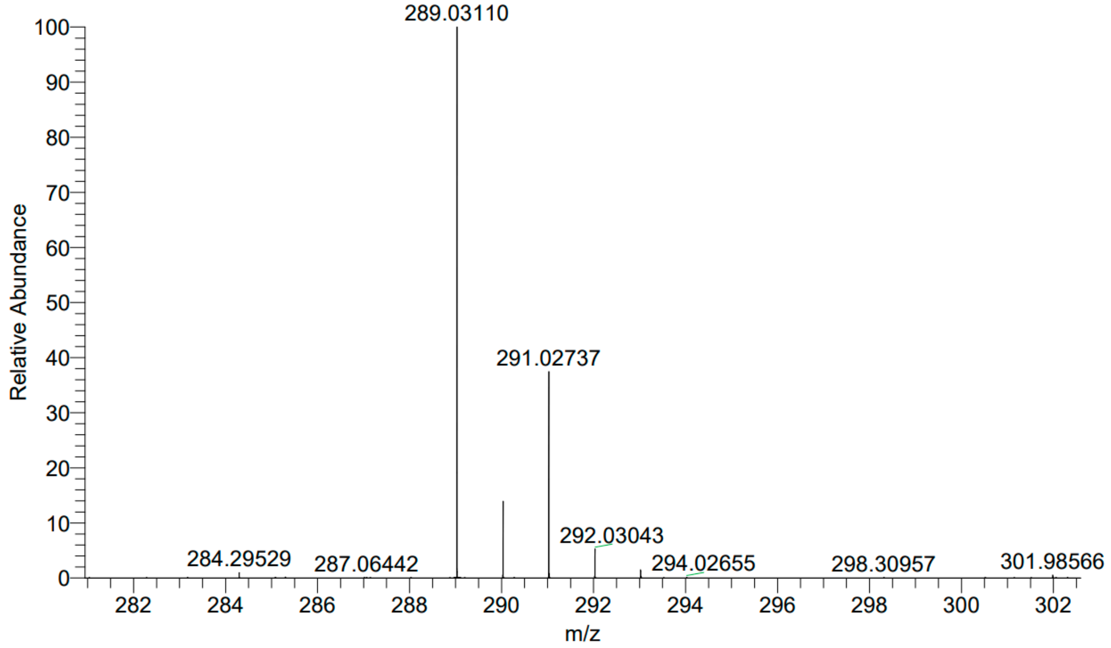

Figure S12 HRMS of target compound **I-2**

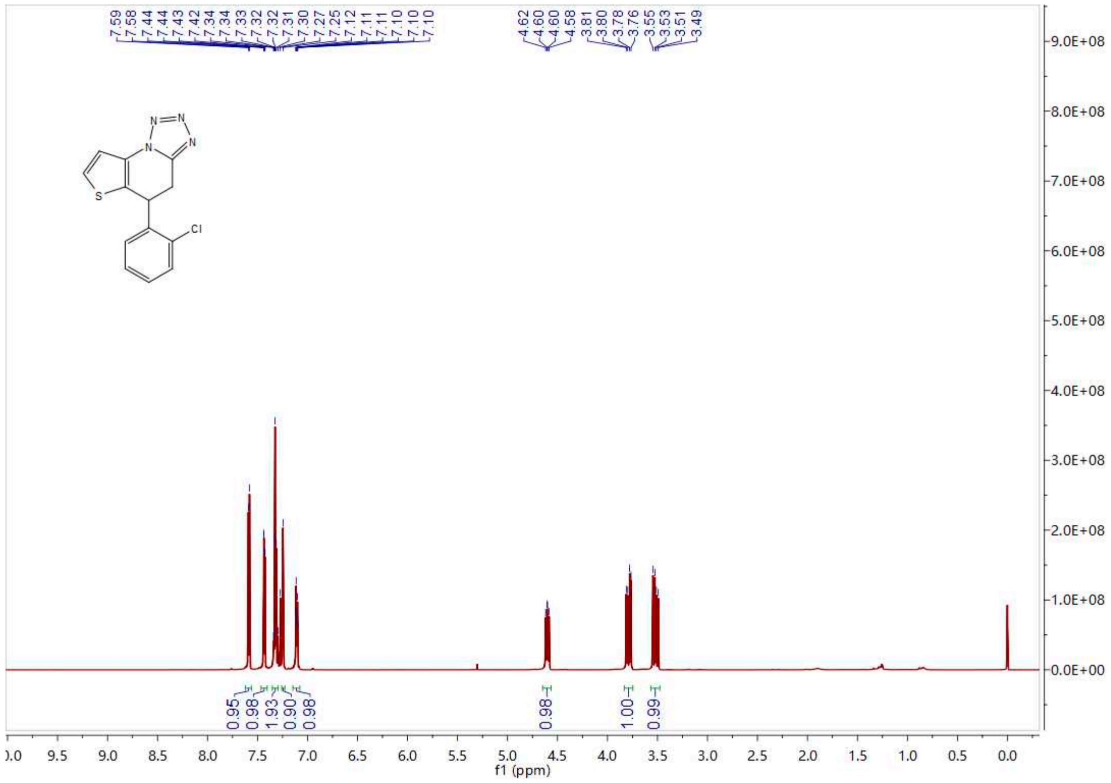

Figure S13 ^{1}HNMR of target compound **I-3**

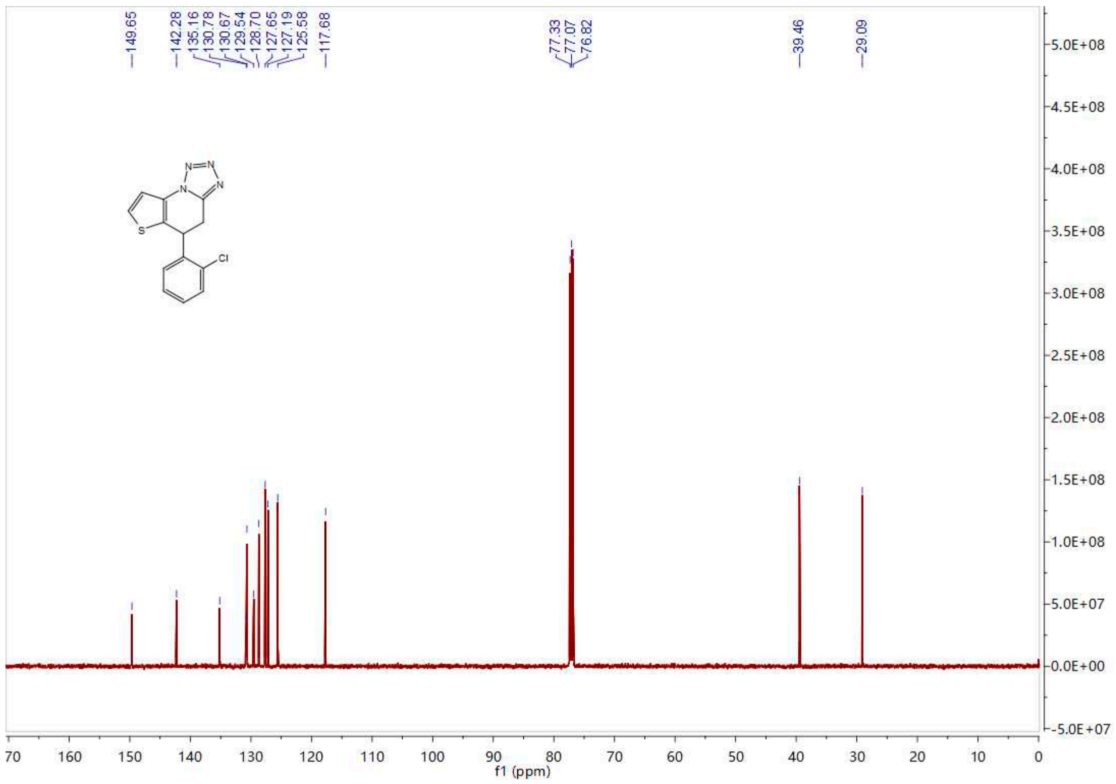

Figure S14 ^{13}CNMR of target compound **I-3**

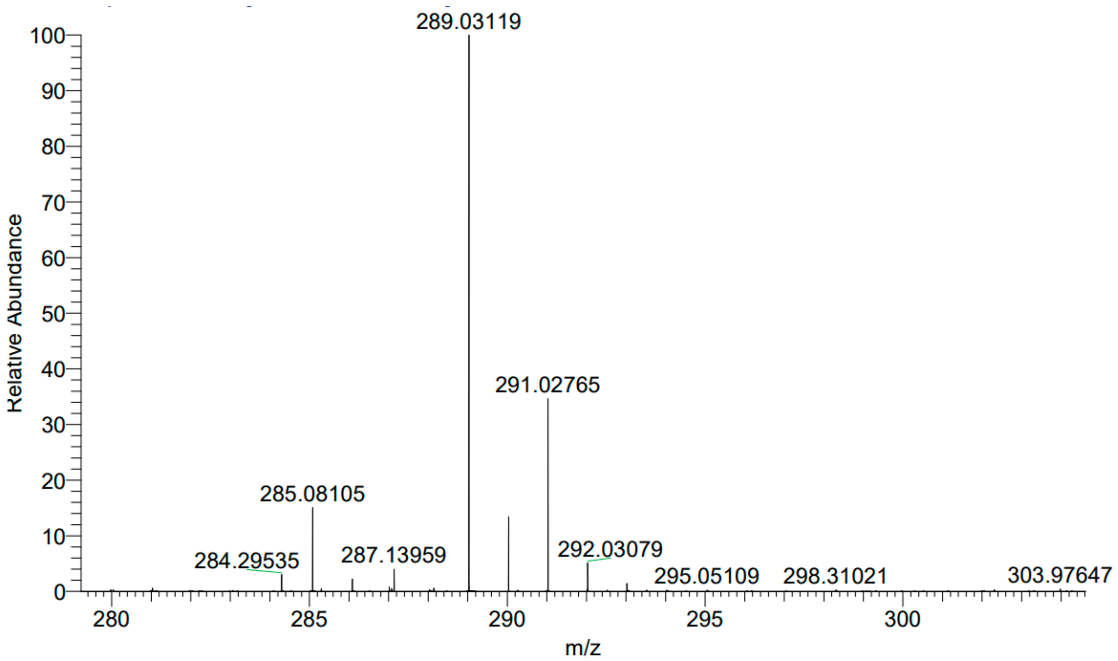

Figure S15 HRMS of target compound **I-3**

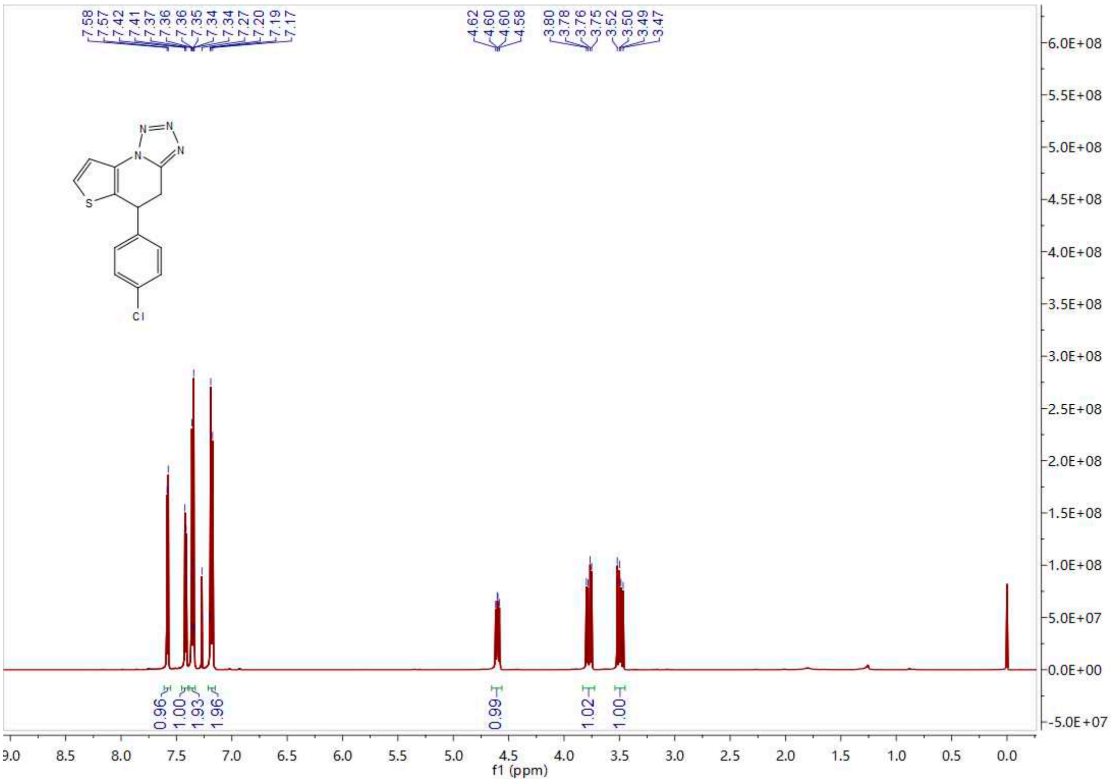

Figure S16 ^{1}HNMR of target compound **I-4**

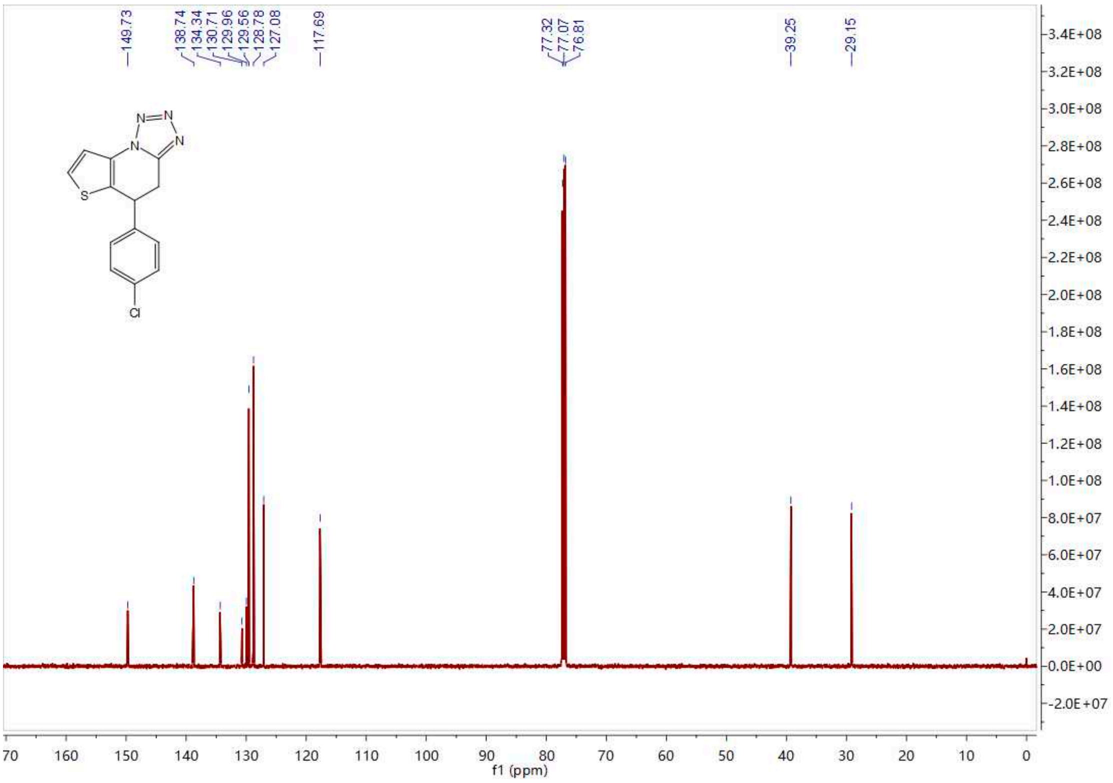

Figure S17 ^{13}CNMR of target compound **I-4**

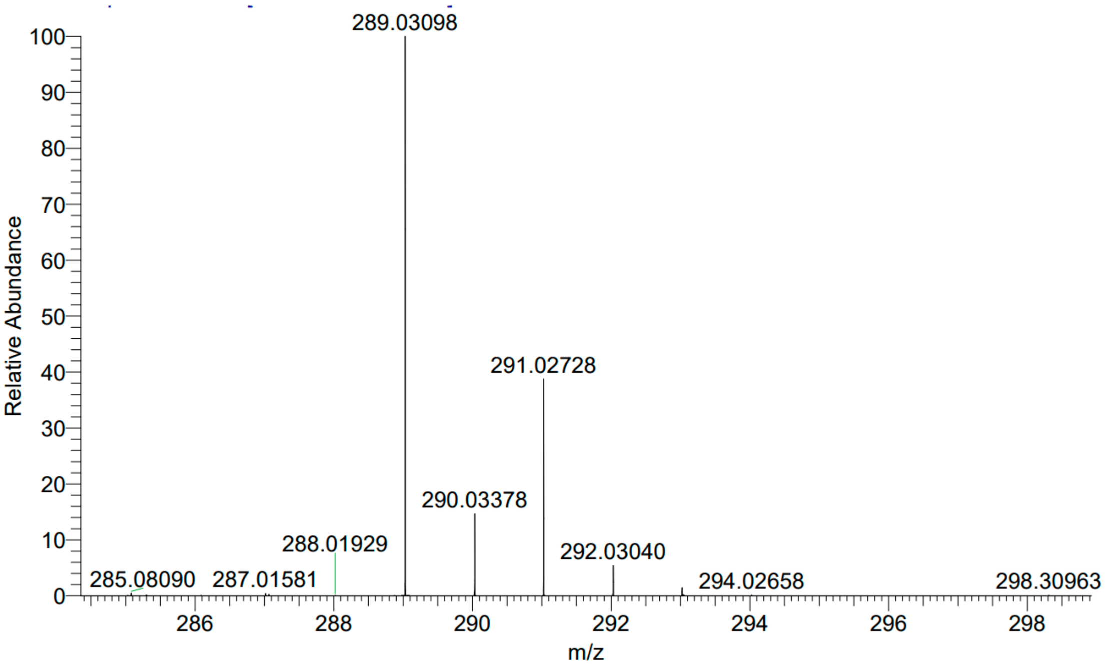

Figure S18 HRMS of target compound **I-4**

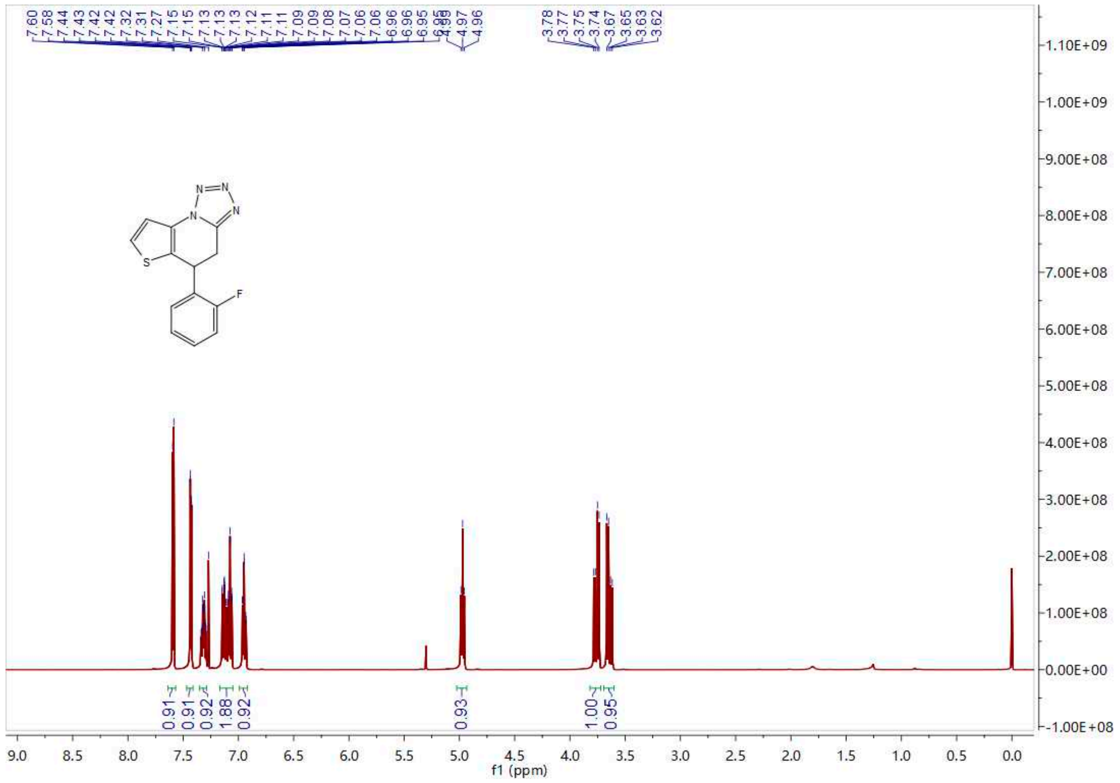

Figure S19 ^{1}HNMR of target compound **I-5**

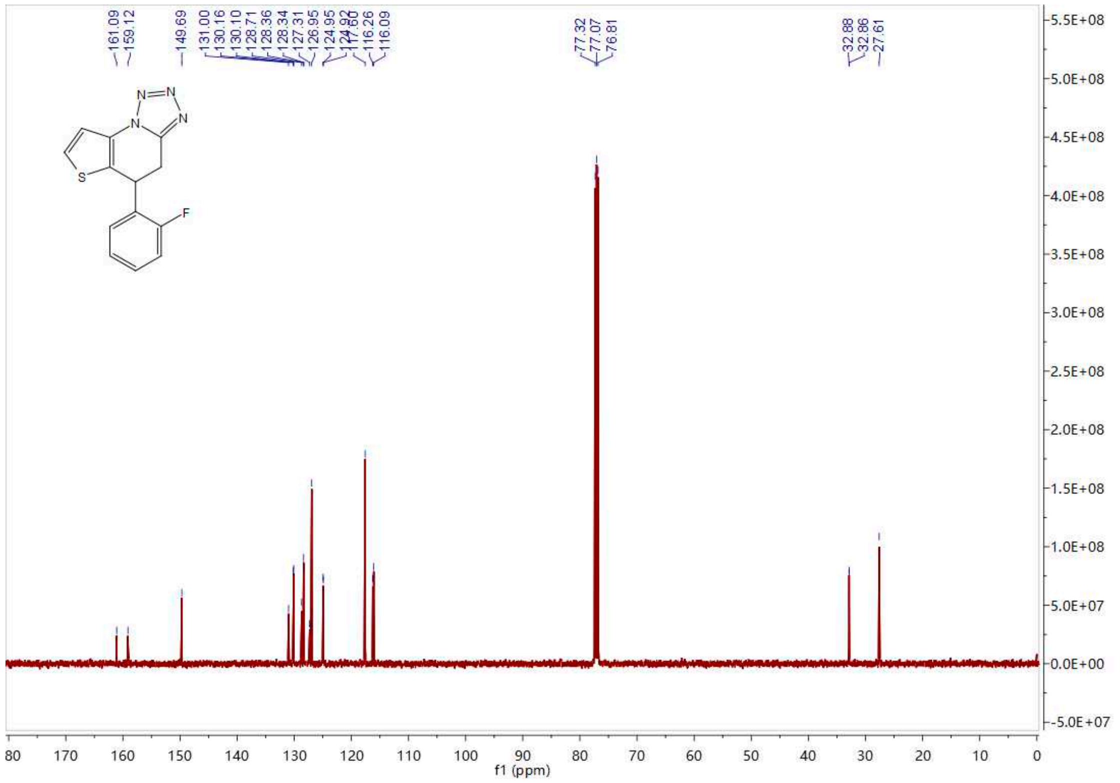

Figure S20 ^{13}CNMR of target compound **I-5**

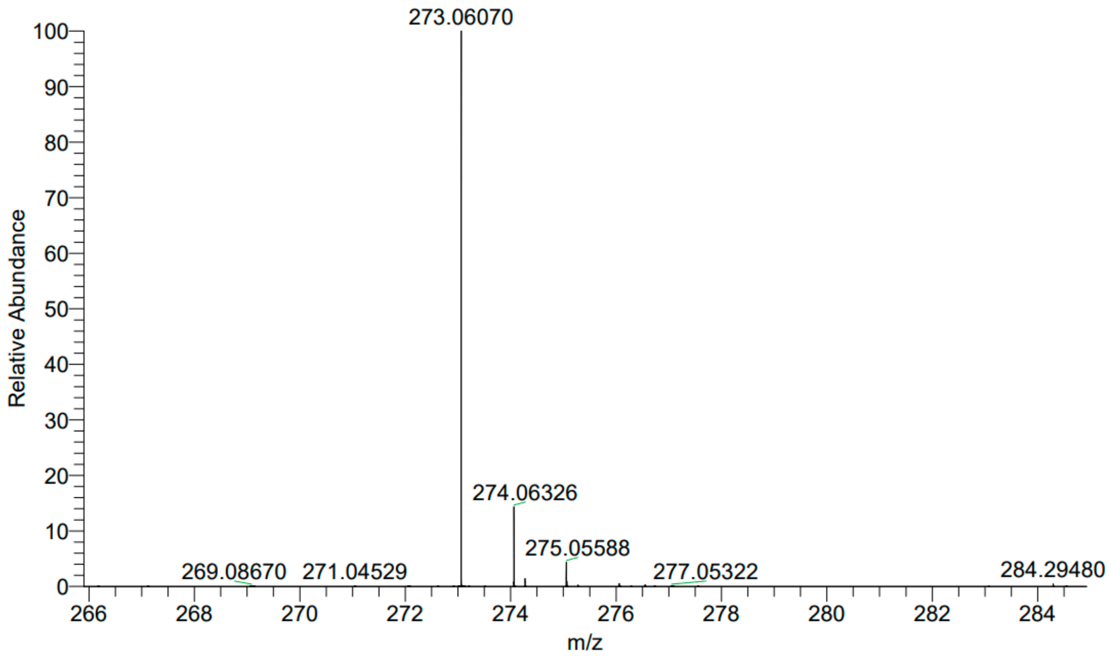

Figure S21 HRMS of target compound **I-5**

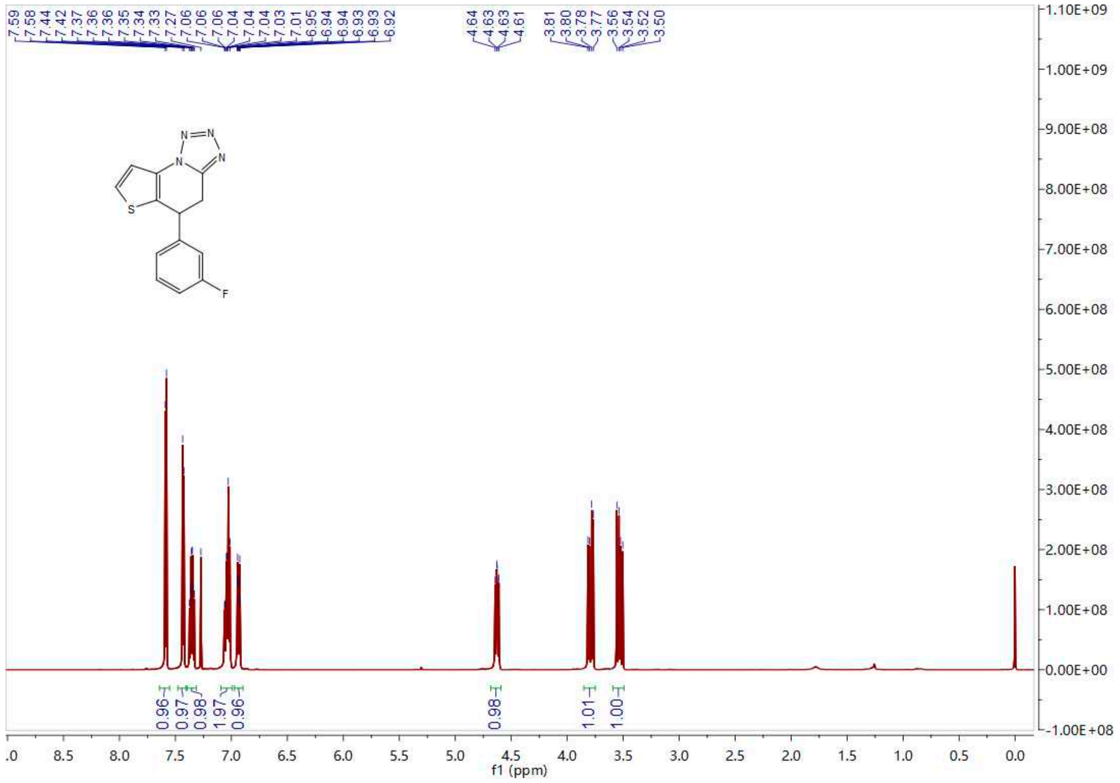

Figure S22 ^{1}HNMR of target compound **I-6**

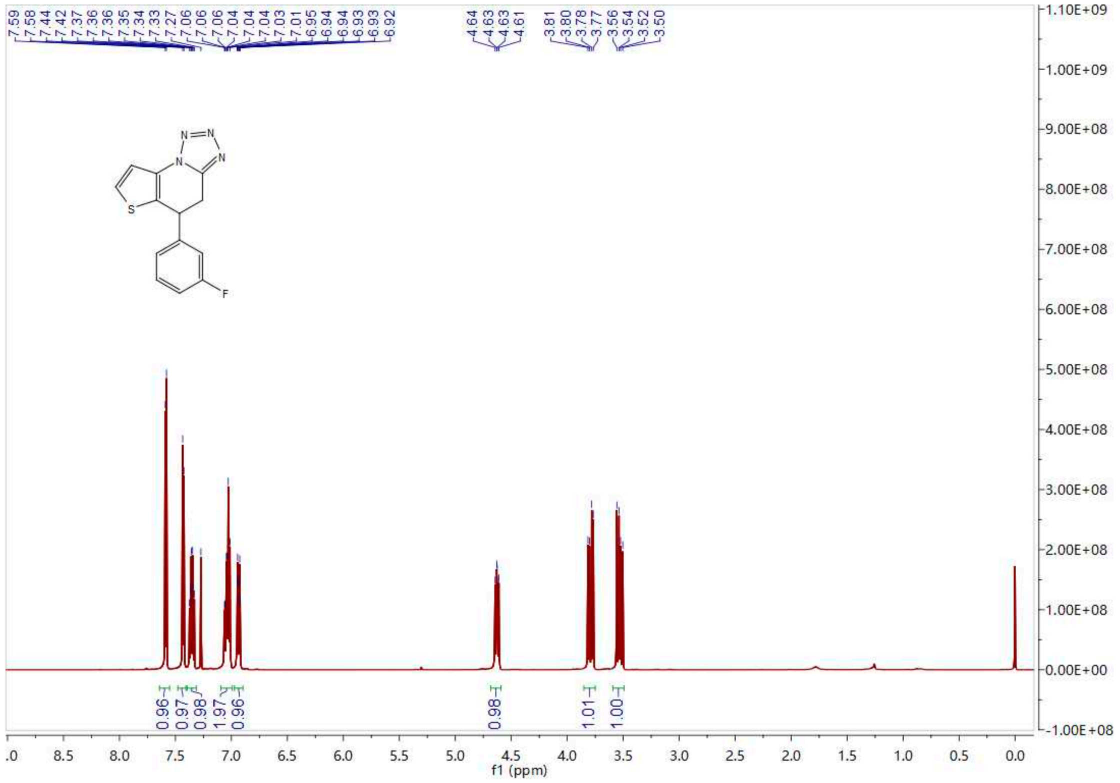

Figure S23 ^{13}CNMR of target compound **I-6**

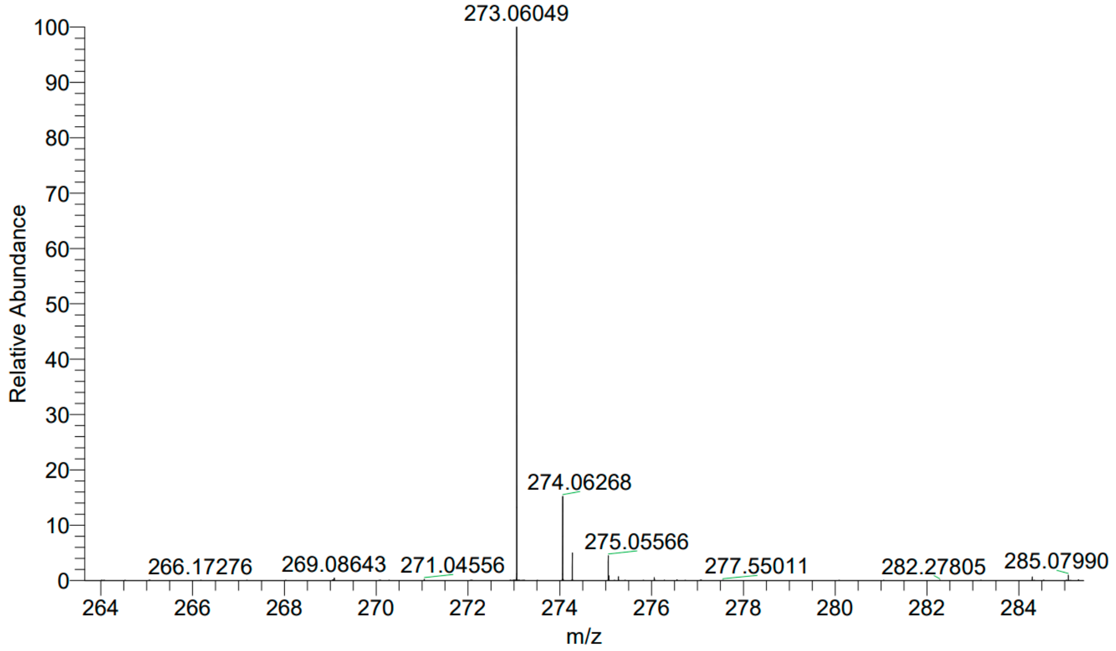

Figure S24 HRMS of target compound **I-6**

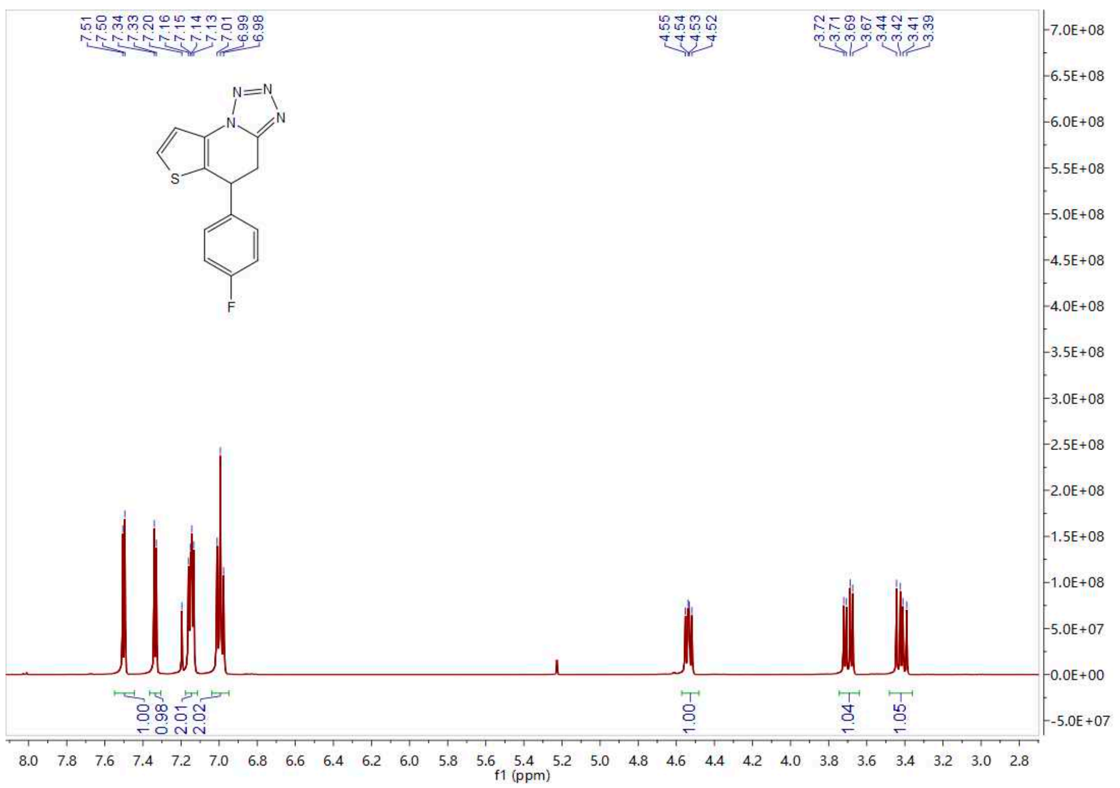

Figure S25 ^{1}HNMR of target compound **I-7**

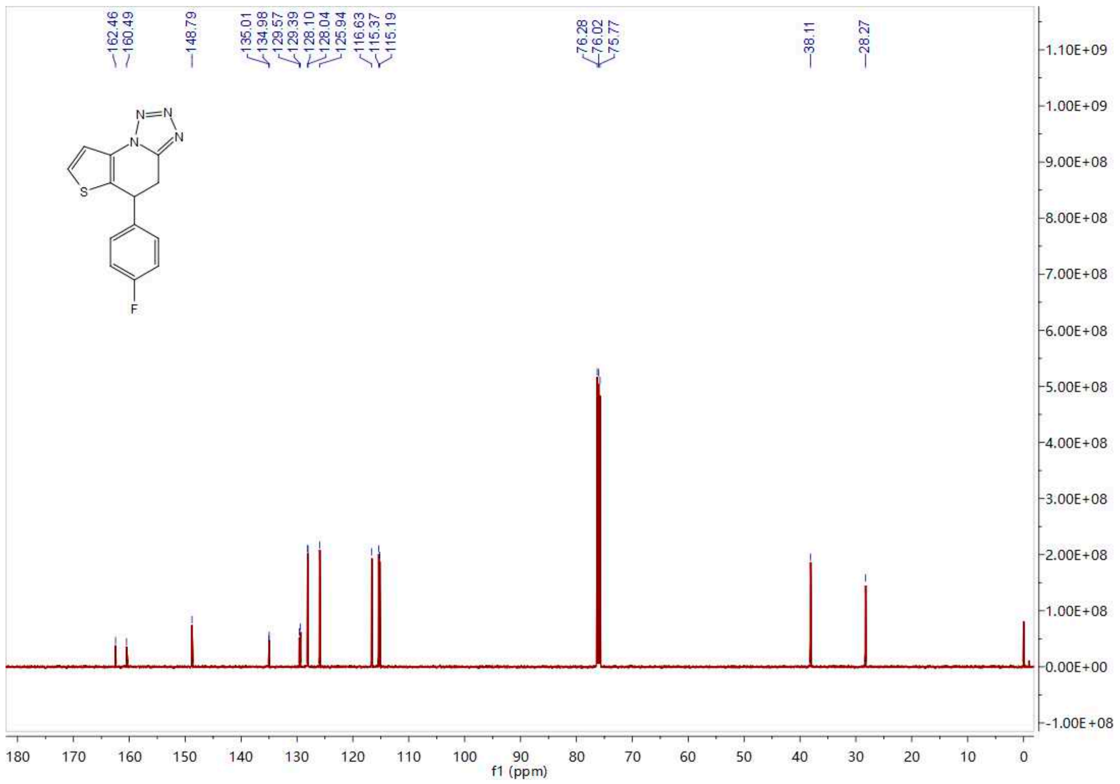

Figure S26 ^{13}CNMR of target compound **I-7**

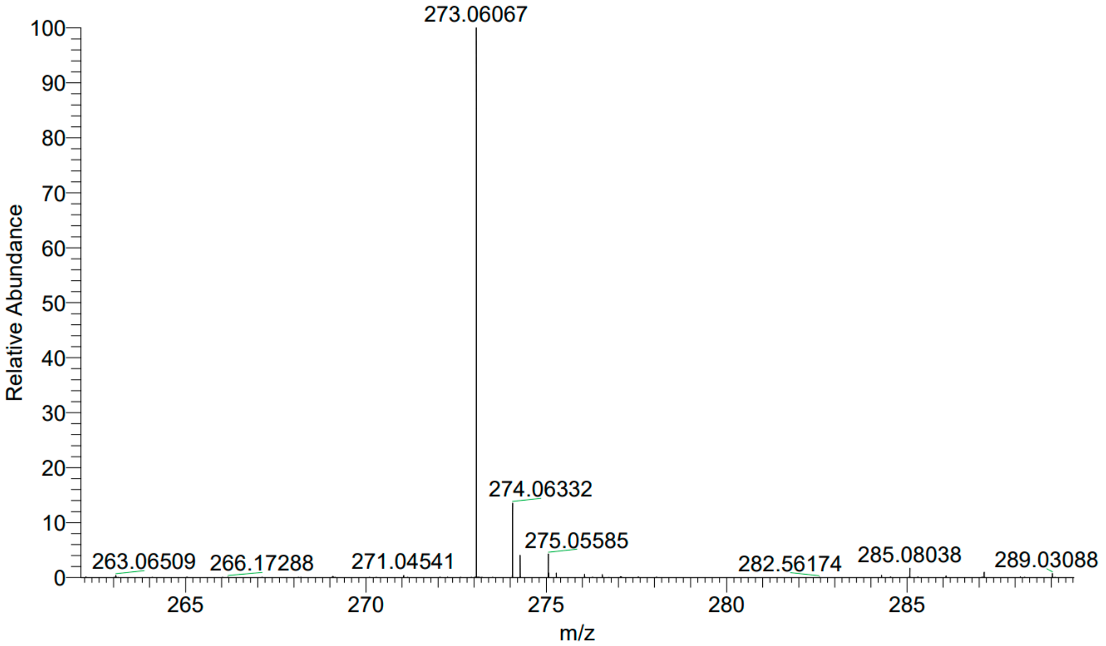

Figure S27 HRMS of target compound **I-7**

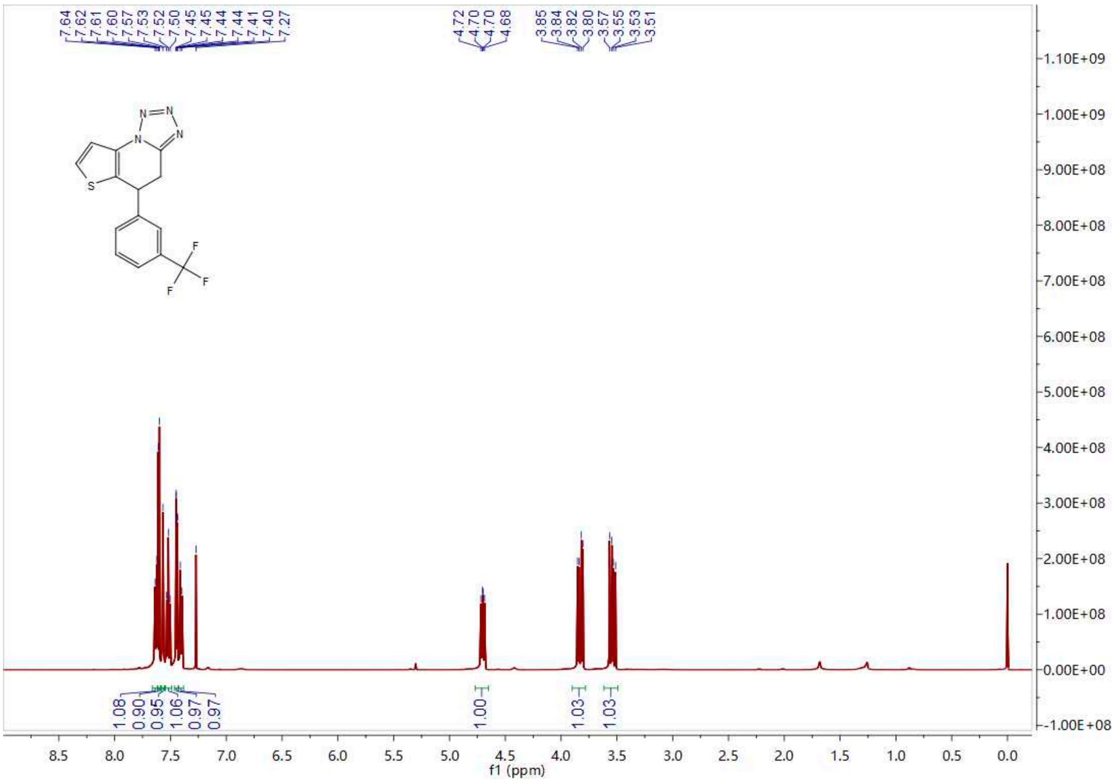

Figure S28 ^{1}HNMR of target compound **I-8**

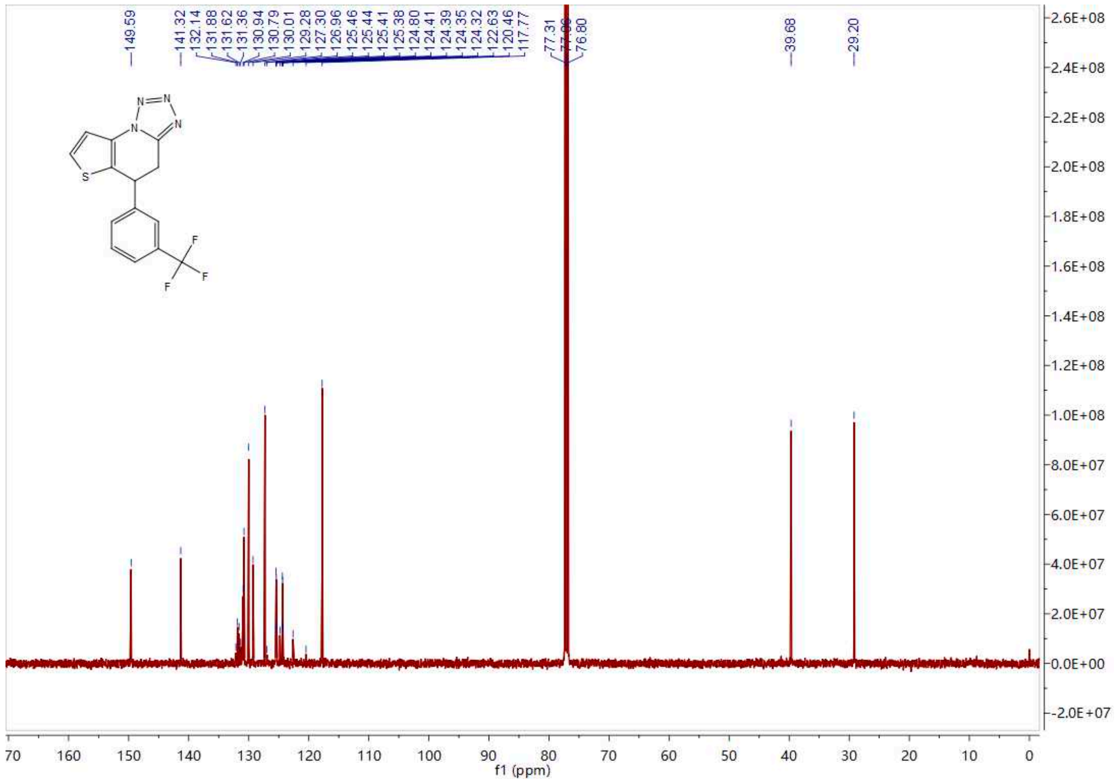

Figure S29 ^{13}CNMR of target compound **I-8**

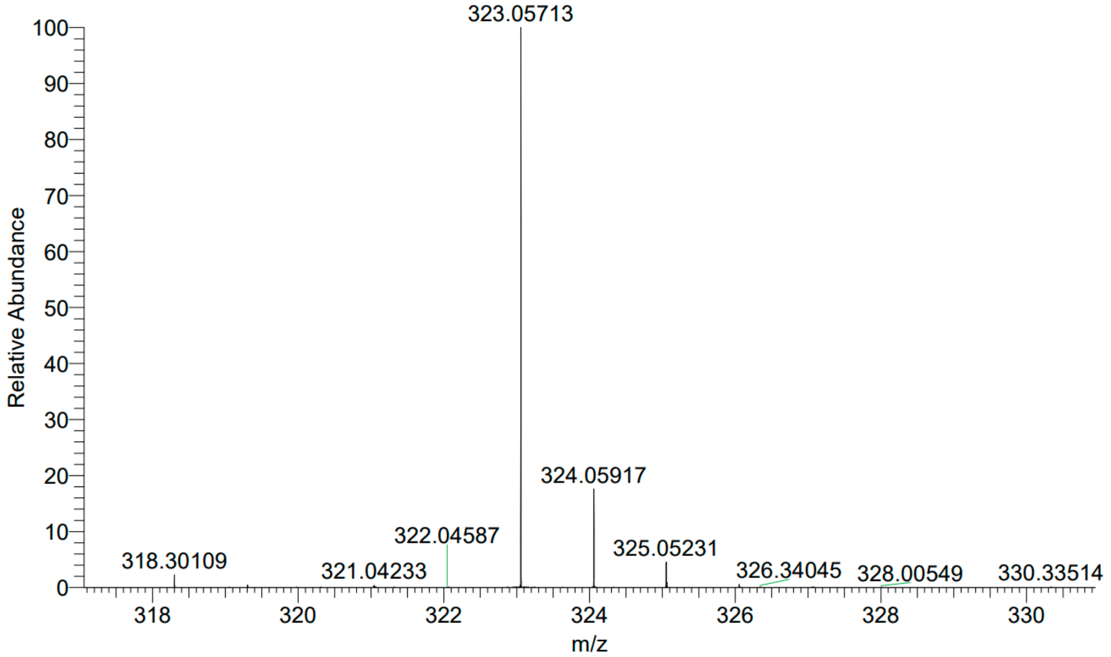

Figure S30 HRMS of target compound **I-8**

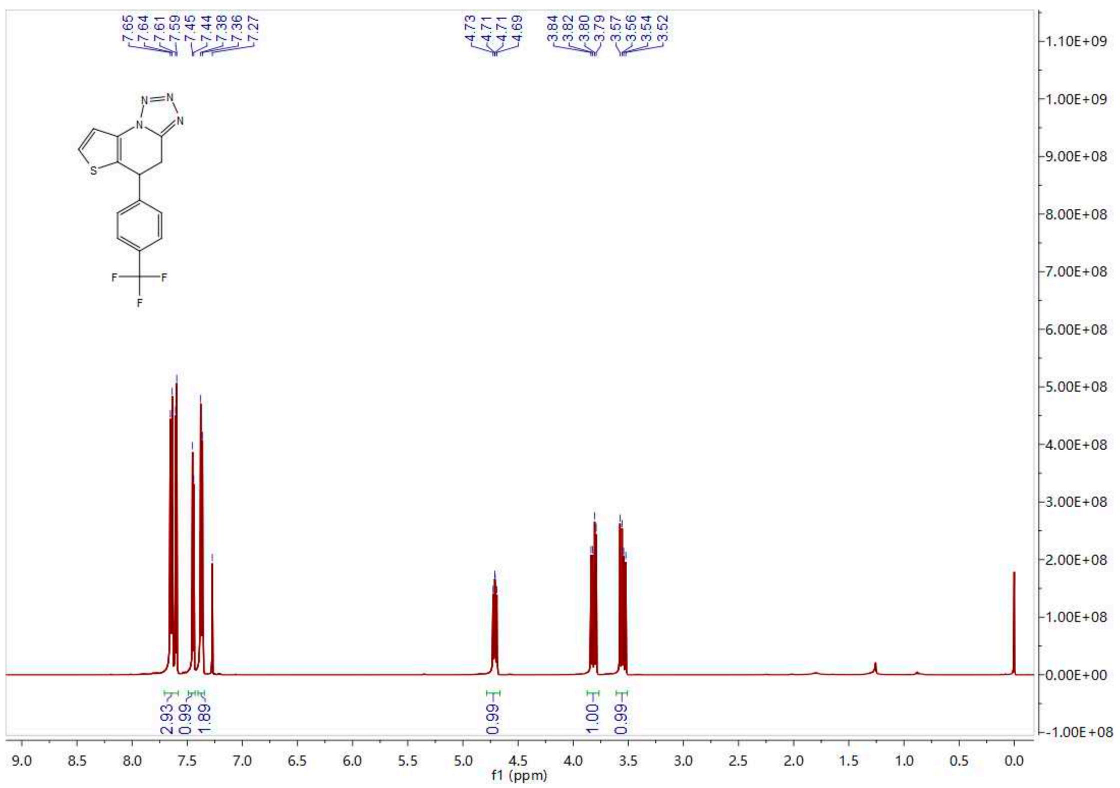

Figure S31 ^{1}HNMR of target compound **I-9**

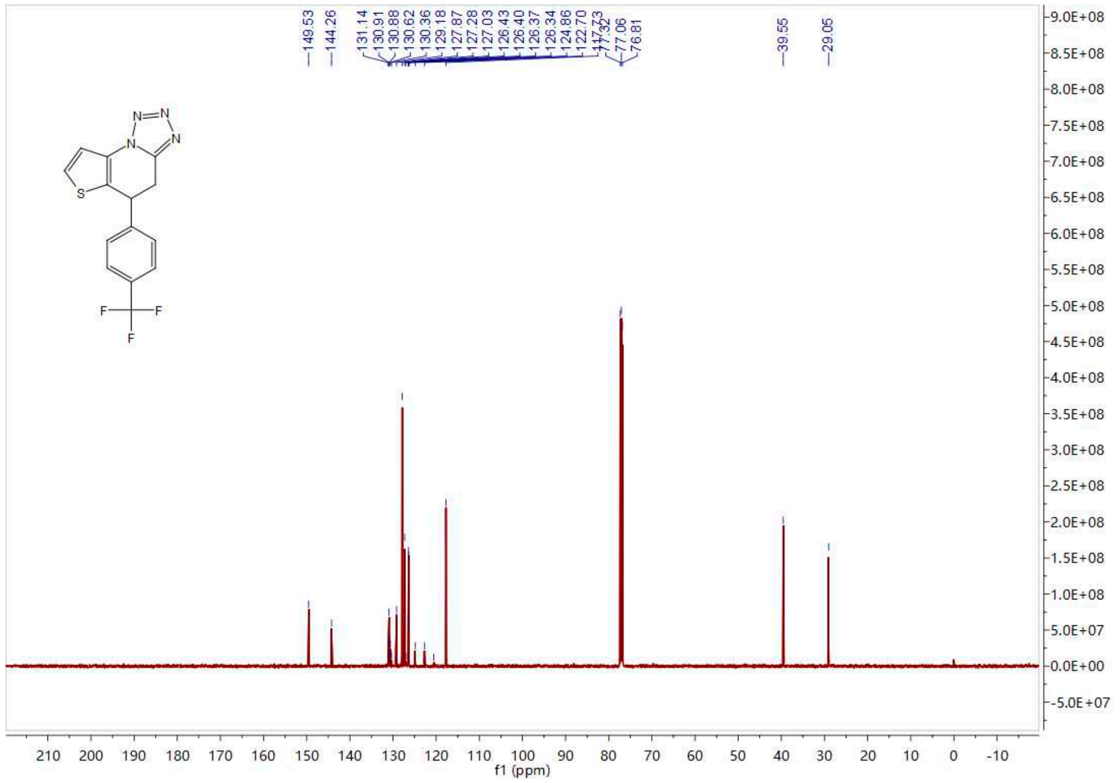

Figure S32 ^{13}CNMR of target compound **I-9**

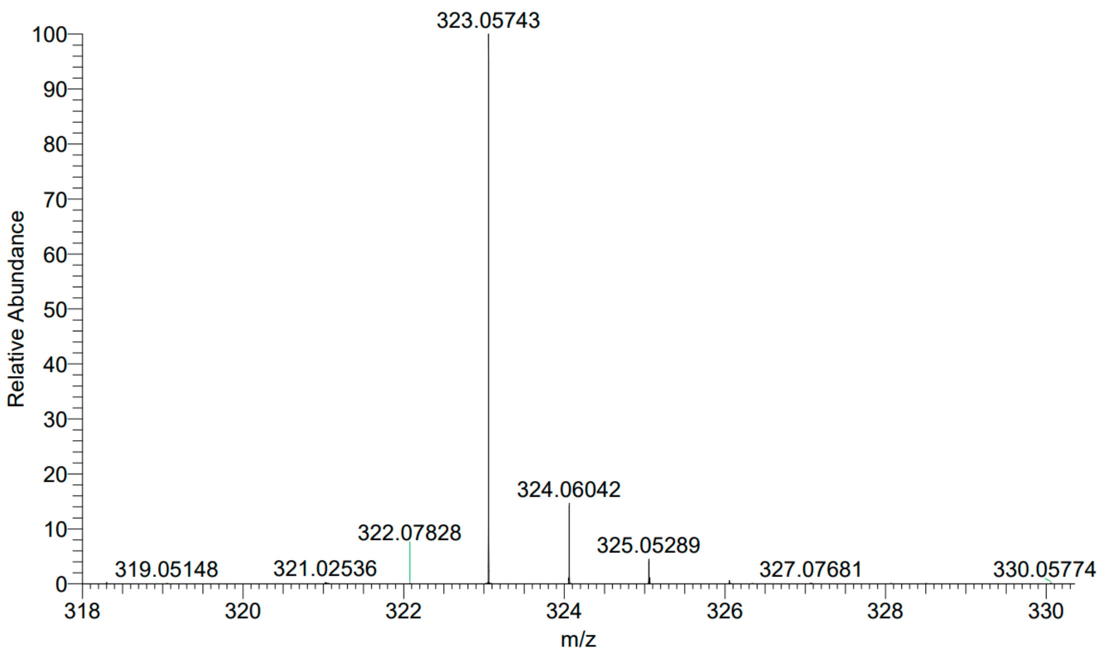

Figure S33 HRMS of target compound **I-9**

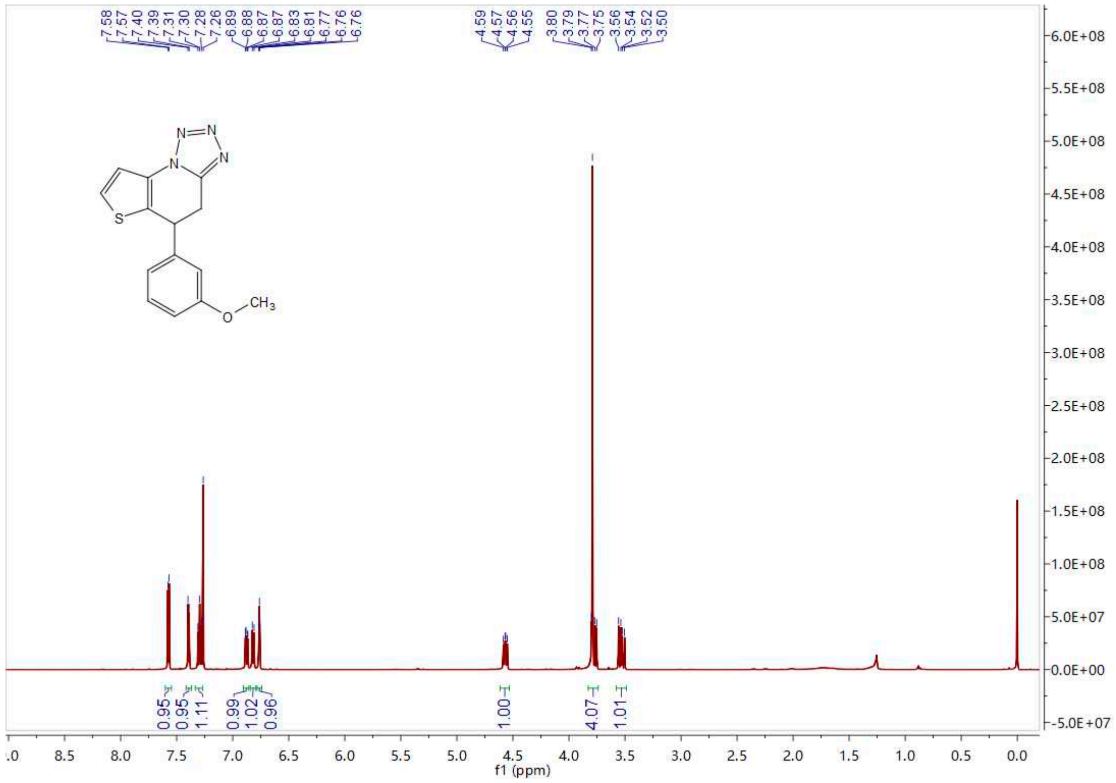

Figure S34 ^{1}HNMR of target compound **I-10**

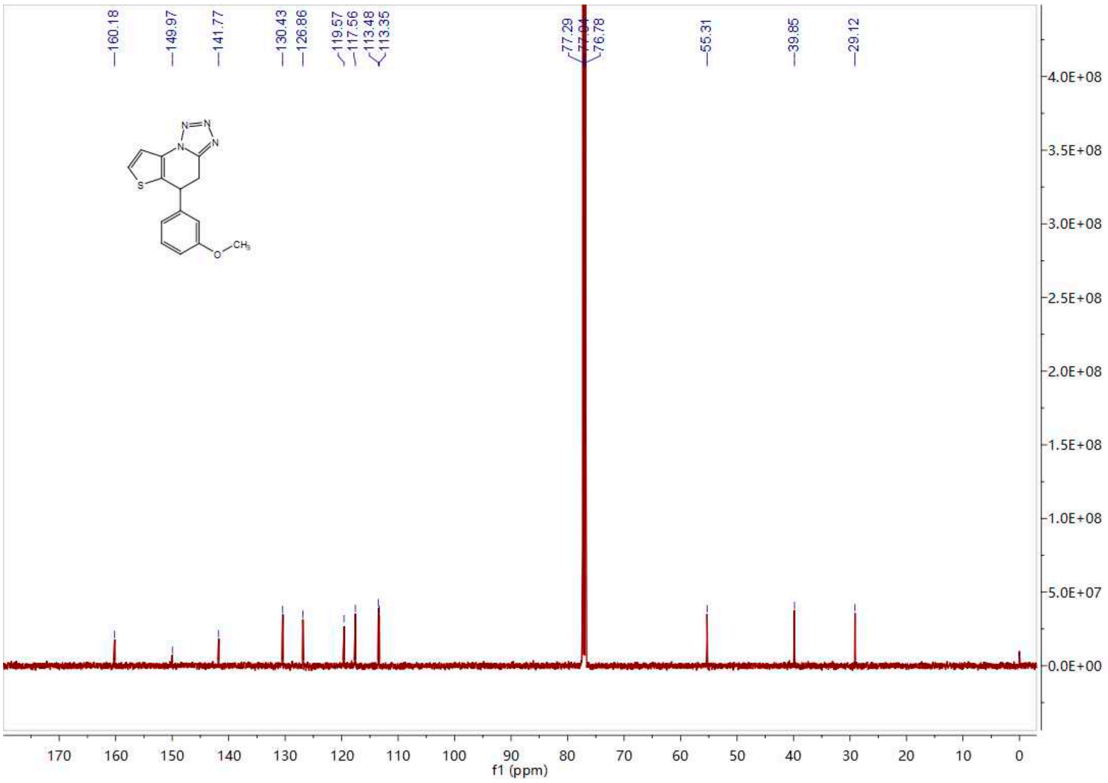

Figure S35 ^{13}CNMR of target compound **I-10**

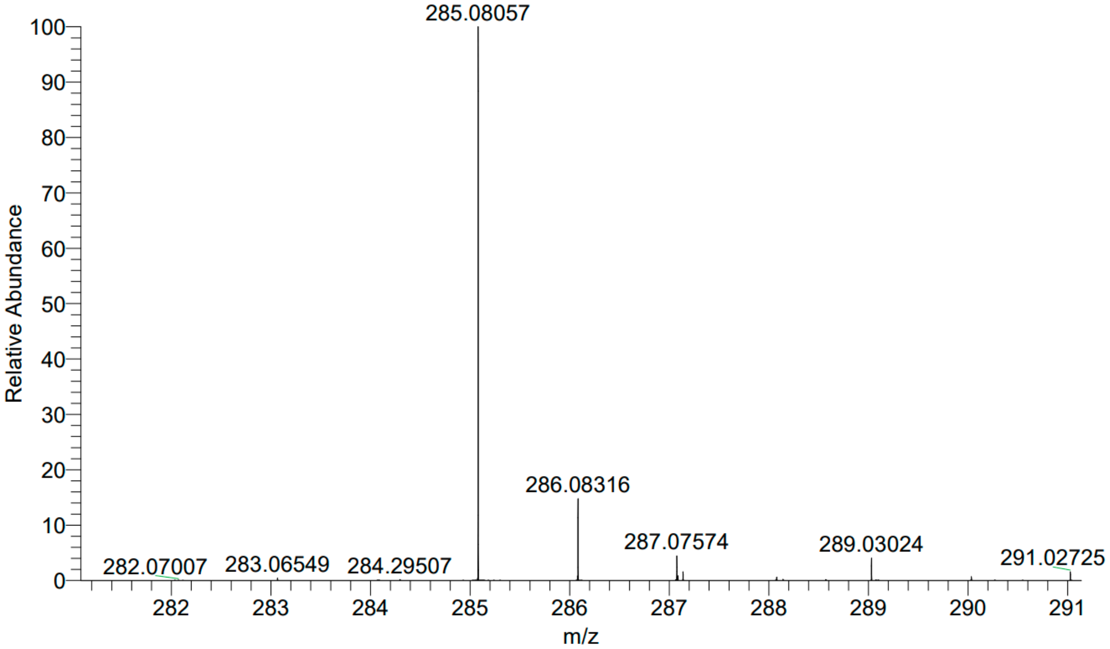

Figure S36 HRMS of target compound **I-10**

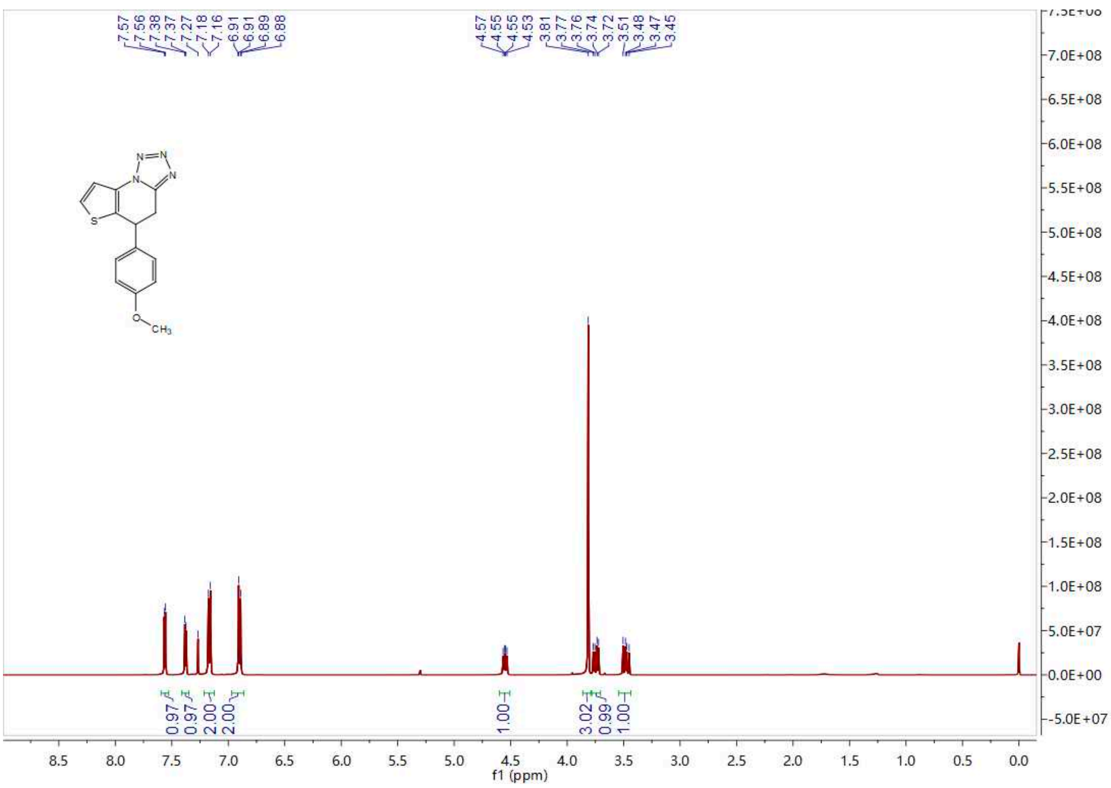

Figure S37 ^{1}HNMR of target compound **I-11**

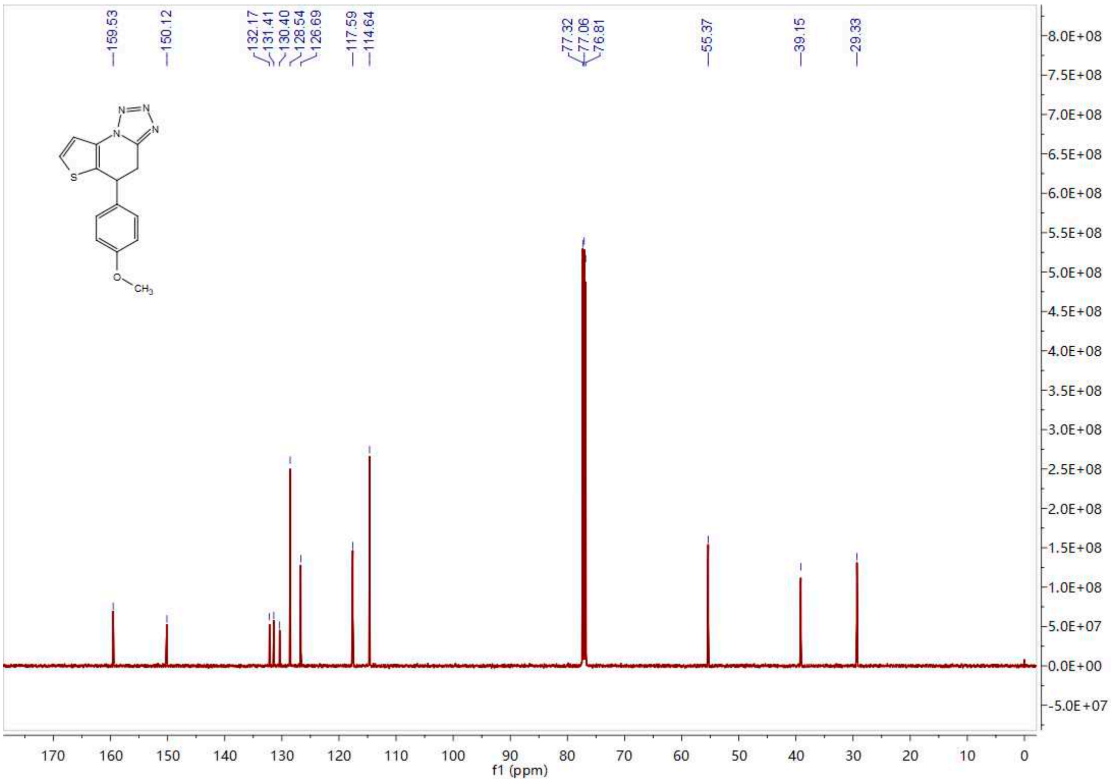

Figure S38 ^{13}CNMR of target compound **I-11**

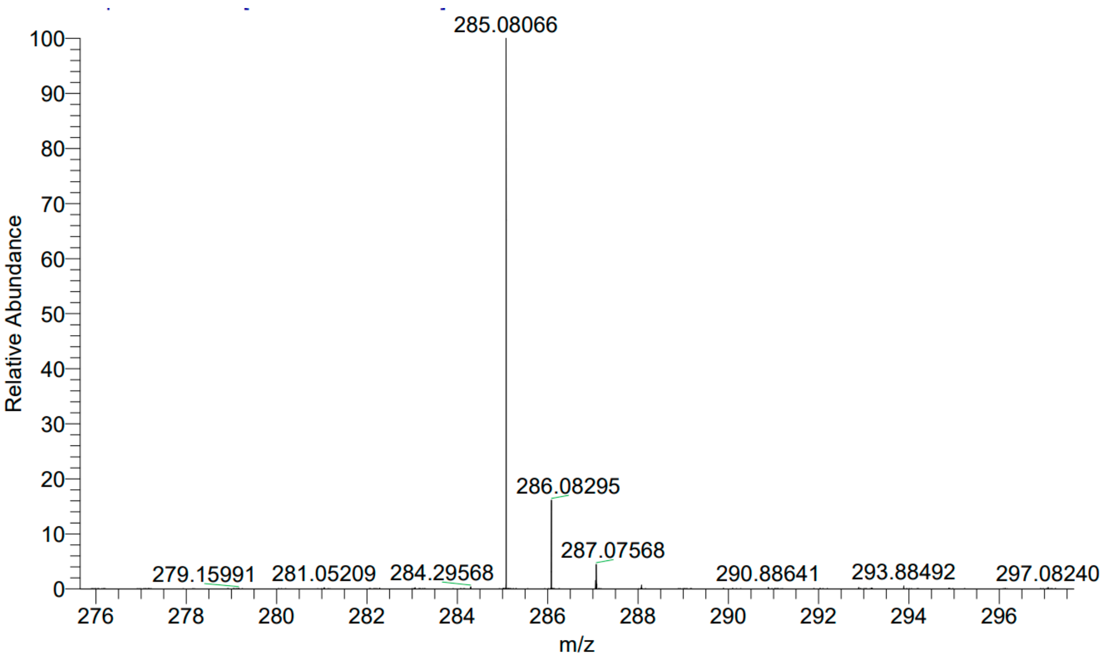

Figure S39 HRMS of target compound **I-11**

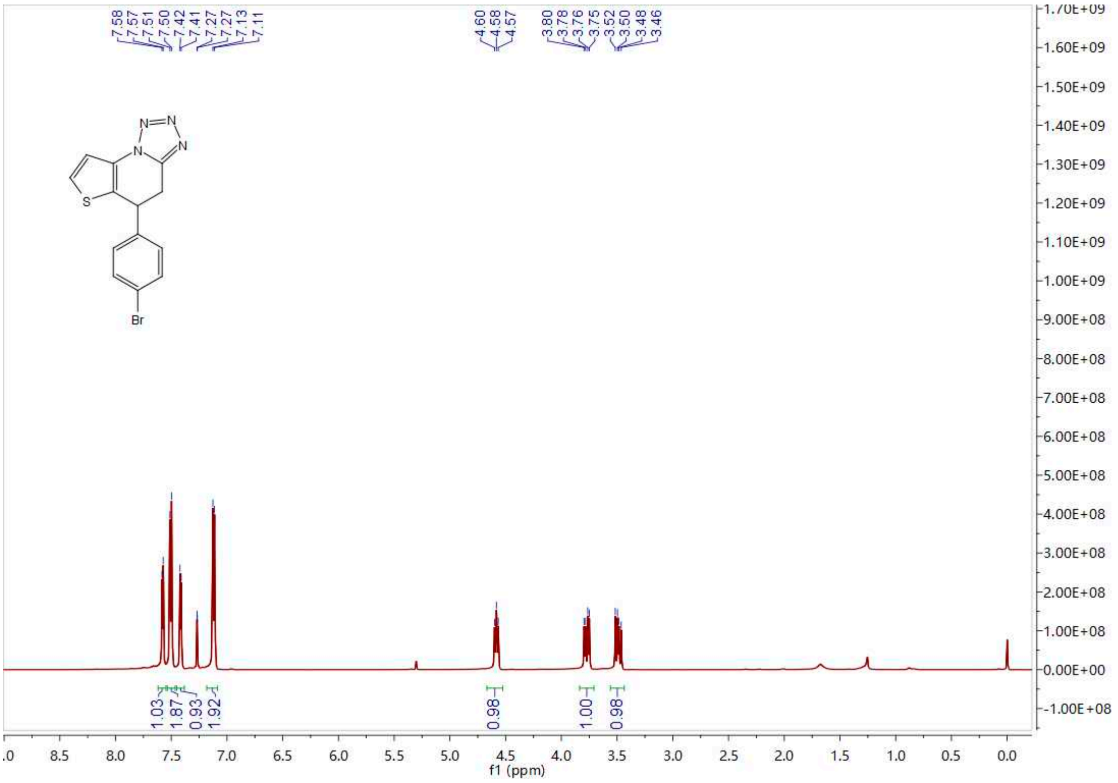

Figure S40 ^{1}HNMR of target compound **I-12**

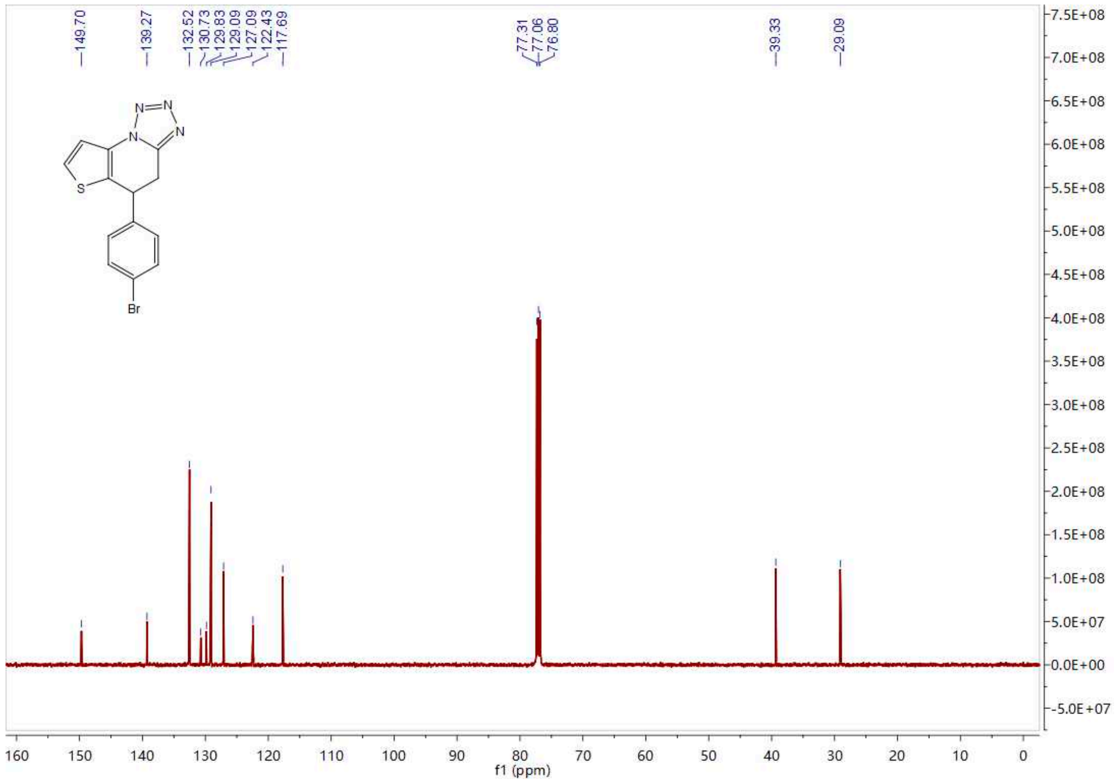

Figure S41 ^{13}CNMR of target compound **I-12**

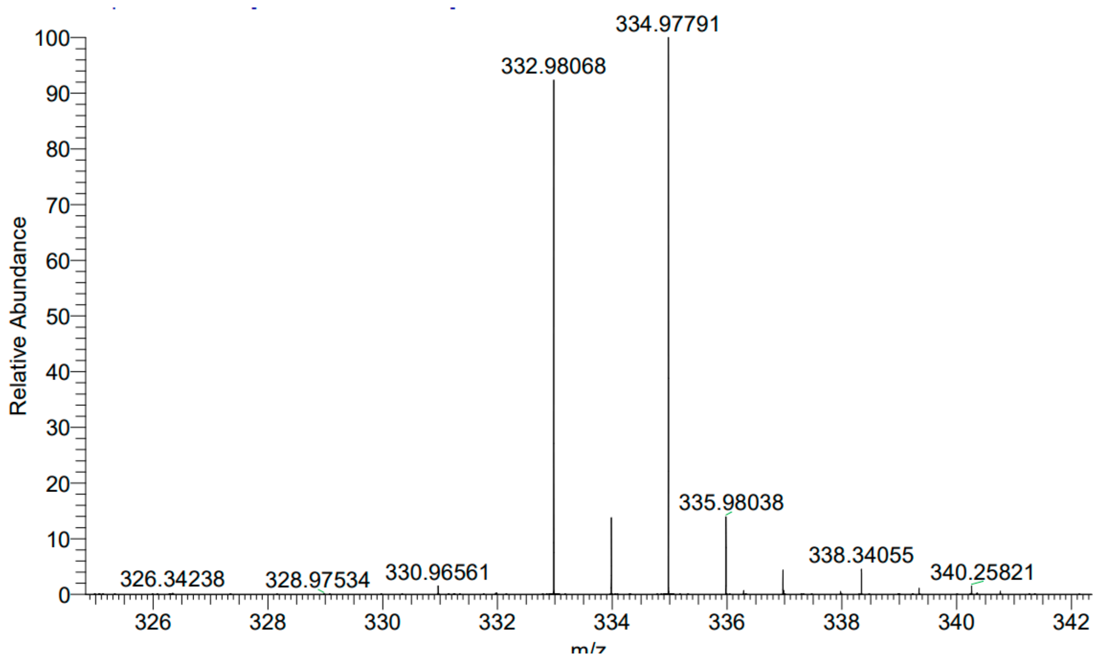

Figure S42 HRMS of target compound **I-12**

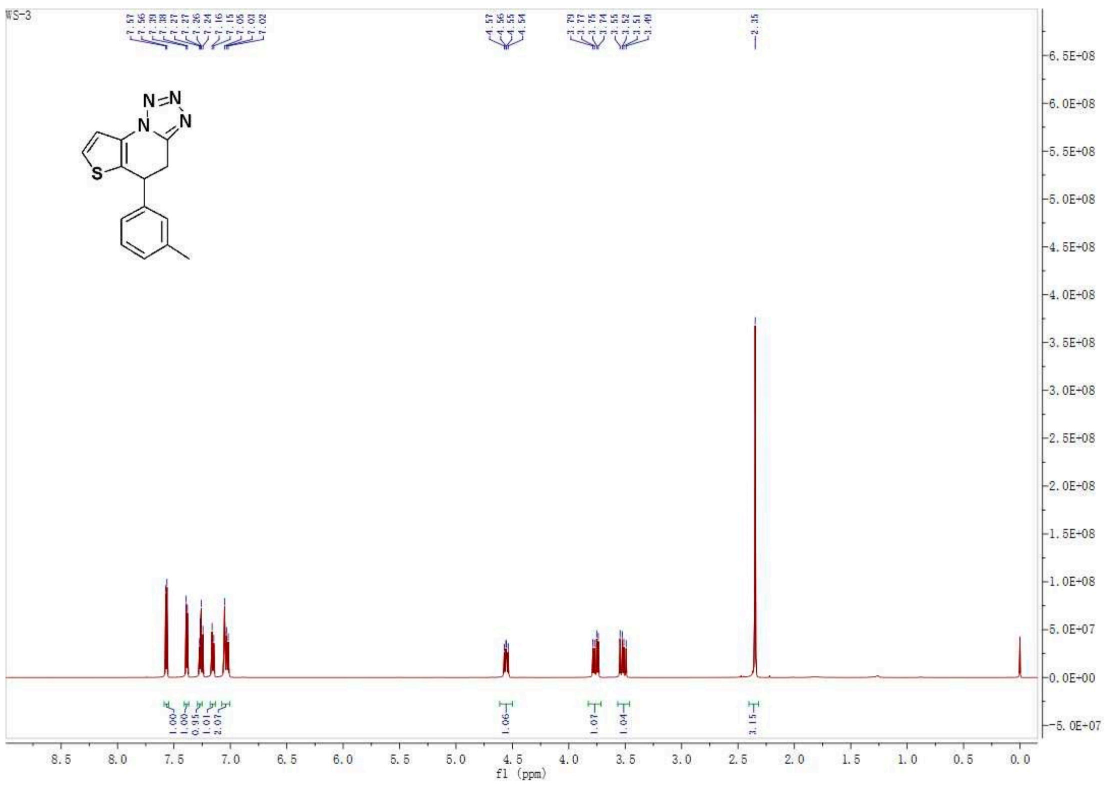

Figure S43 ^{1}HNMR of target compound **I-13**

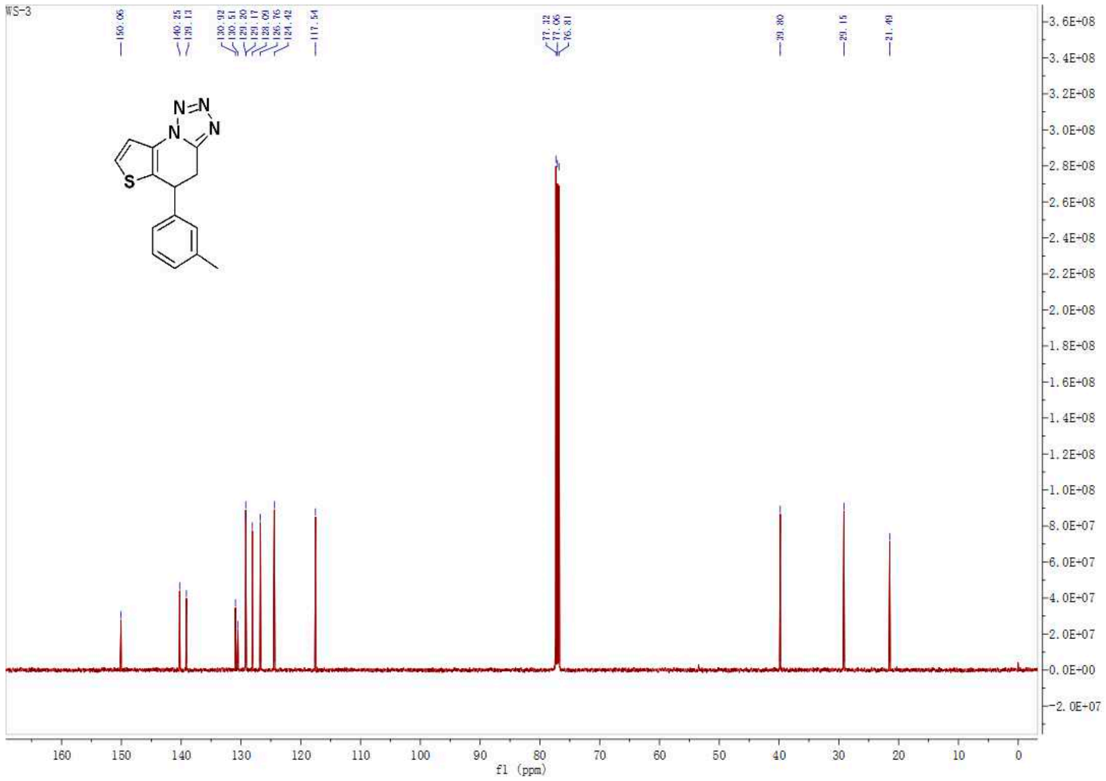

Figure S44 ^{13}CNMR of target compound **I-13**

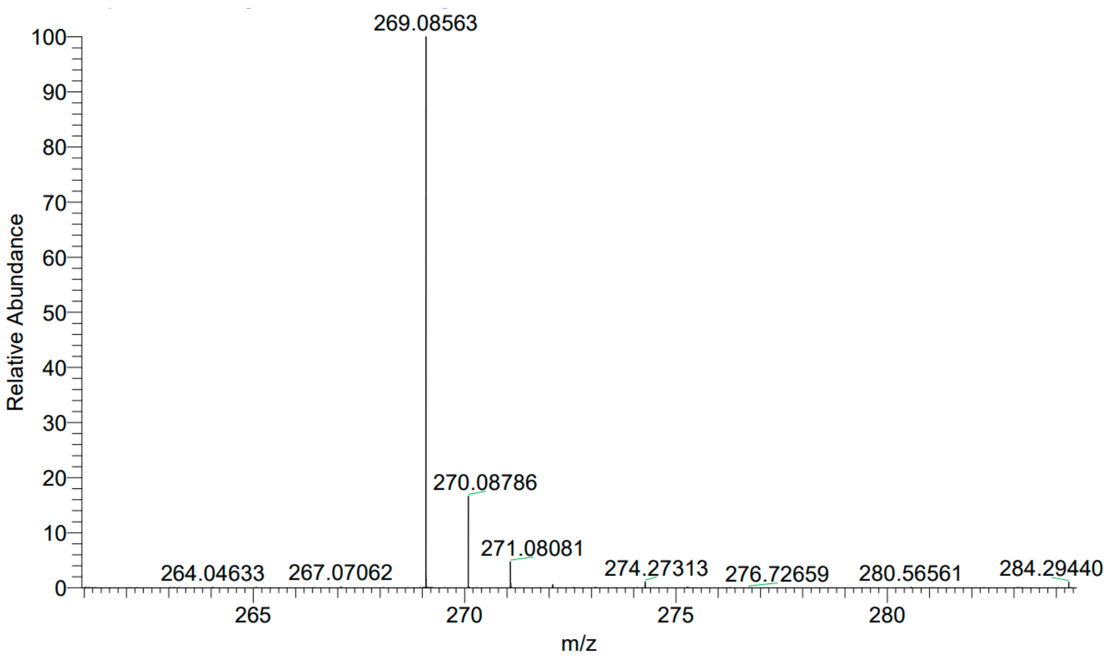

Figure S45 HRMS of target compound **I-13**

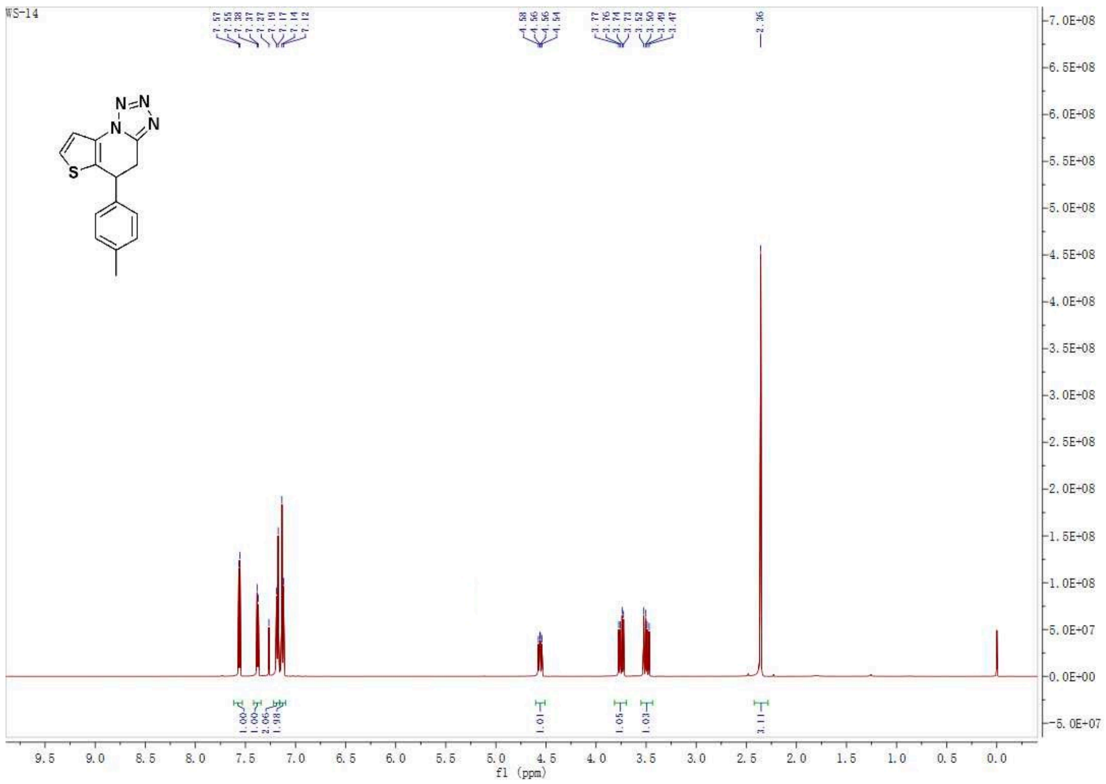

Figure S46 ^{1}HNMR of target compound **I-14**

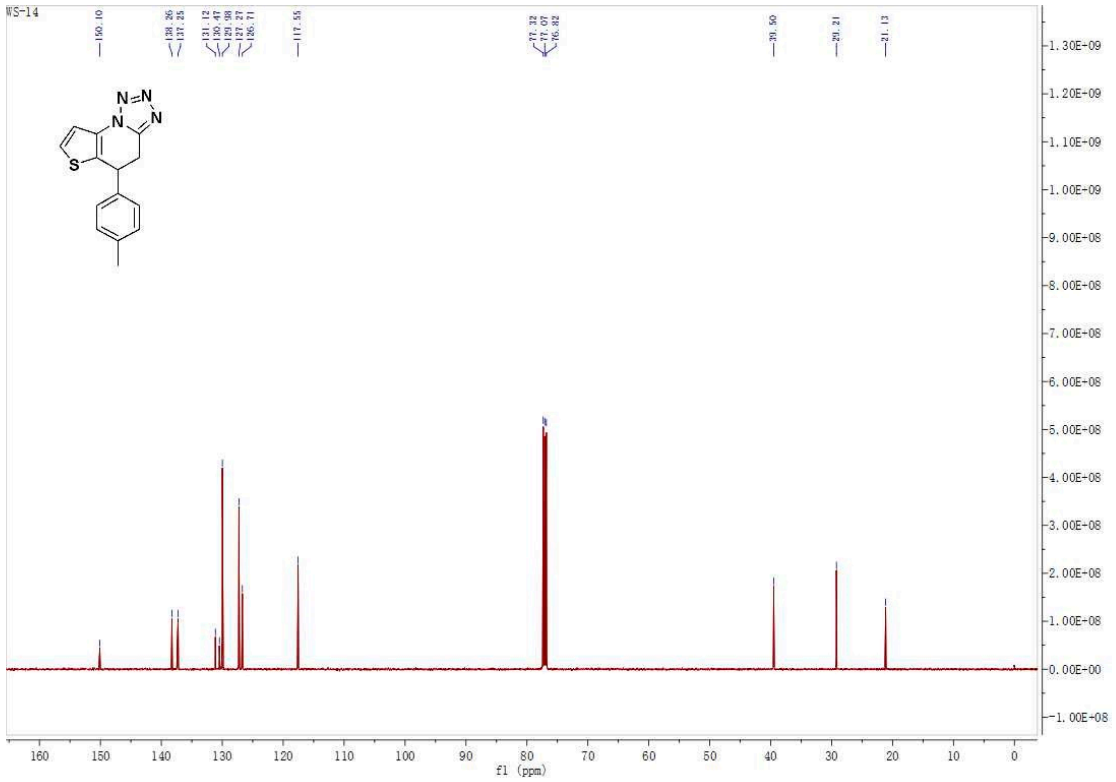

Figure S47 ^{13}CNMR of target compound **I-14**

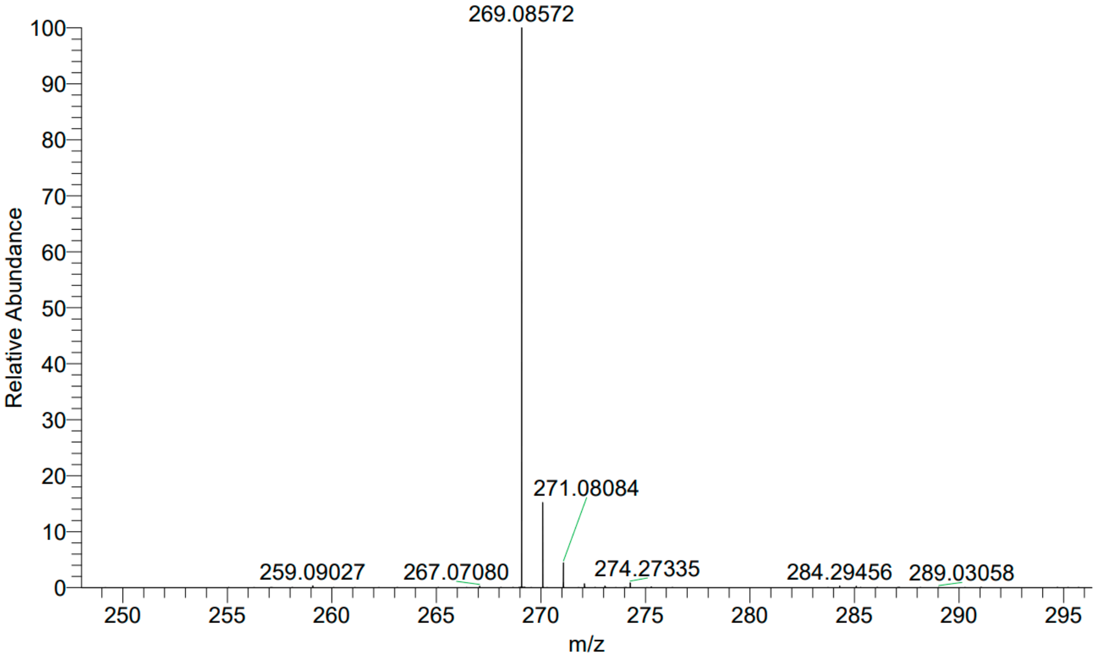

Figure S48 HRMS of target compound **I-14**

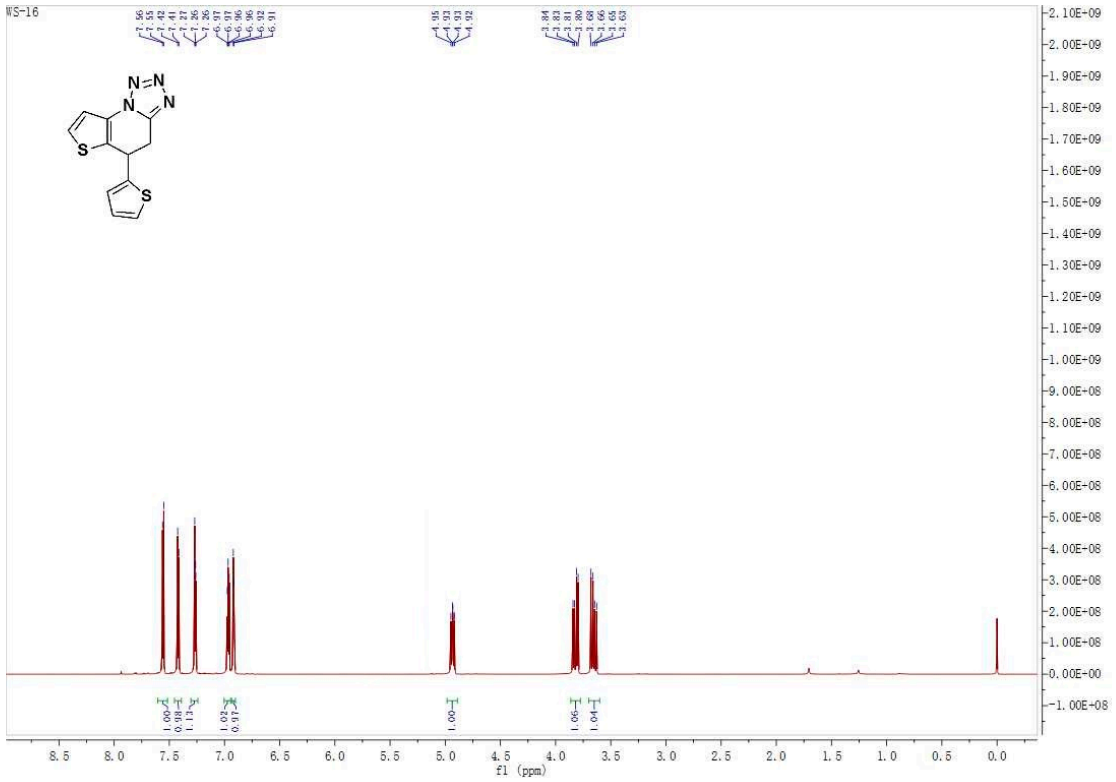

Figure S49 ^{1}HNMR of target compound **I-15**

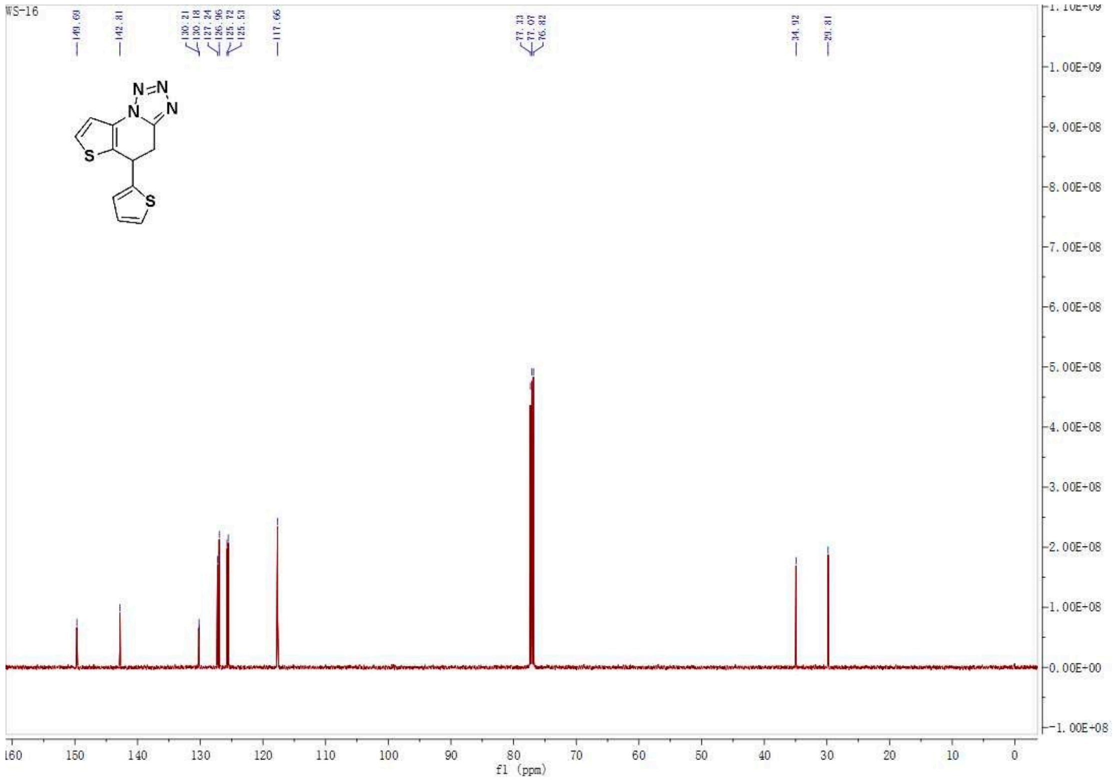

Figure S50 ^{13}CNMR of target compound **I-15**

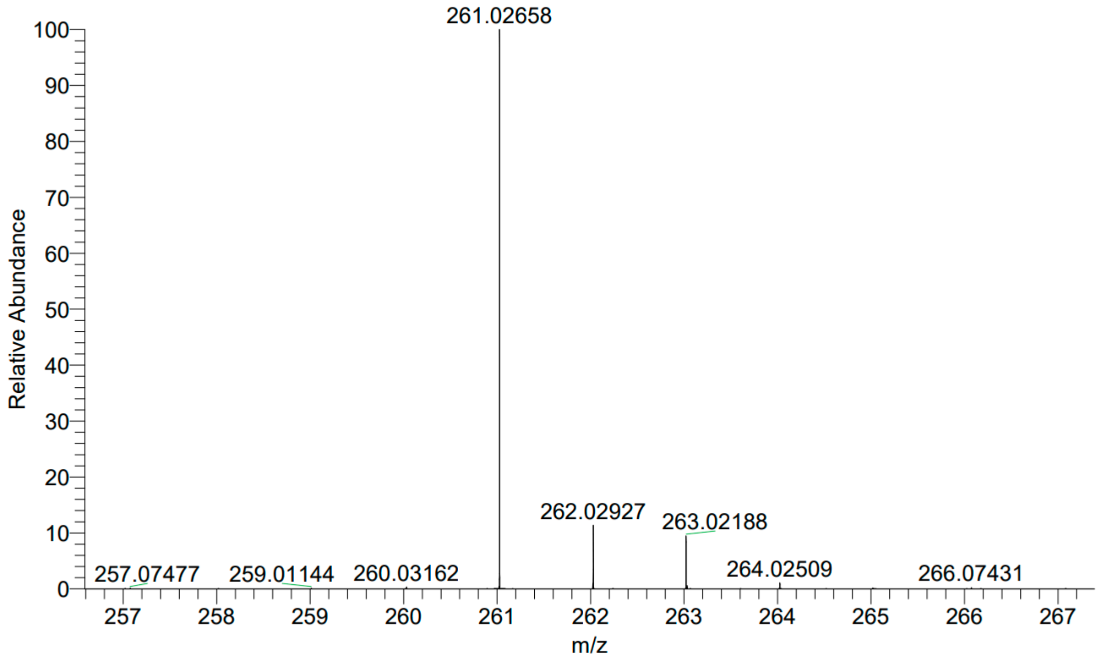

Figure S51 HRMS of target compound **I-15**

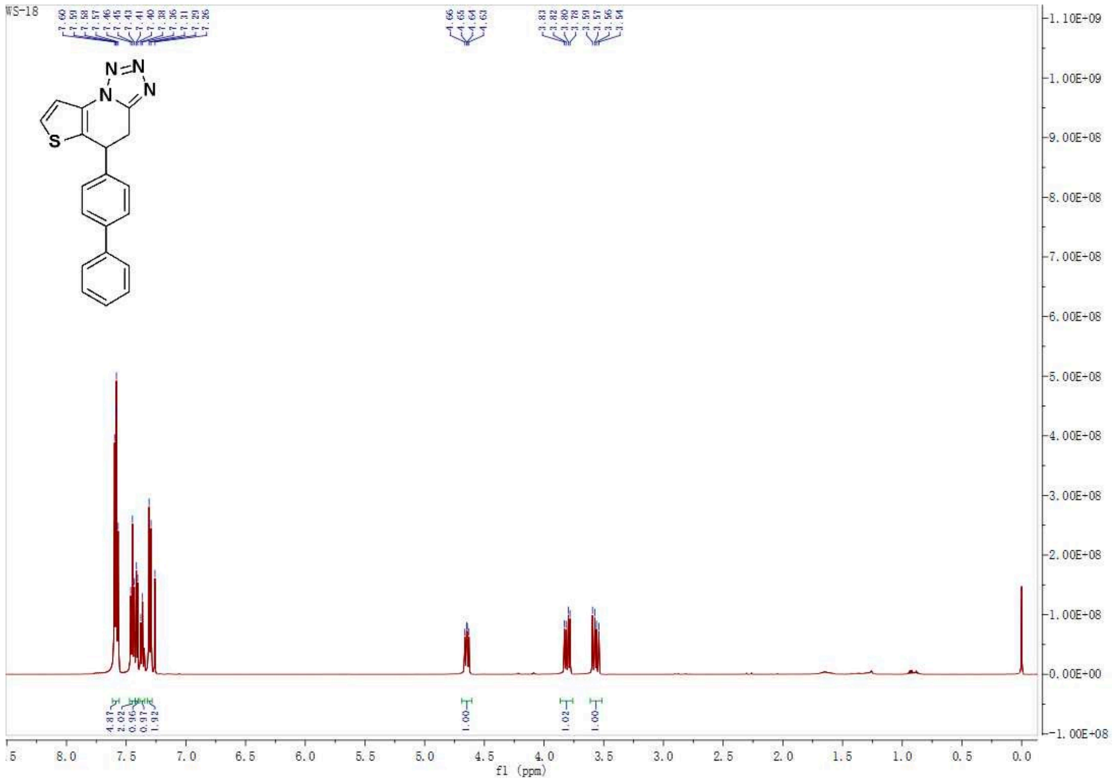

Figure S52 ^{1}HNMR of target compound **I-16**

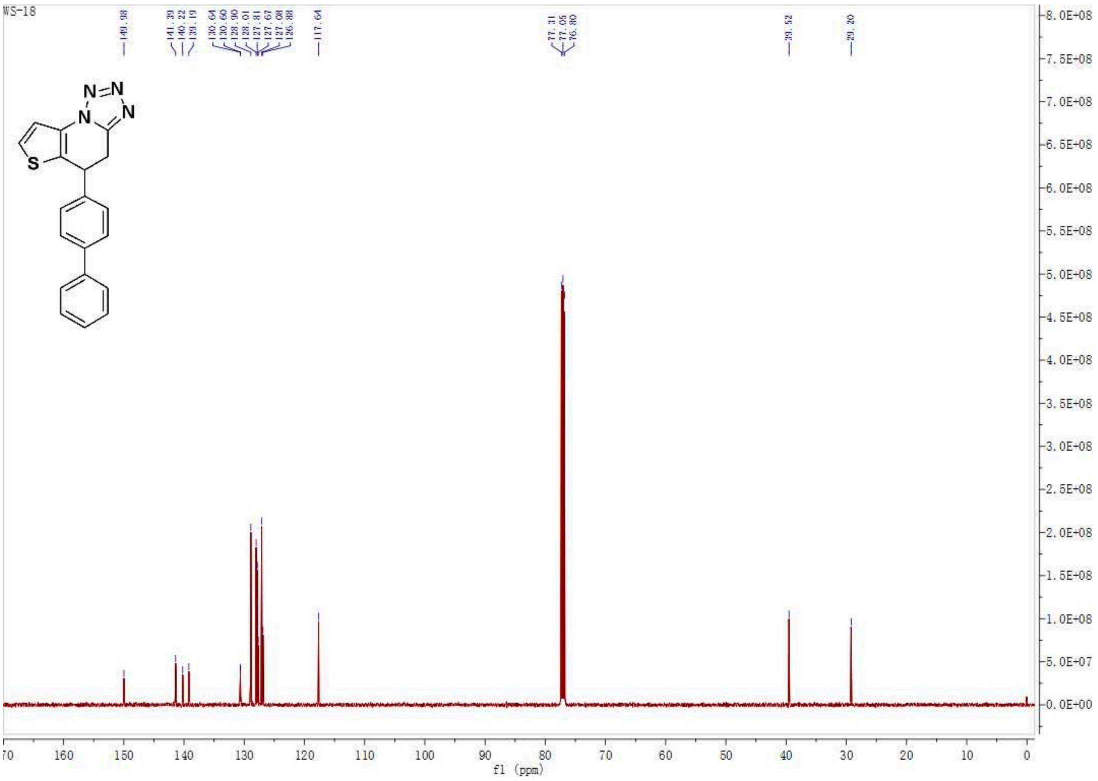

Figure S53 ^{13}CNMR of target compound **I-16**

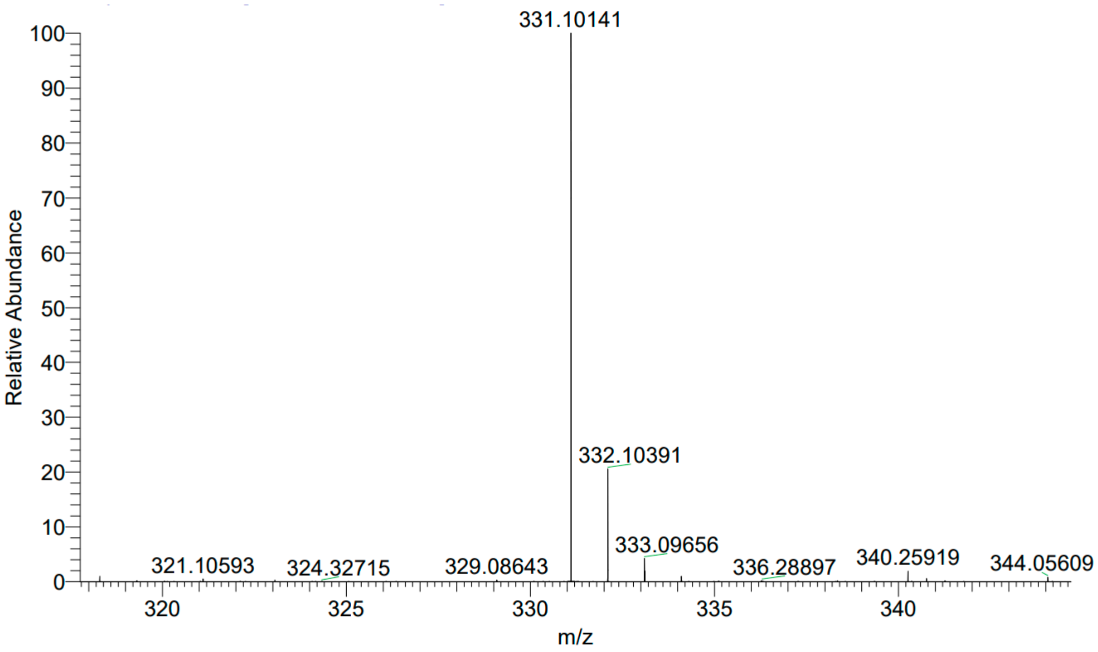

Figure S54 HRMS of target compound **I-16**

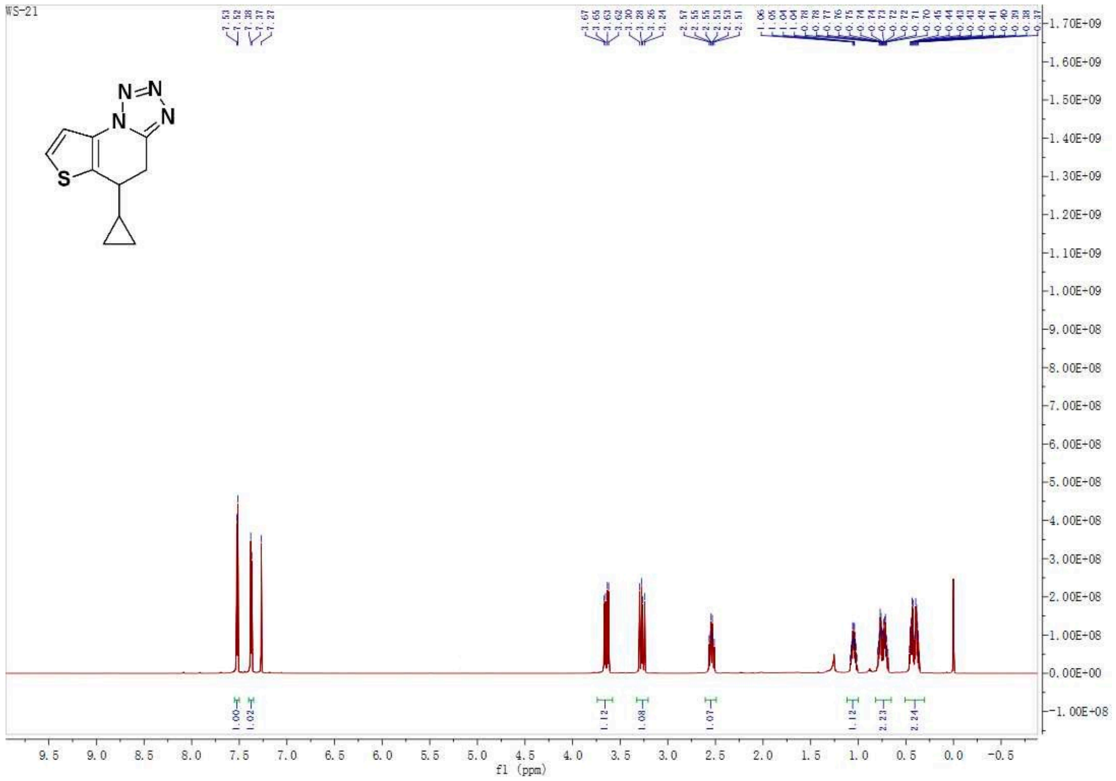

Figure S55 ^{1}HNMR of target compound **I-17**

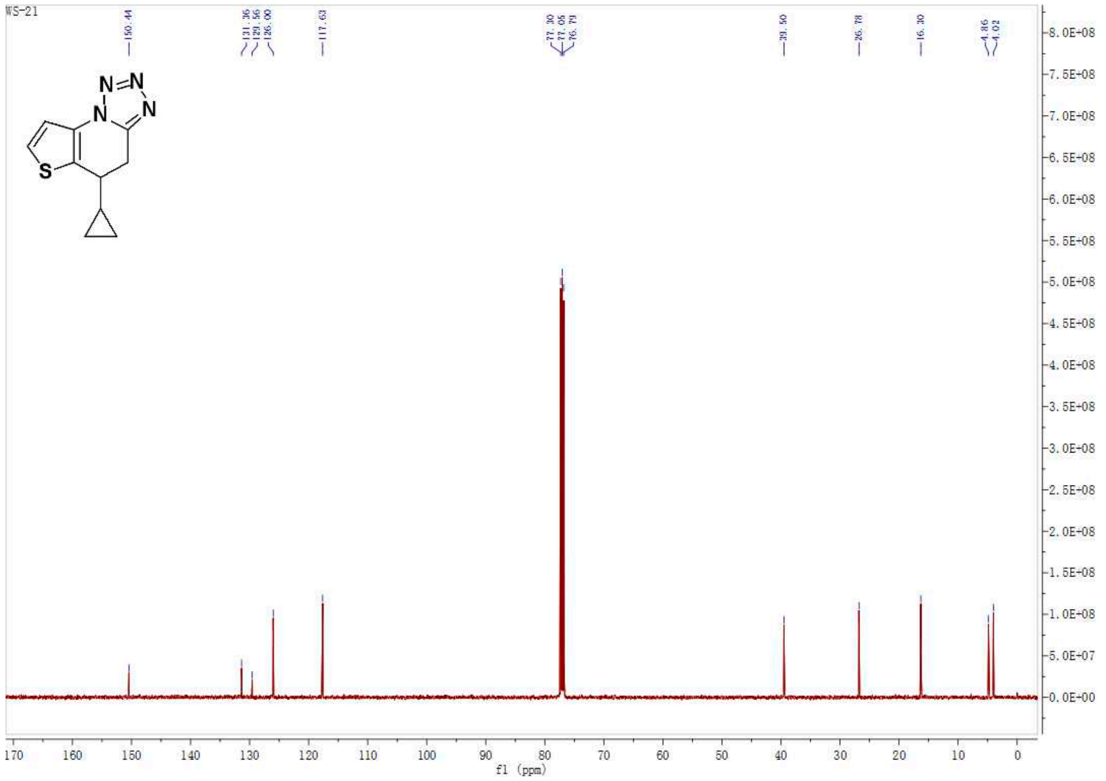

Figure S56 ^{13}CNMR of target compound **I-17**

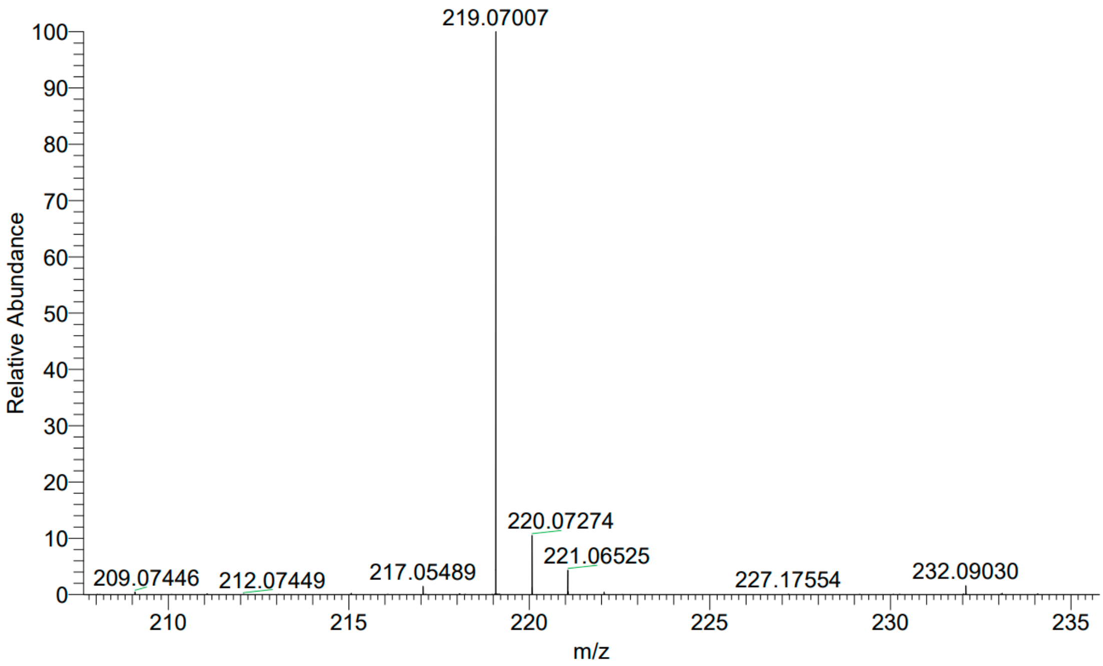

Figure S57 HRMS of target compound **I-17**

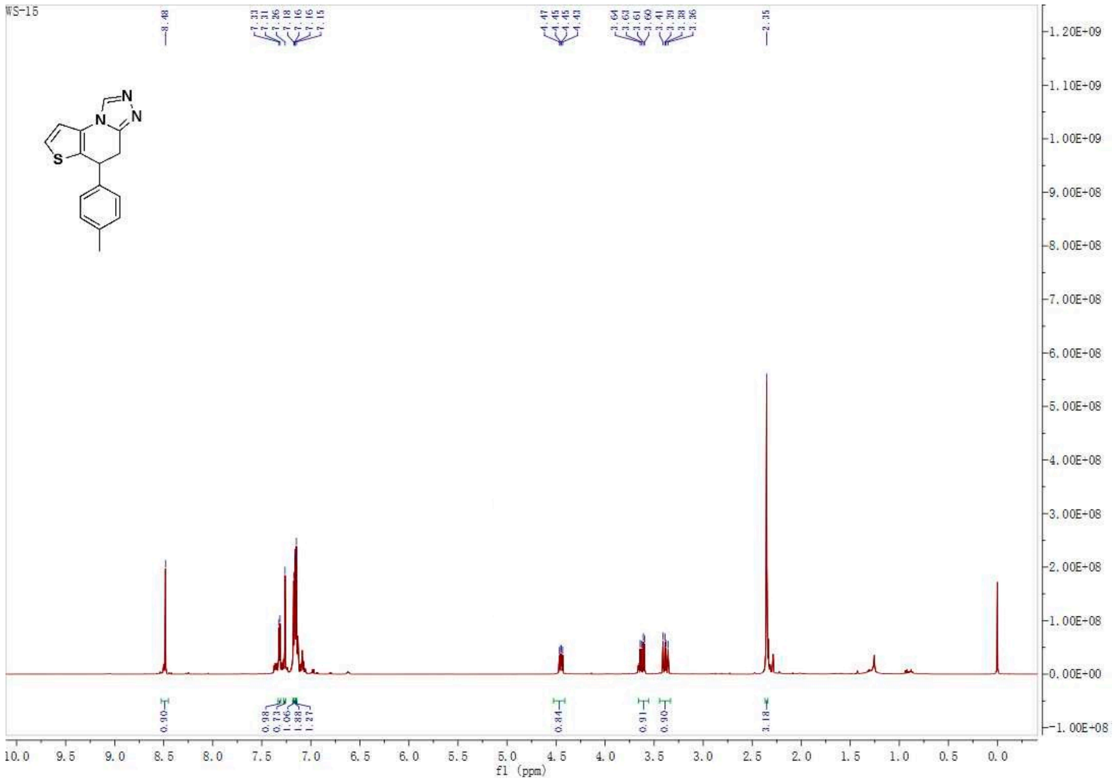

Figure S58 ^{1}HNMR of target compound **II-1**

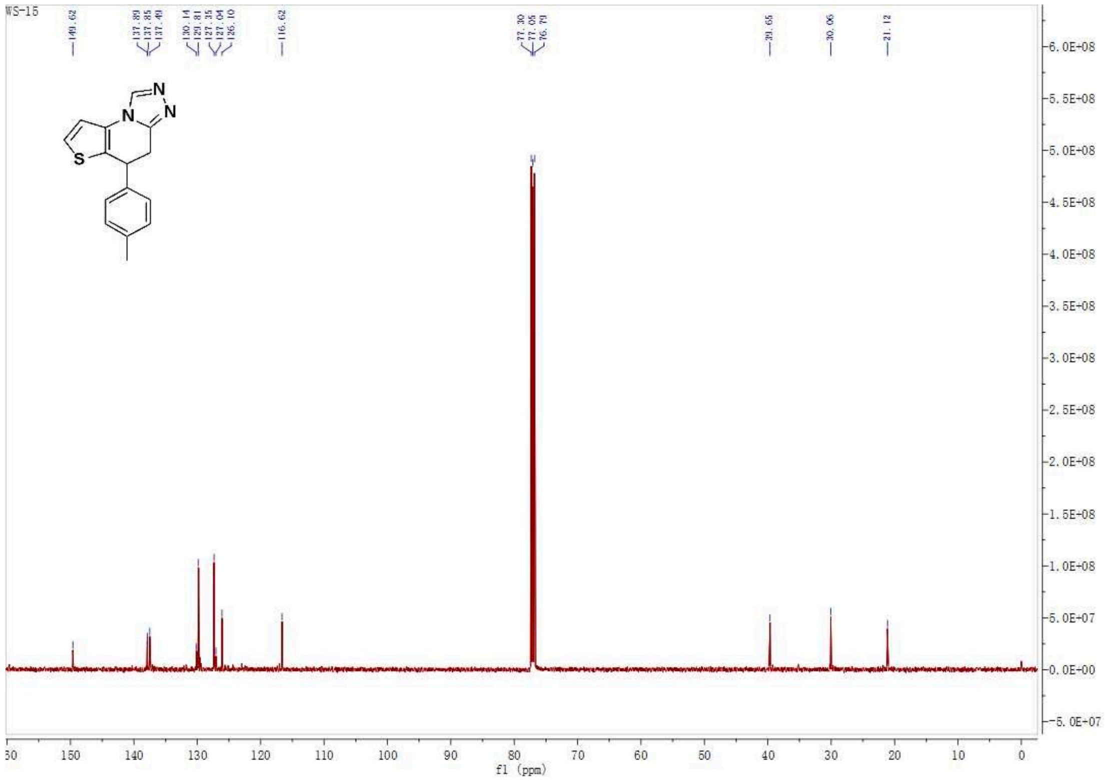

Figure S59 ^{13}CNMR of target compound **II-1**

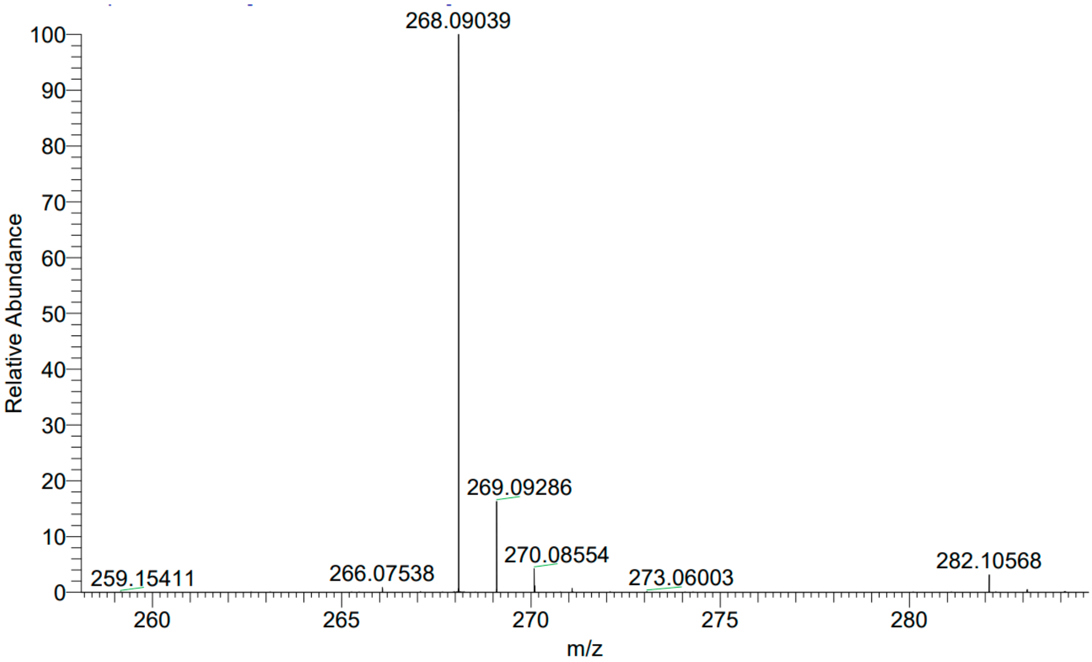

Figure S60 HRMS of target compound **II-1**

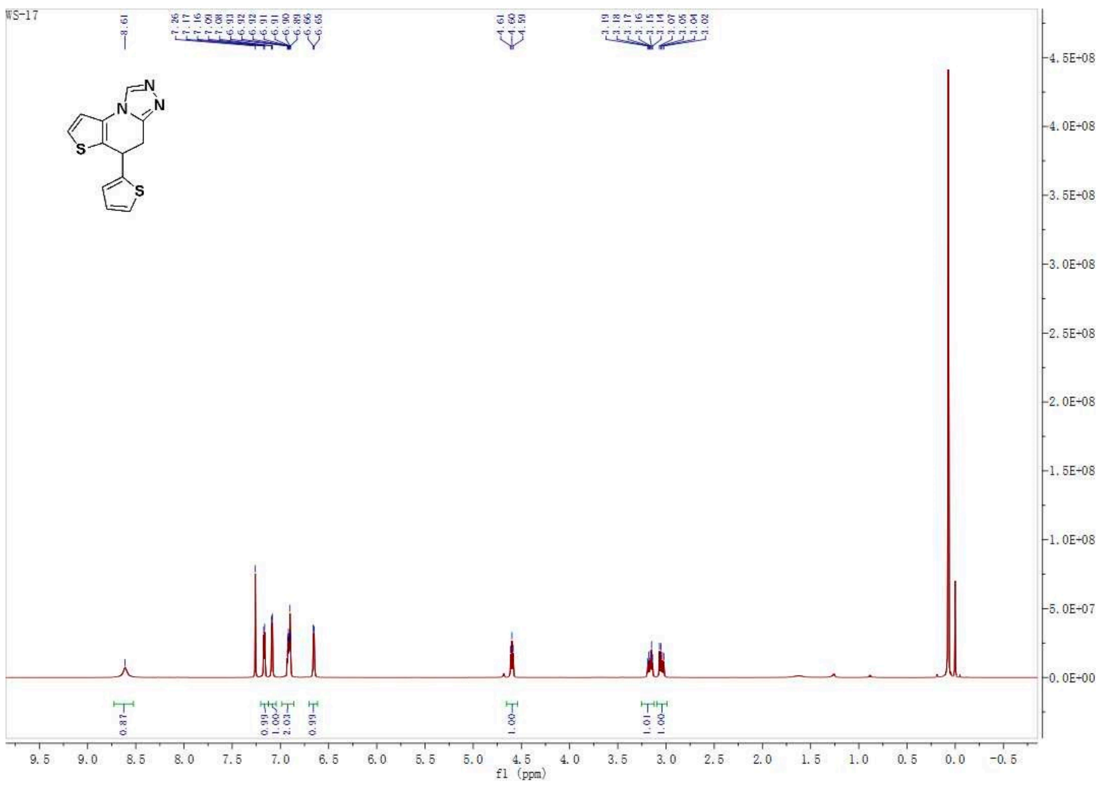

Figure S61 ^{1}HNMR of target compound **II-2**

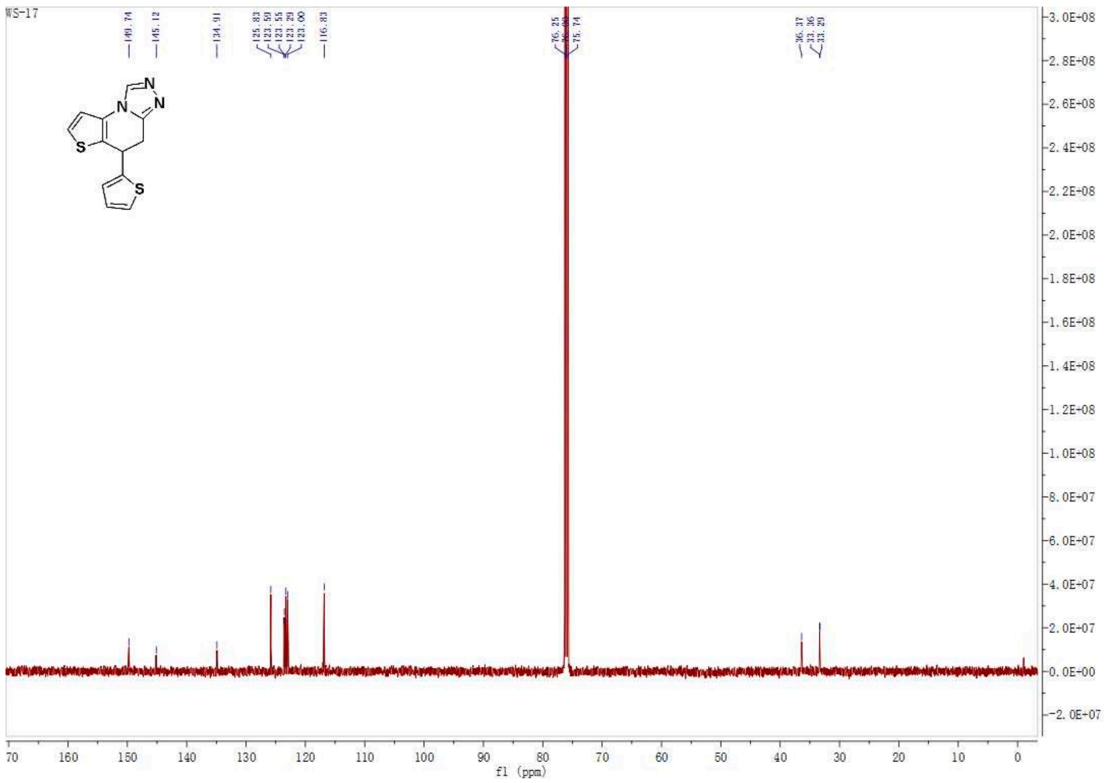

Figure S62 ^{13}CNMR of target compound **II-2**

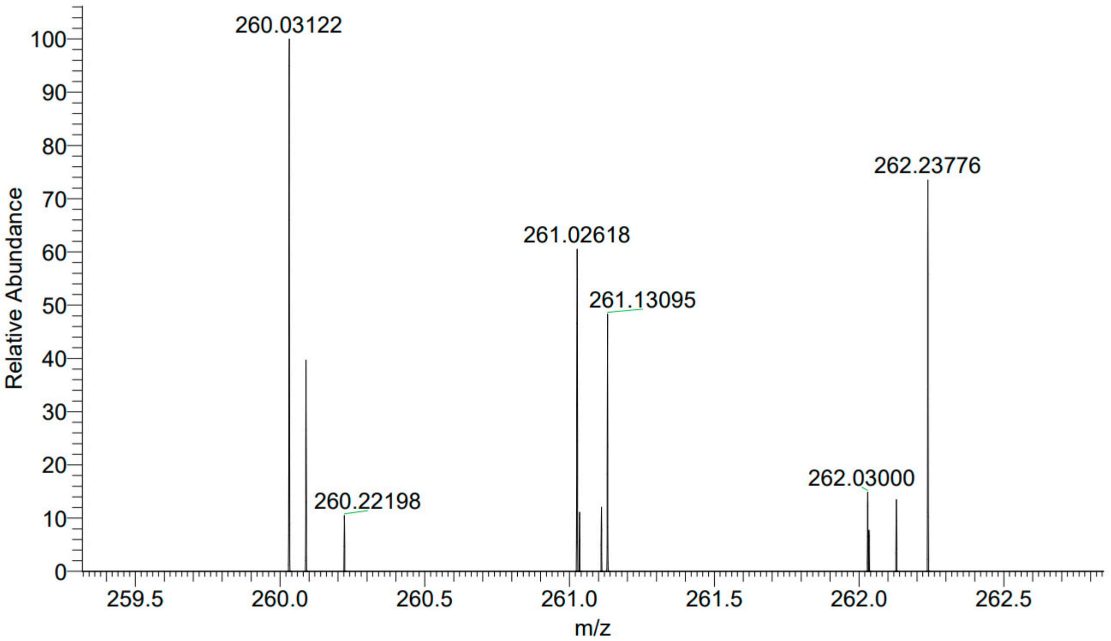

Figure S63 HRMS of target compound **II-2**

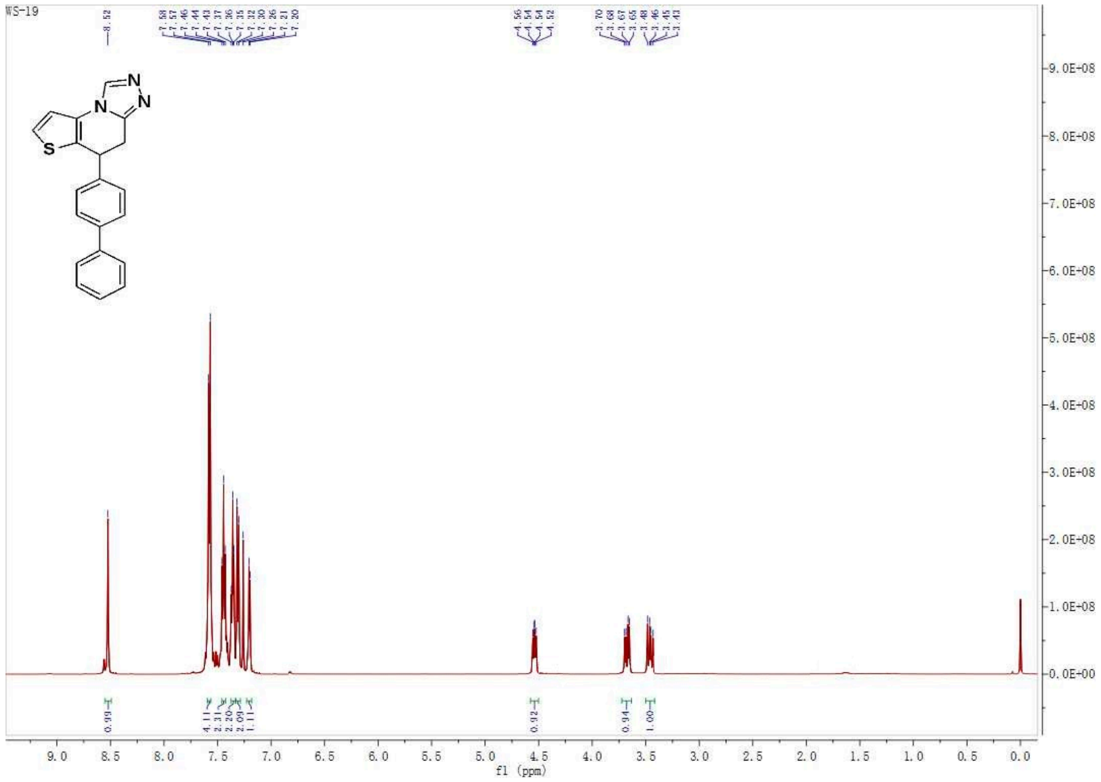

Figure S64 ^{1}HNMR of target compound **II-3**

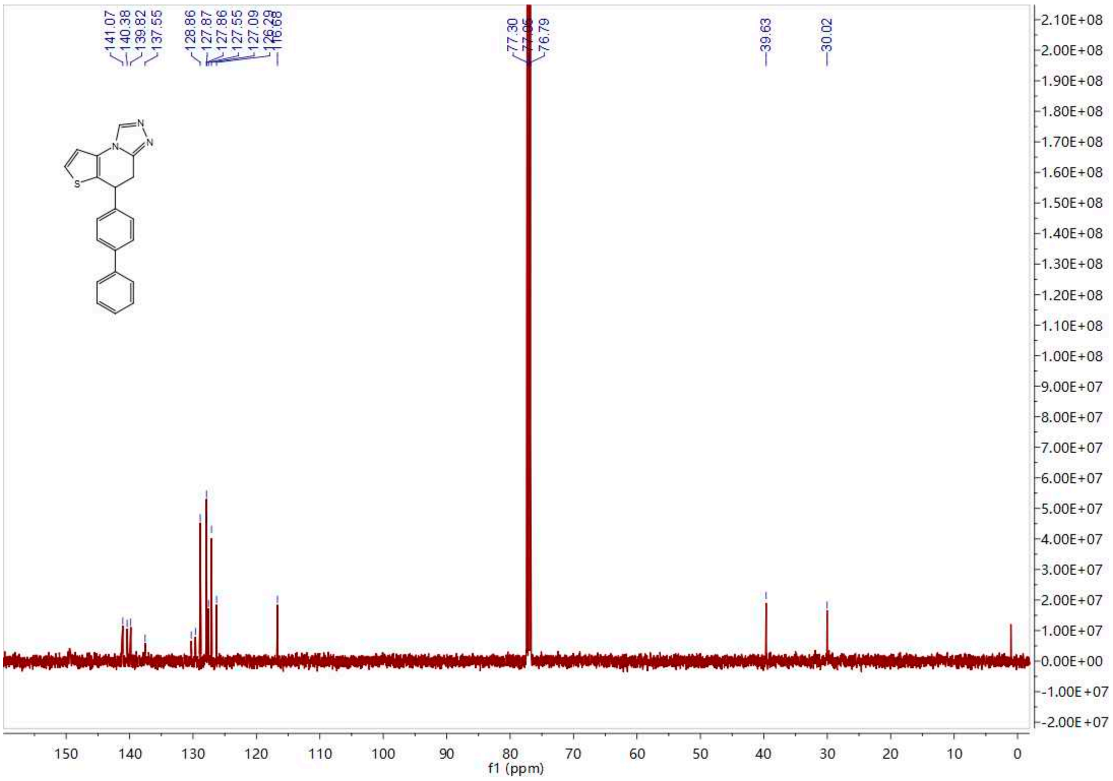

Figure S65 ^{13}CNMR of target compound **II-3**

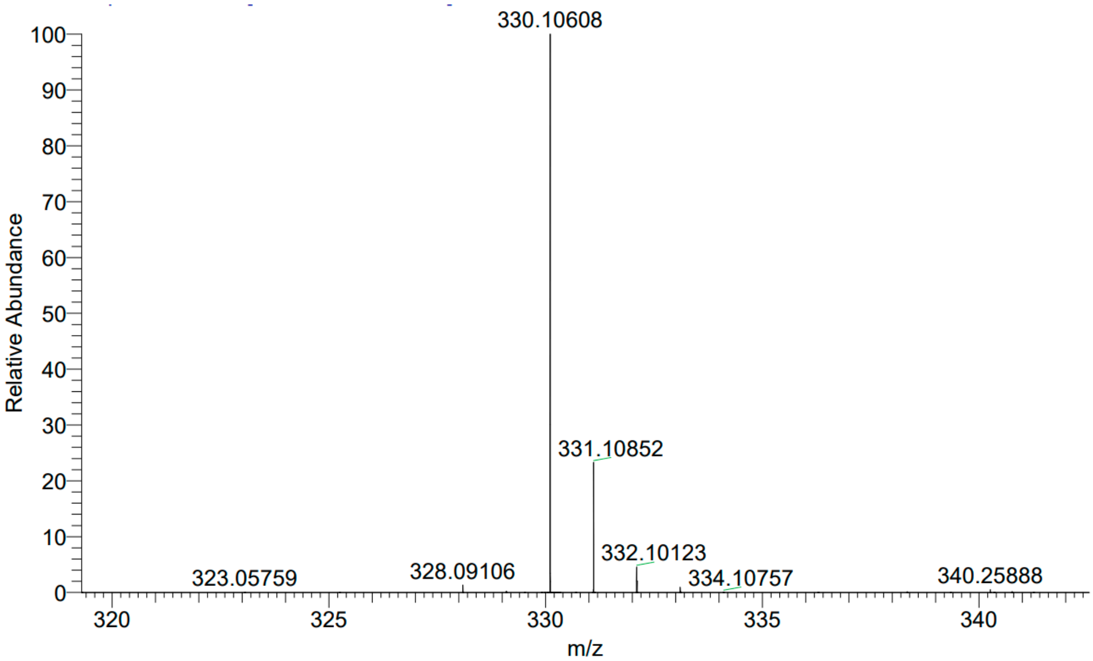

Figure S66 HRMS of target compound **II-3**

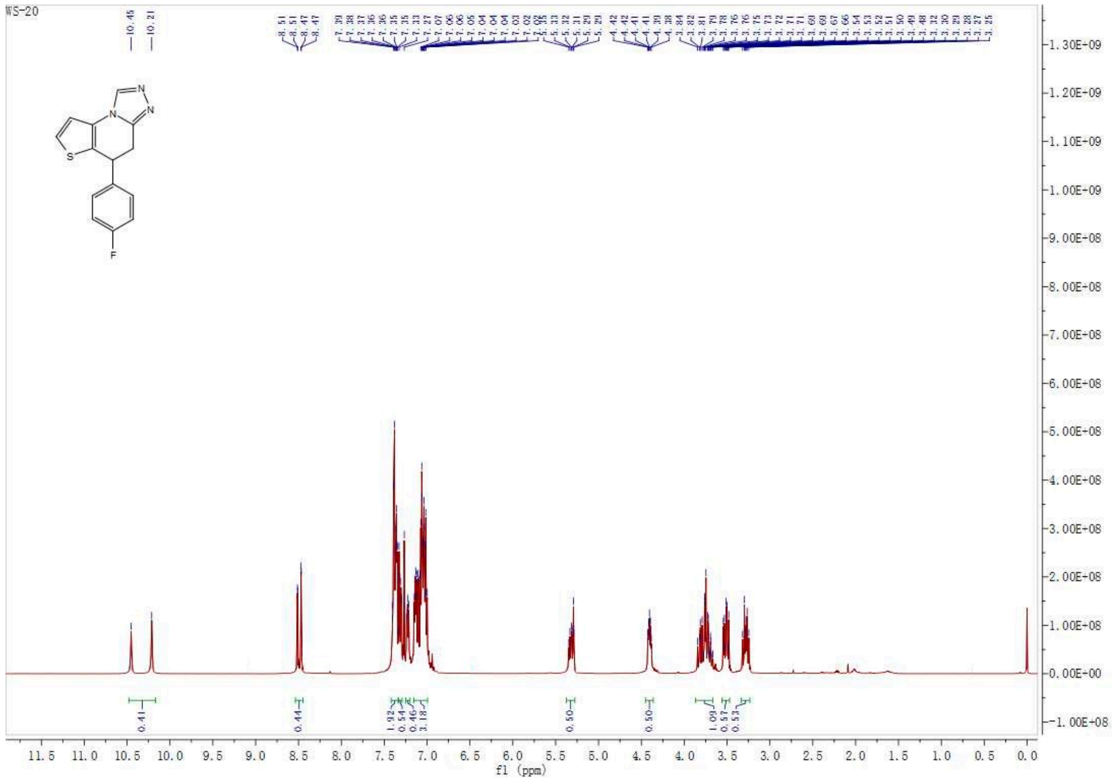

Figure S67 ^{1}HNMR of target compound **II-4**

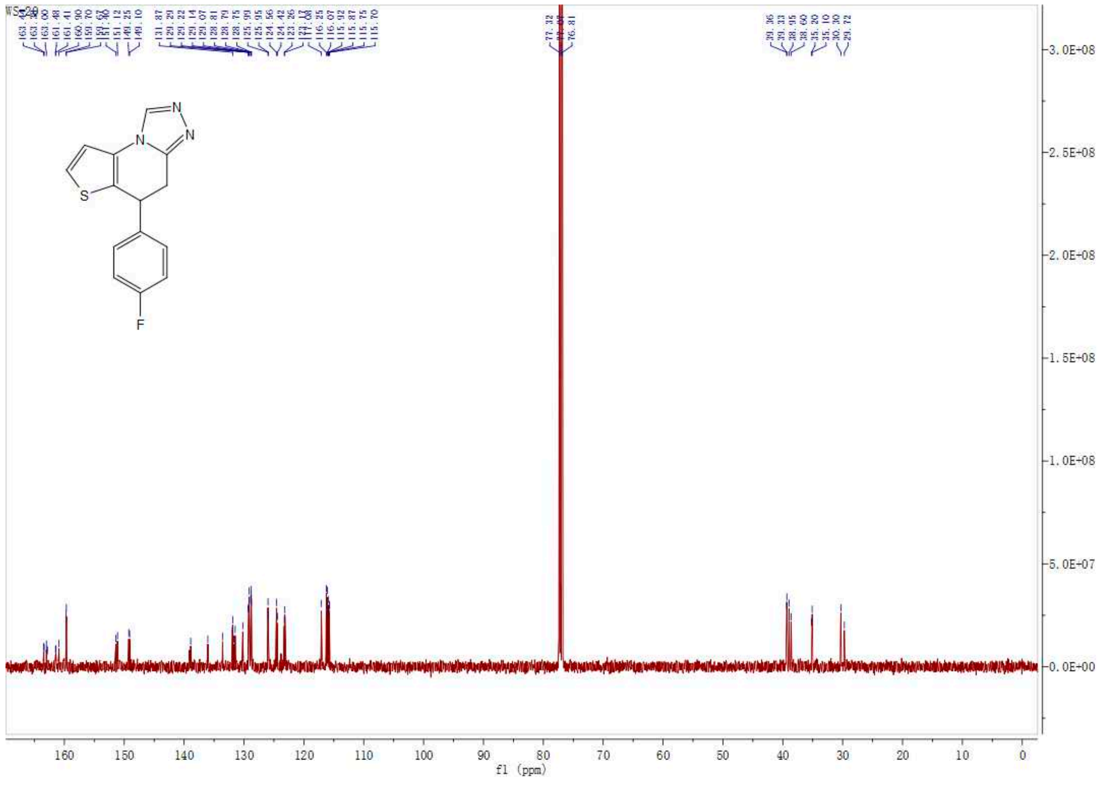

Figure S68 ^{13}CNMR of target compound **II-4**

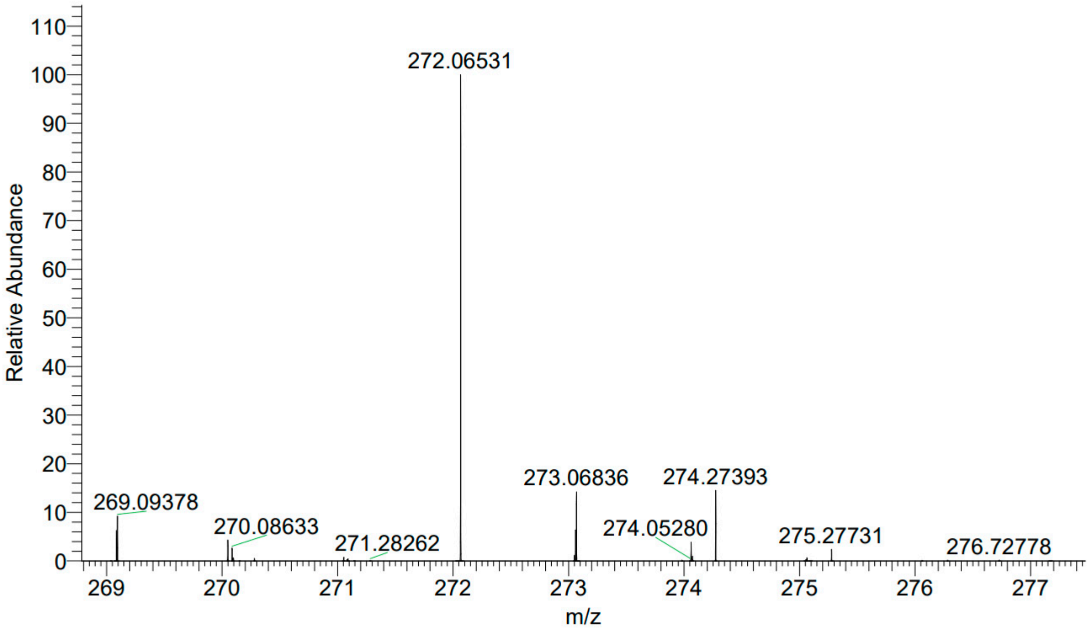

Figure S69 HRMS of target compound **II-4**

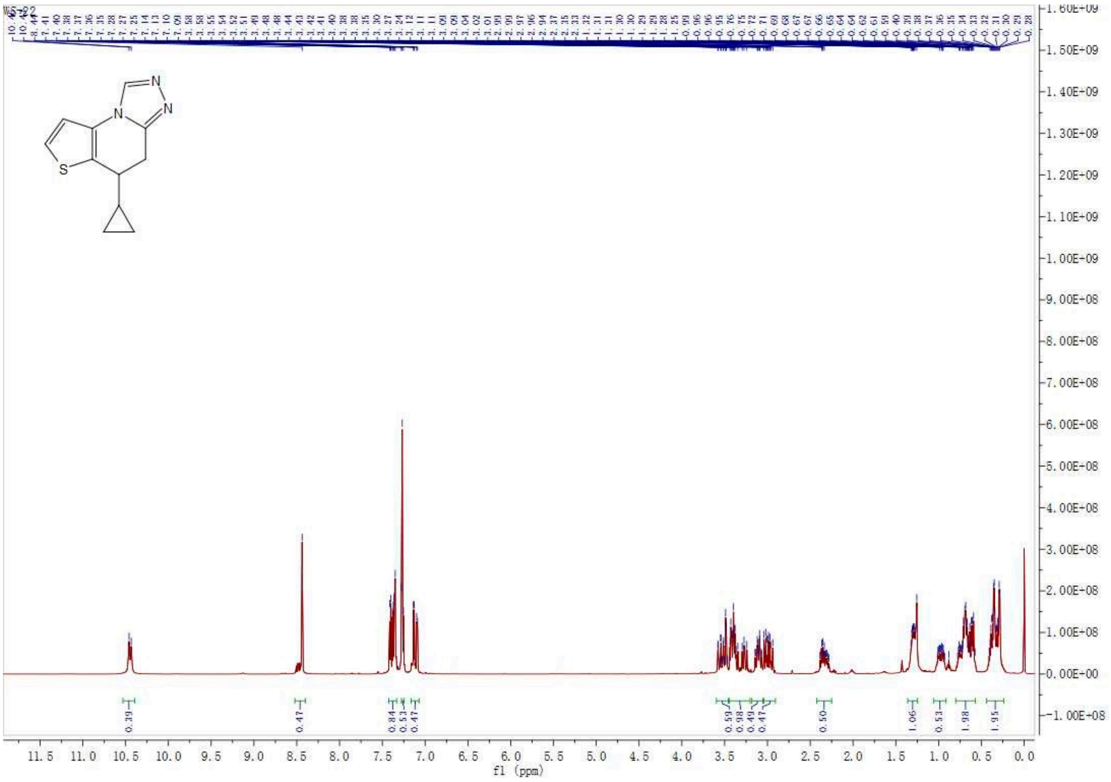

Figure S70 ^{1}HNMR of target compound **II-5**

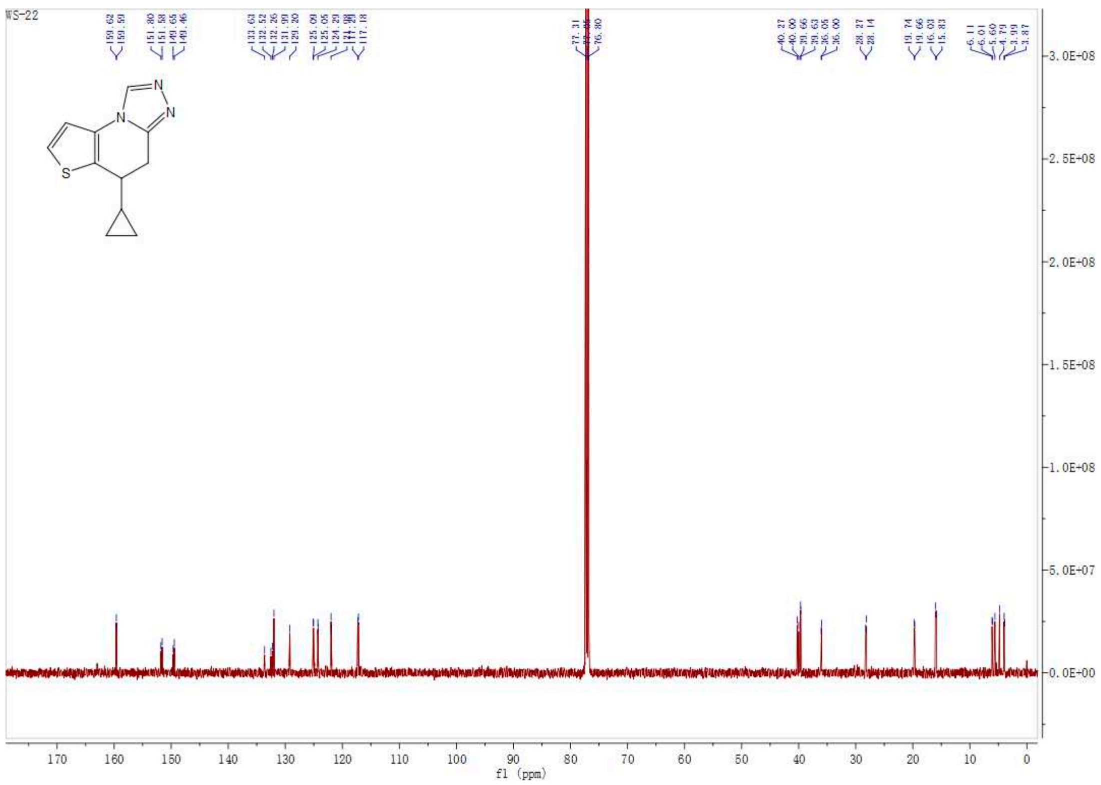

Figure S71 ^{13}CNMR of target compound **II-5**

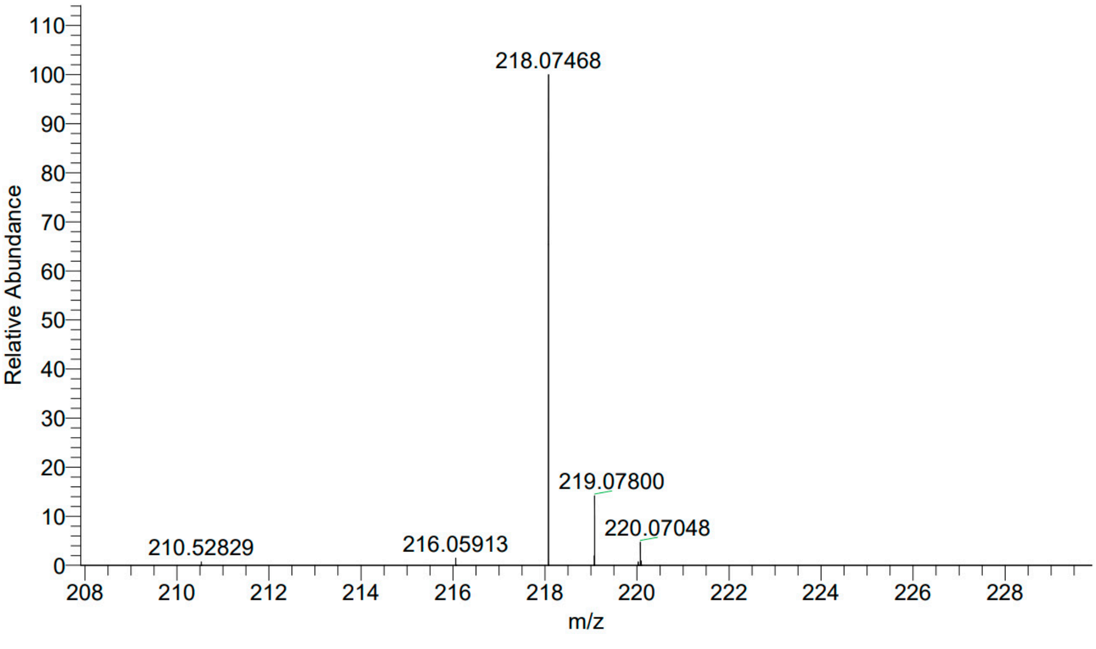

Figure S72 HRMS of target compound **II-5**

3. KEGG pathway analysis of DEGs.

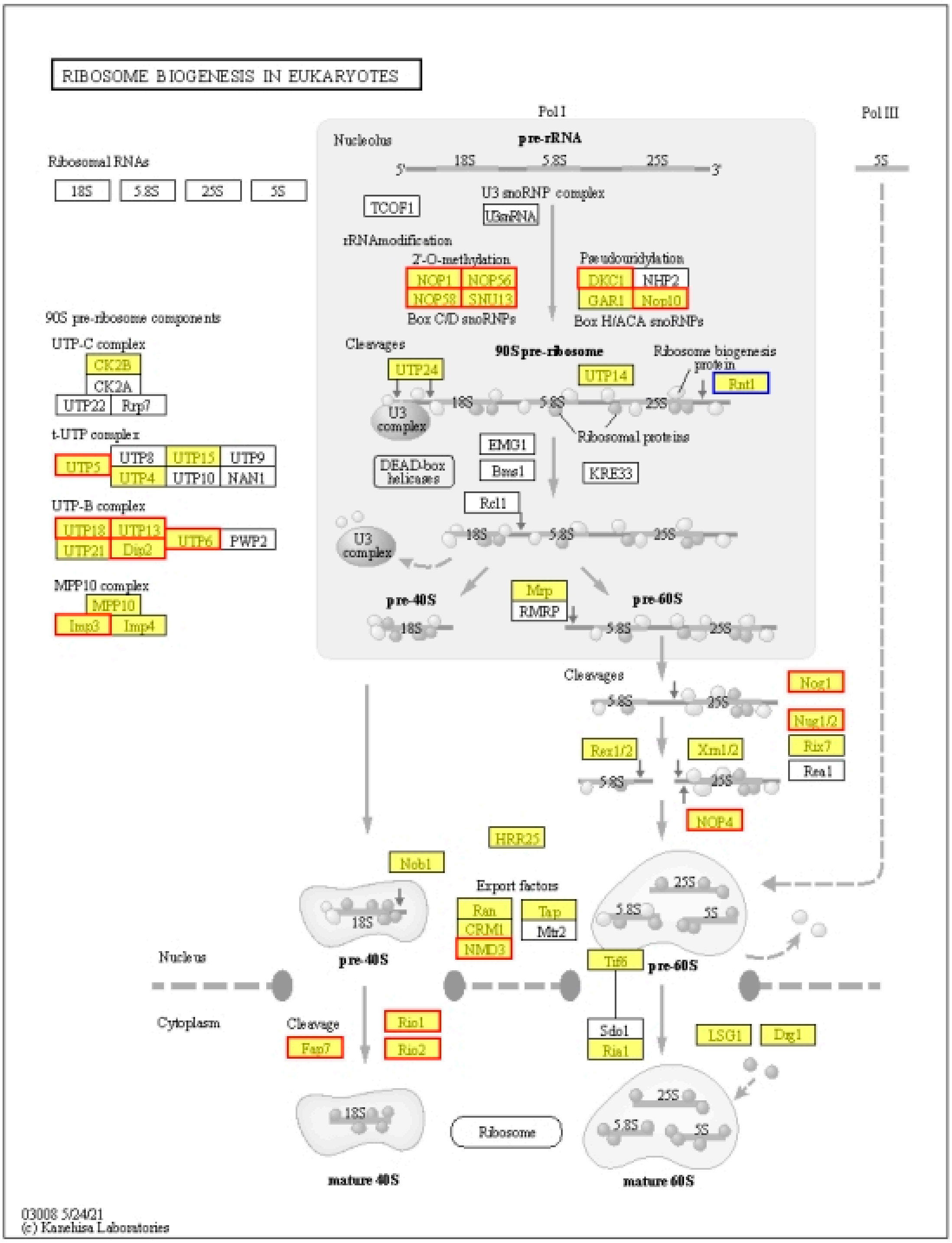

Figure S73 Ribosome KEGG pathway analysis of DEGs.

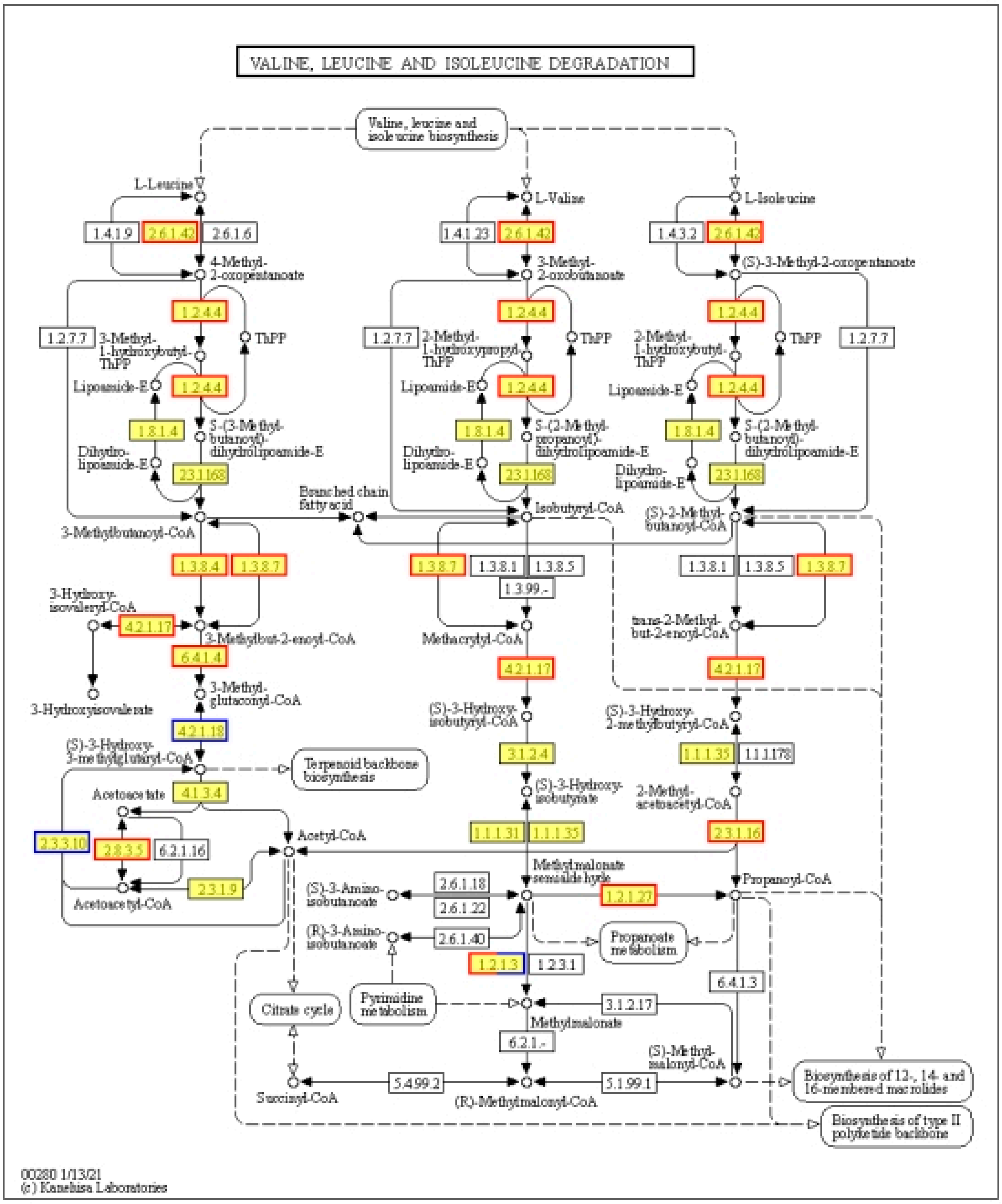

Figure S74 Valine leucine and isoleucine degradation KEGG pathway analysis of DEGs.

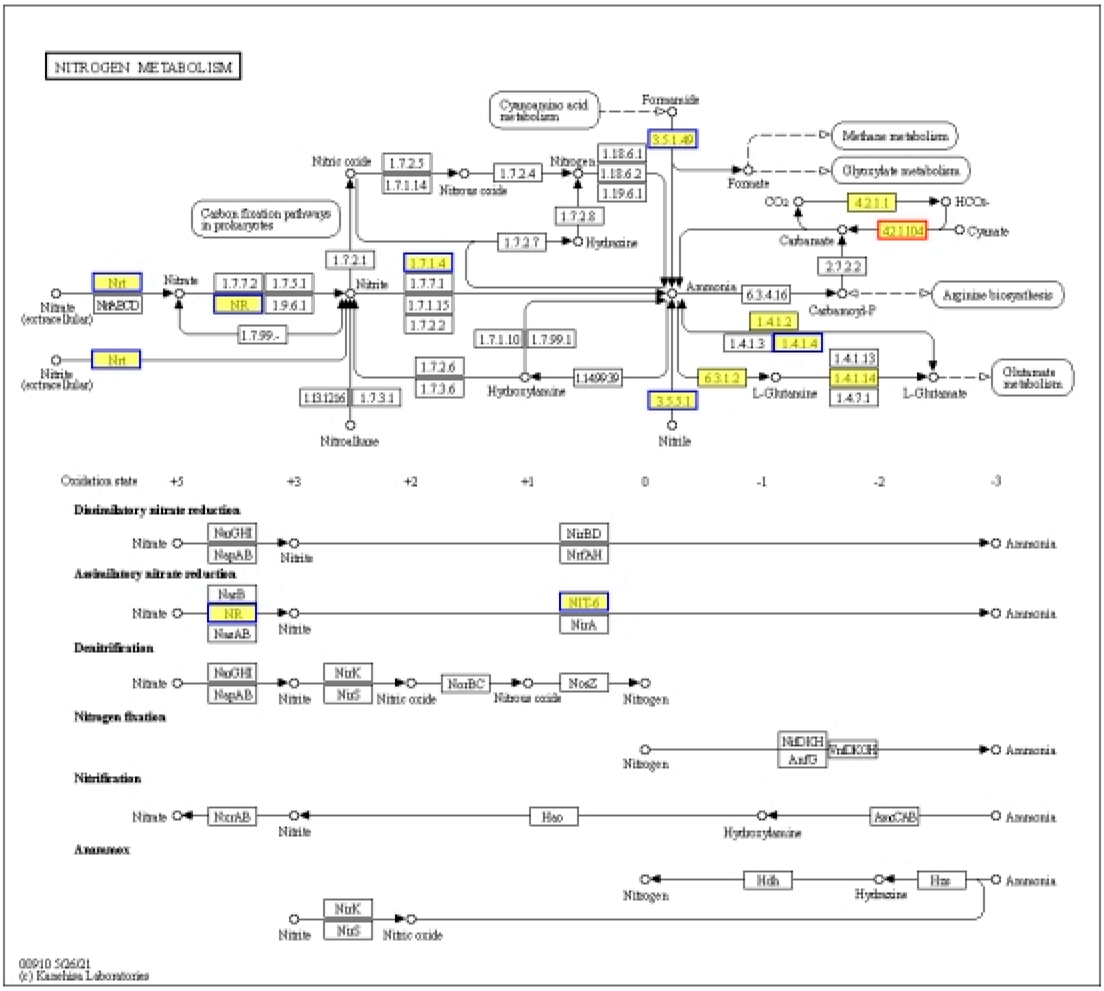

Figure S75 Nitrogen metabolism KEGG pathway analysis of DEGs.

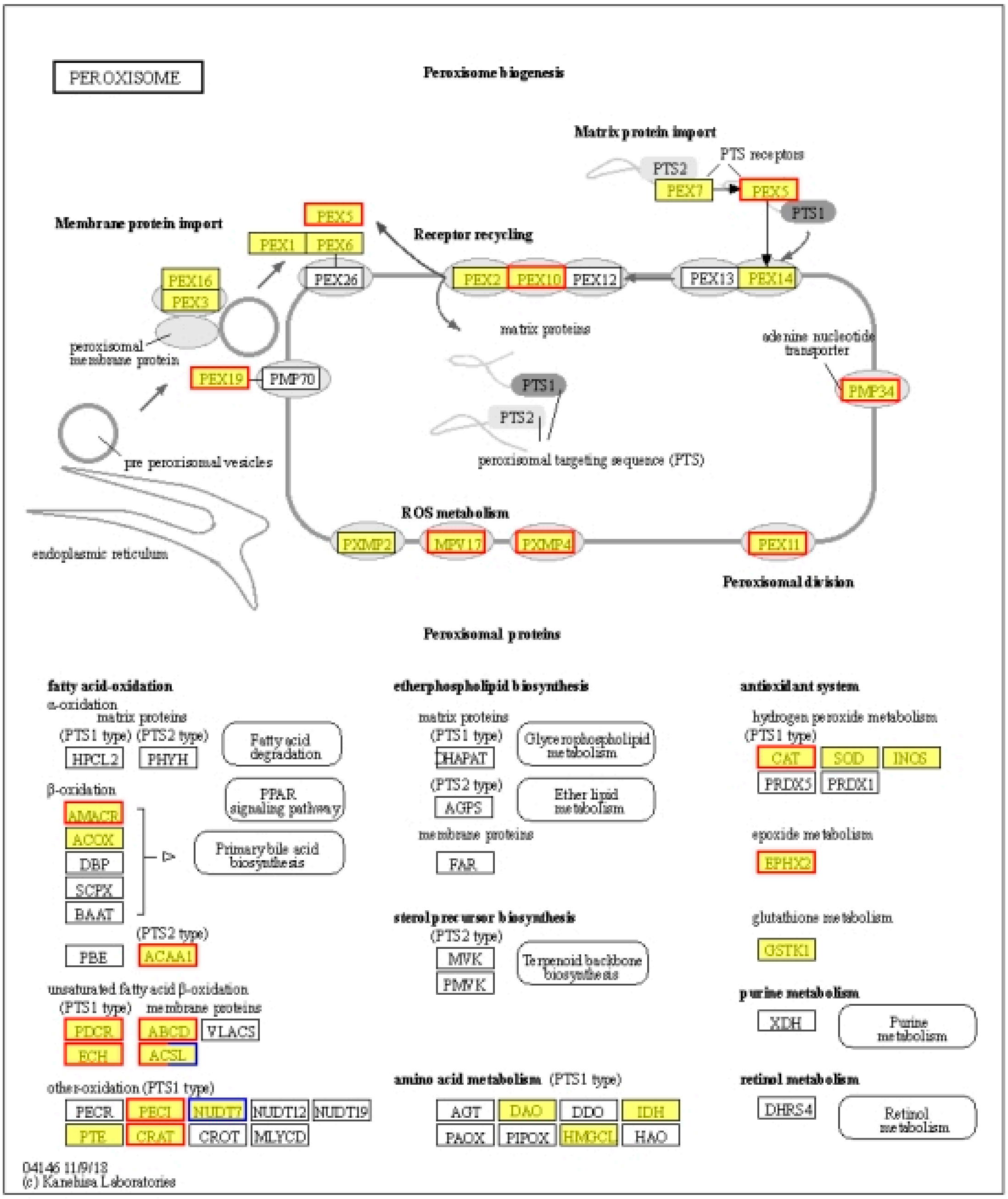

Figure S76 Peroxisome KEGG pathway analysis of DEGs.

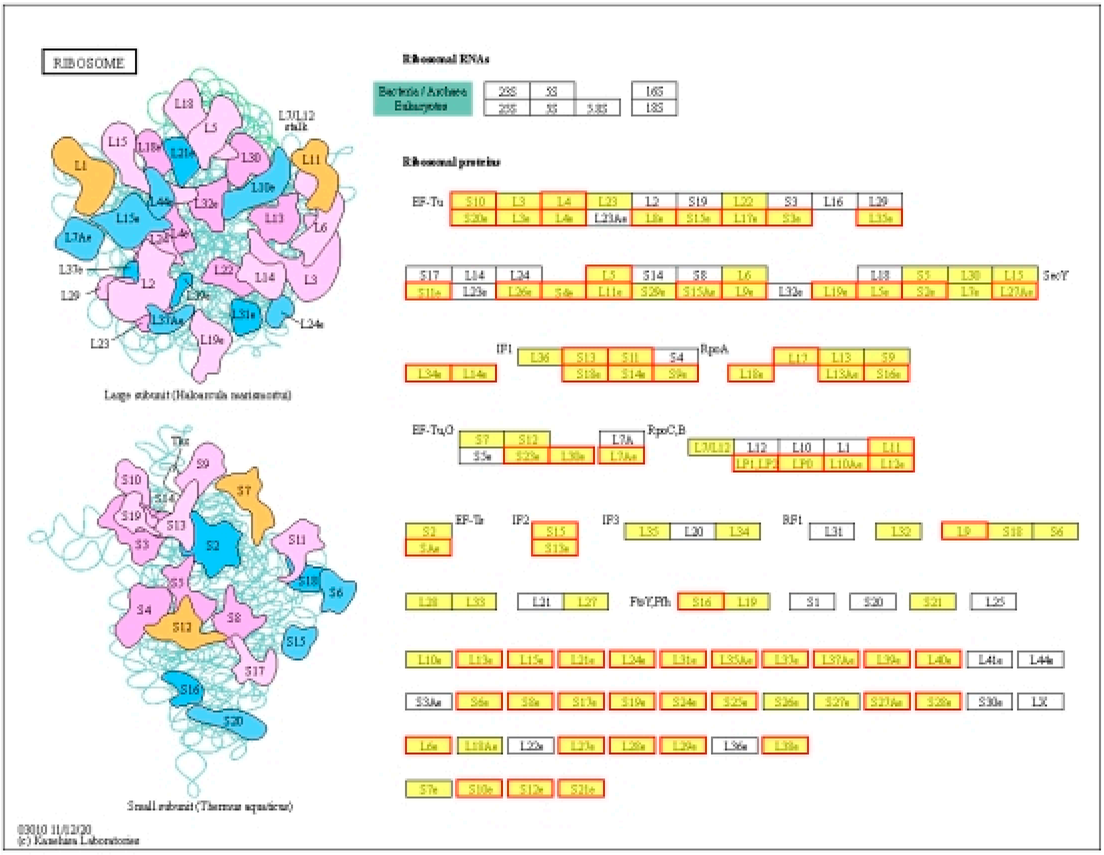

Figure S77 Ribosome KEGG pathway analysis of DEGs.

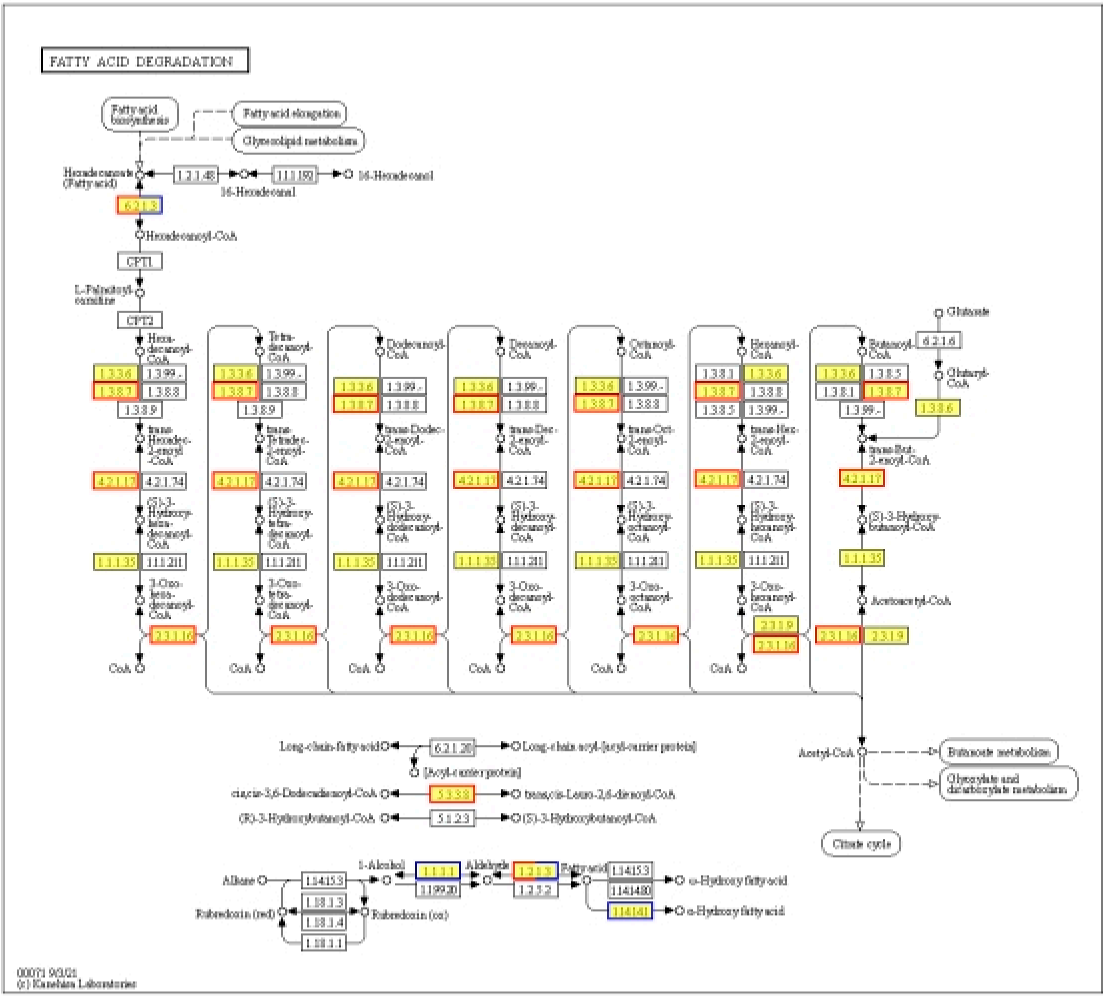

Figure S78 Fatty acid degradation KEGG pathway analysis of DEGs.

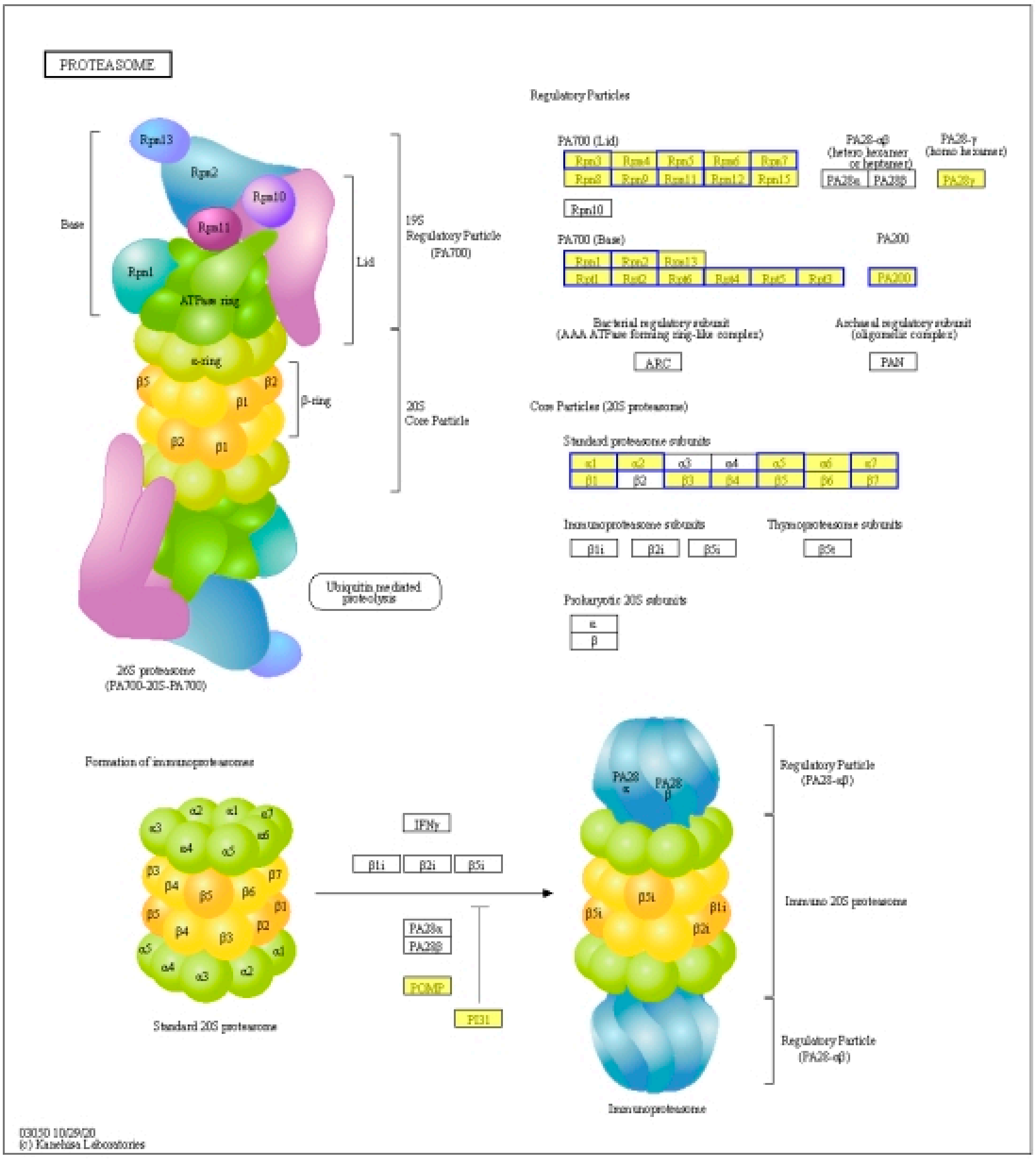

Figure S79 Proteasome KEGG pathway analysis of DEGs.
